# Low functional vulnerability of fish assemblages to coral loss in Southwestern Atlantic marginal reefs

**DOI:** 10.1038/s41598-022-20919-9

**Published:** 2022-10-13

**Authors:** André L. Luza, Juan P. Quimbayo, Carlos E. L. Ferreira, Sergio R. Floeter, Ronaldo B. Francini-Filho, Mariana G. Bender, Guilherme O. Longo

**Affiliations:** 1 Departament of Ecology and Evolution, Universidade Federal de Santa Maria, Santa Maria, Rio Grande do Sul Brazil; 2 Centre for Marine Biology (CEBIMar), Universidade de São Paulo, São Sebastião, São Paulo Brazil; 3 Department of Marine Biology, Universidade Federal Fluminense, Niterói, Rio de Janeiro Brazil; 4 Departament of Ecology and Zoology, Universidade Federal de Santa Catarina, Florianópolis, Santa Catarina Brazil; 5 Department of Oceanography and Limnology, Universidade Federal do Rio Grande do Norte, Natal, Rio Grande do Norte Brazil

**Keywords:** Ecology, Ecology

## Abstract

Marginal reefs sustain coral assemblages under conditions considered suboptimal for most corals, resulting in low coral abundance. These reefs are inhabited by numerous fishes with a generally unknown degree of association with corals that might lead to the assumption that corals play minor roles in determining fish occurrence, when corals could be actually sustaining diverse and resilient assemblages. Using site-occupancy models fitted to data of 113 reef fish species of different life stages (adults and juveniles) from 36 reefs distributed across the Southwestern Atlantic (0.87–27.6°S) we first assessed fish assemblage’s response to coral and turf algal cover, and identified coral-associated fish. Then, we simulated the loss of coral-associated fishes and contrasted it with random losses, providing inferences on the resilience of fish assemblage’s functional trait space to species loss. The entire fish assemblage responded more positively to coral than to turf algae, with 42 (37%) species being identified as coral-associated fish. The simulated loss of coral-associated fish reduced up to 5% the functional trait space and was not different from the random loss. These results reveal that marginal reefs of Southwestern Atlantic reefs host resilient fish assemblages that might preserve fundamental ecological functions and ecosystem services even with coral declines.

## Introduction

Despite covering a tiny portion of the Earth’s surface (0.000063%), coral reefs provide unique conditions and resources for an impressive amount of marine life^1^. Marginal reefs—i.e., those that are under conditions considered suboptimal for most corals including high turbidity and nutrient content^2,3^—are not an exception. Healthy coral colonies form the frame of most tropical coral reefs^4^ and, even at low abundance levels, add structural complexity to marginal reefs, including rocky reef habitats^5,6^. Even small coral colonies can attract reef fishes by providing suitable sheltering, breeding and foraging microhabitats^7–9^, being key components in reef ecosystems and contributing to the goods and services provided by reefs to humans worldwide^4,10–12^. Despite their ecological and economic importance, reefs are subject to local and global threats, including human-driven ocean warming and acidification^4,8,10–14^. These threats are causing mass coral mortality, reducing reef complexity, niche and refuge availability, and influencing a range of other interactions supported by corals^11,15–19^. While coral decline is a global process, its impacts on ecosystem functioning are more documented in coral-dominated reefs^8,9,18–21^. Less studied marginal reefs with lower coral cover may also be in jeopardy as corals’ contribution to sustain diversity may be disproportionately high (i.e., corals as keystone species^22^).

A recent global projection suggests an alarming scenario of halved fish diversity on coral-depleted reefs^20^. However, marginal reefs were not properly represented in that study, despite its widespread distribution^2^ and local importance to biodiversity^3,6^. Fish vulnerability can be particularly high in marginal reefs where the few and locally sparse coral species may have a key role in supporting trophic^17^ and non-trophic^15^ interactions between fishes and benthos, as well as adding structural complexity and concentrating biodiversity^5,23,24^. In tropical reefs of the Indo-Pacific and Caribbean, fishes showing association with corals (namely “coral-dwelling fishes” and “coral-reliant fishes”) are generally numerous and have strict association with corals^5,9,25^. The loss of these fishes, triggered by coral declines, have caused cascading effects on tropical reefs and decreased reef functions and services^8,9,21^. Nonetheless, even basic parameters, such as the identity of fish associated to corals and the influence of coral-associated fish to the functional structure of assemblages, are yet to be determined in most marginal reefs worldwide.

A useful framework to measure the influence of coral declines on the functional structure of reef fish assemblages is combining statistical models^20^ with a functional trait space approach^26^. Under this framework it is possible to project an assemblage’s functional trait structure in a multivariate space and estimate the area filled by such traits (i.e., its functional diversity), which then serves as a reference for comparing reductions in trait space along the simulated loss of coral-associated fish from the assemblage^20^. Alteration in the trait space from species loss has been considered a proxy of assemblage’s resilience (or the lack of it) to disturbances through functional redundancy—an ecological ‘life insurance’ under which ecological functions are ensured by species that share similar traits^27,28^.

Marginal reefs are distributed throughout the Southwestern (SW) Atlantic and developed under turbid and nutrient- and sediment-rich waters^5,29^. Most of these reefs are not primarily built by corals, but sustain significant coralline assemblages that tolerate these conditions amid turf algae, the predominant benthic component in this region^6,30,31^. Coral richness and cover are lower compared to Caribbean and Indo-Pacific reefs, with fewer branching and higher dominance of massive corals^5,30^, although there is a considerable proportion of endemic fishes and corals in this region^30–34^. Evidences of fish association to coral cover (e.g.,^7,9,20,21^) are scarce in the SW Atlantic, likely underestimating the impacts and risks to these reef ecosystems from human-driven coral declines. While existing evidence show that fishes in SW Atlantic marginal reefs use coral colonies to set territories, rest, scape from predators, map resources and reproduce^23,24,35^, it is unclear whether these fish are more likely to occupy reefs with higher coral cover (but see Coni et al.^24^ for an assessment using raw fish counts).

**Figure 1:**
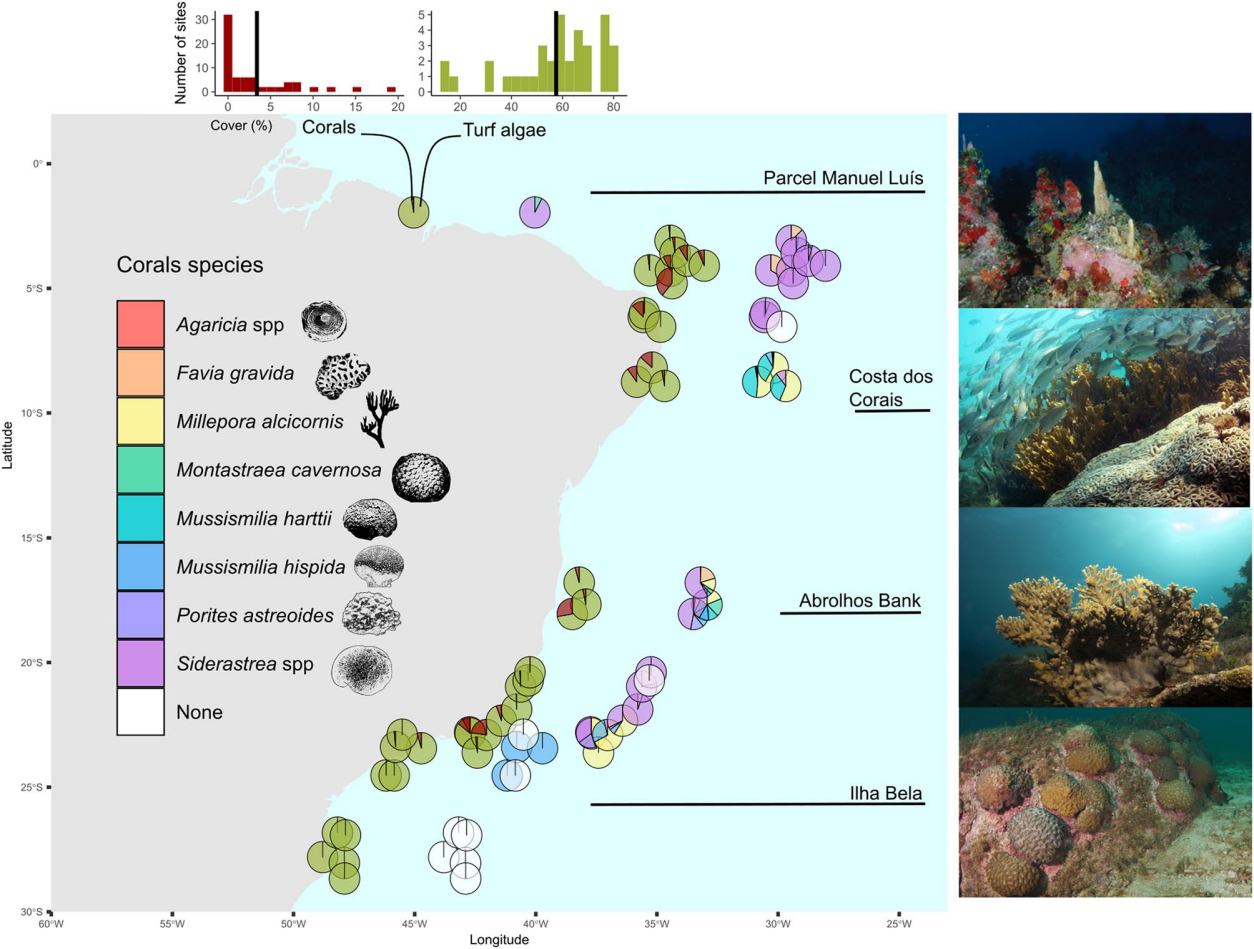
Coral cover in sites where reef fishes were sampled using video plots. Pie charts in the left show the percentage of coral (red) and turf algae cover (green) across sites, and the right pie charts represent the contribution of eight coral species to coral cover in each site. Pie charts were subtly jittered to improve visualization. At the top we presented the percentage and average coral and turf algae cover across sites. We show subaquatic pictures of some sites with marginal reefs. Subaquatic images were kindly provided by Luiz Rocha (Parcel Manuel Luis), Guilherme Longo (Costa dos Corais), Ronaldo Francini-Filho (Abrolhos Bank) and Léo Francini (picture from Alcatrazes, which is spatially close and have similar coral composition to llha Bela). This figure was produced using R v.4.1.2 (https://www.r-project.org/) and edited using Inkscape v.1.0 (https://inkscape.org/).

We investigated whether the loss of coral-associated fish, triggered by coral decline, would influence fish functional diversity in SW Atlantic reefs. To achieve this goal, we first estimated the association of 113 reef fish in juvenile and adult life stages to coral and turf cover using site-occupancy modeling^36^, which accounted for imperfections in fish detection while estimating coral-fish association. Our models accommodate the response of fish to coral and turf algae, the ontogenetic effects on coral-fish association, and the uncertainty in fish observation process. After estimating coral-fish association to the cover of eight reef-building corals (Fig. 1) we built an assemblage-wide functional trait space based on six traits that could confer responsiveness to coral declines and climate change: body length, trophic level, aspect ratio, group size, maximum of preferred temperature and depth^7,9,37^. These traits represent important dimensions in reef fish life-history^38^ and are linked to key ecosystem processes and services that could be impaired by coral decline, such as nutrient transport, cycling and storage in reef areas^39^. We computed reductions in the complete functional trait space along scenarios of primary loss of (i) all coral-associated fish (hereafter ‘total loss’), and (ii) all fish associated to a specific coral species (hereafter ‘loss per coral species’). These scenarios were then compared to random-loss scenarios, making it possible to estimate whether (i) the loss of coral-associated fish would produce greater reductions in the functional trait space than the loss produced by chance, and (ii) there is functional resistance in this system whereby the loss of coral-associated fish, or the loss of fish associated to a particular coral, would not reduce the complete functional trait space. Considering the association between reef fish and coral cover, we expected that: (i) juvenile fish would show a stronger response to coral cover compared to adult fish, since juveniles would strongly rely on corals to protect from predators and acquire food^7,8,40^; (ii) the loss of coral-associated fish in marginal reefs of the SW Atlantic, which host species-poor fish assemblages with limited functional redundancy compared to tropical reefs^38,41^, would reduce the assemblage functional trait space.

## Methods

### Study area

We used benthic and fish data collected in coastal-wide missions conducted along the Brazilian coast from 2011 to 2014 (SISBIOTA-Mar^17,30^), covering 36 sites and ~ 27 latitude degrees, from 0.87°S to 27.6°S (Fig. 1). The Brazilian coast extends across ~ 8.000 km and lies within the Brazilian marine biogeographical province^32,33,42^. There is a longstanding separation of Brazilian from Caribbean and Western Africa provinces by biogeographical barriers (Amazon-Orinoco and the Plata River’s plume, ocean currents, mid-Atlantic depths) so that the endemism levels are considerably high in Brazil^32–34^. Brazilian reefs are arranged along a broad latitudinal gradient of temperature and productivity^32,34^, and develop under turbid and nutrient-rich waters due to large sediment discharge in estuaries (e.g., Rio Doce River). These conditions are ideal for algal development and suboptimal for most coral species. However, corals within the Brazilian province thrive under these conditions and co-exist with high algal cover^5,29,30^.

### Coral cover data

We used data from photoquadrats to obtain coral and turf algal cover per site^30^, considering a ‘site’ as a unique combination of locality, place, and depth category (1–7 m; 8–15 m) in the dataset. These depths represent the depth range at which most SW shallow reefs occur, whereas the depth of 7–8-m a turning tip in abundance of several benthic organisms^30^. Some places had photoquadrats in both or only in a single depth category. Between one and 15 photoquadrats (average 6.3 ± 4.27 SD) were deployed per site, and were predominantly disposed at the reef top and in places sheltered from wave action. The photoquadrats were subdivided in 5 subunits of 25 cm^2^ where pictures were taken. Coral and turf algal cover were quantified by laying fifty random points in each subunit^30^. The data of coral and turf algal cover used in site-occupancy models were site-level averages across the photoquadrats.

The cover of eight reef-building corals with non-zero cover in ≥ 4 sites were associated to fish occurrence probability (Fig. 1): one branching hydrocoral, *Millepora alcicornis* (Linnaeus, 1758), six massive scleractinian corals: *Favia gravida* Verrill, 1868, *Mussismilia hispida* (Verrill, 1901) and *M. hartii* (Verrill, 1868)—which can be facelloid depending on phenotypical variation, *Montastraea cavernosa* (Linnaeus, 1767), *Porites astreoides* Lamarck, 1816, *Siderastrea* spp. Blainville, 1830, and one plate coral *Agaricia* spp. Lamarck, 1801. Among them, two could not be precisely identified to the species level through the pictures: (i) *Siderastrea* spp. which embraces the cover of starlet massive corals (*Siderastrea stellata, Siderastrea radians*, *Siderastrea siderea*, but mostly *S. stellata*), and (ii) *Agaricia* spp. which embraces the cover of hat corals (*Agaricia fragilis*, *Agaricia humilis*, and one unidentified *Agaricia* sp.). Prevalent turf algae comprise filamentous algae, articulated coralline algae, red (e.g. Gelidiacea), brown algae (e.g. small *Lobophora* spp. or even *Sargassum* spp.) and complex epilitical algal matrix with detritus and associated cryptofauna^30^.

### Fish data

We used GoPro® cameras to record fish activity within the same 2 m^2^ areas of benthic sampling (see^17^). Video plots recorded fish activity during 10 min, an effort that allows an adequate characterization of dominant species in the fish assemblage^43^. Between one to 12 video plots were deployed per site (average of 4.84 ± 2.56 SD). Despite being deployed in the same areas, fish and benthic sampling were planned for independent research^17,30^ thereby the sampling imbalance shows that fish were not monitored in all plots with benthic sampling.

Through these videos we obtained a history of observations (detection = 1, non-detection = 0) of each fish species of a given life stage (juvenile or adult) in each site and frame of 10 min of video recording. Analyzes covered 113 out of the 140 species detected in these studied sites by Longo et al.^17^, and comprised only fish identified at the species level and occurring in at least one site. Juvenile and adult fish were identified based on size estimates of individuals recorded in the videos. We considered as adults all individuals measuring > 5 cm for species with total length (T_L_) between 8 and 16 cm, all individuals measuring > 10 cm for species with T_L_ > 16 cm, and all individuals of species with T_L_ < 5. We considered as juveniles all the individuals measuring ≤ 5 cm for species with T_L_ between 8 and 16 cm, and all individuals measuring ≤ 10 cm for species with T_L_ > 16 cm (T_L_ data were obtained from Quimbayo et al.^44^). In certain cases, differences in fish body sizes also reflect different color patterns among the adult and juvenile development phases. Among the 113 species, 112 were recorded at adult life stage, 47 at juvenile life stage, and 46 at both stages.

### Statistical analyses

#### Measuring coral cover influence on fish occurrence

We fitted site-occupancy models to the historic of fish observation, in a Bayesian-Inference framework, to estimate the effect of coral and turf algal cover on fish occurrence probability. Site-occupancy modeling allows to estimate the effect of site covariates on species occurrence probability through a model of site occupancy, in which coral and turf cover were treated as fixed effects and fish identity as a random effect. The random effect allowed that (i) species show identical, but not any possible and independent, response to coral and turf cover, and (ii) species with many detections help improve the performance of models fitted to species with less data^36^. Uncertainty in the process of fish observation was assessed through an observation model^36,45^ whereby a spurious relationship between fish occurrence and coral and turf cover was avoided by treating non-detections as imperfectly observed quantities^36,45^. As data were acquired in samples replicated across space rather than time—a common characteristic of marine datasets^45^—we used the “time-for-space substitution” strategy in the observation model where each 10-min frame was considered one sampling occasion within each site. Detection probability model had depth as a fixed effect and video plots as a random effect in order to account for variation in detection induced by distributing video plots in space (see model details in Supporting information file S2). Variation in detection probability across fish, averaged across models of coral species, was analyzed relative to maximum body size, depth, and fish life stage (see Supplementary information file S2).

The standardized effect size of regression coefficients (β_k_) and associated Credible Intervals (CI), estimated across *k* = 1 to *K* species, were the parameters used to analyze assemblage-wide and species-specific response of fish to corals and turf. The coefficient depicts the effect strength of each coral species cover (β_1k_) and turf cover (β_2k_) on the occurrence probability of each fish *k*. β_1k_ values were often larger than β_2k_ because the average and standard deviation (SD) of coral cover was very low (< < 1%) and, as the regression coefficients are in units of SD from the mean, any increase in coral cover would lead to a steep β_1k_. Parameters were estimated by running eight different models per life stage class, in which turf algal cover and one coral species cover were included as fixed effects per run.

The averages of β_1k_ and β_2k_, estimated across the *K* species, were used as an indication of assemblage-wide response of fish to coral and turf cover. Averaged estimates of β_1k_ and β_2k_ were projected into ridgeline plots, which show the density of estimates across 3,000 posterior probability samples produced by three parallel Monte-Carlo Markov Chains (Supporting Information file S2). We then counted the number of fishes presenting positive, neutral, and negative response to these covariates using the species-specific estimates of β_1k_ and β_2k_. Fish presenting positive response to coral and turf were those with lower β_1k_ and β_2k_ CI bounds > 0, respectively. In contrast, fish presenting negative response to coral and turf were those with upper β_1k_ and β_2k_ CI bounds < 0, whereas fish with neutral response had lower and upper β_1k_ and β_2k_ CI bounds superimposing zero. The proportions of fish per category of response to coral and algal cover were presented in bar plots (one bar plot per covariate (coral, turf) and life stage (adult, juvenile)). Finally, we used β_1k_ and β_2k_ estimates to identify ‘coral-associated fish’ and apply them in fish loss scenarios (see below). “Coral-associated fish” were the fish presenting positive β_1k_ with associated CI not superimposing the zero, and β_2k_ ≤ 0. The regression coefficients and CI for adult and juvenile coral-associated fish were shown through coefficient plots (see the complete framework in Fig. S1.1). Coefficient plots also showed the Bayesian P-Value (BPV), a goodness-of-fit statistic of site-occupancy models which shows whether the model can estimate data similar to the input data, so that a BPV ~ 0.5 indicates a well-fitted model^36^.

#### Simulated effect of coral declines on fish functional diversity

A complete functional trait space was built with 113 species and six traits: total length, trophic level, aspect ratio, group size, maximum of preferred temperature and depth (Supplementary information file S3, Table S3.1). All traits, except for total body length that was obtained directly from the video estimates, were gathered from Quimbayo et al.^44^. Total body length, aspect ratio, trophic level, maximum preferred temperature and depth were treated as quantitative traits, whereas group size was treated as an ordered trait (schooling fishes had higher ranks than solitary fishes). Functional trait space area was calculated through the convex-hull approach of Cornwell et al.^26^, Villegér et al.^46^ and Maire et al.^47^ (see Supporting information file S3 for a detailed protocol).

We simulated the impacts of total coral loss and loss of each coral species on reef fish functional diversity by simulating the complete removal of coral-associated fish (assuming they would vanish without corals) and computing the reduction in trait space relative to the complete trait space. Reductions in the functional trait space area (FS_reduced_) along each scenario were compared to the complete trait space area (FS_complete_) using the Eq. (1)

1 $${RFS}=\left(1-\left({FS}_{reduced}/{FS}_{complete}\right)\right) \times 100$$

where the total Reduction in the Functional trait Space area (RFS) is the inverse of the ratio between FS_reduced_ and FS_complete,_ multiplied by 100 to obtain a percentage. An *RFS* of 0% indicates no loss of functional space and, likely, high functional similarity (or redundancy)^41^ among the coral-associated fish being removed and those fish that remained in the assemblage. In contrast, an *RFS* of 100% indicates a complete reduction in the functional trait space and no functional redundancy nor resilience in the system.

Reductions in trait space due to the exclusion of coral associated fish were then contrasted with two scenarios of random loss which involved the random removal (100 runs, with uniform probability of sampling) of (i) the same number of species as the total number of coral-associated fish (42 species), and (ii) the same number of associated fishes per coral species. We also contrasted those scenarios with an alternative randomization procedure [also obeying the rules (i) and (ii)] where species in the vertices of the trait space—and therefore more functionally distinct—had higher loss probability. We explored the degree to which RFS values under the total loss and loss per coral scenarios deviate from random loss using violin plots. These plots show whether the RFS from both scenarios lie inside or outside the interval of random RFS values. All analyzes and figures were produced in R programming environment^48^ using the packages ‘jagsUI’ (site-occupancy modeling^49^), ‘grDevices’ (functional trait spaces^48^), 'base’ (random sampling^48^), and ‘ggplot2’ (figures^50^).

## Results

Coral cover varied from zero to 19.2% across the 36 studied sites, with an average of 4% across sites (Fig. 1). In turn, turf algae were much more prevalent (average cover of 57% across sites) but also variable across sites (15 to 81%) (Fig. 1). While turf prevailed in the South, coral cover prevailed in the Northeast Brazil, around Costa dos Corais, Abrolhos Bank, and Rocas’ Atol areas (Fig. 1). The most widespread corals were *Siderastraea* spp., *Mussimilia hispida*, and *Millepora alcicornis* (detected in 23, 13, and 12 sites, respectively) (Fig. 1). *Siderastrea* spp. had the highest average cover (1.8 ± 3.7% SD, range of 0 to 19.2%), followed by the hydrocoral *M. alcicornis* (1.0 ± 2.1% SD; range of 0 to 10.3%).

We analyzed a total of 194 sampling occasions of fish sampling, with an average and standard deviation of 17.25 ± 24.22 detections per fish species across occasions. The 113 species belong to 41 different families, but mostly from the Scaridae, Labridae, Haemulidae, Pomacentridae, and Serranidae families (Supplementary information file S3, Table S3.1). The most frequently detected adult fish were *Stegastes fuscus* (detection in 118 occasions), *Abudefduf saxatilis* (109 occasions), *Sparisoma axillare* (92 occasions) and *Acanthurus chirurgus* (91 occasions). The most frequently detected juvenile fish were *A. saxatilis* (65 occasions), *Halichoeres poeyi* (40 occasions), *S. fuscus* (33 occasions), and *Sphoeroides spengleri* (27 occasions). Fish detection probability decreased with fish total length (an effect similar for adult and juvenile fish), and it was generally higher at depths of 8–15 m. than at 1–7 m (Supplementary information file S2, Fig. S2.1).

### Fish response to coral and turf algal cover

**Figure 2:**
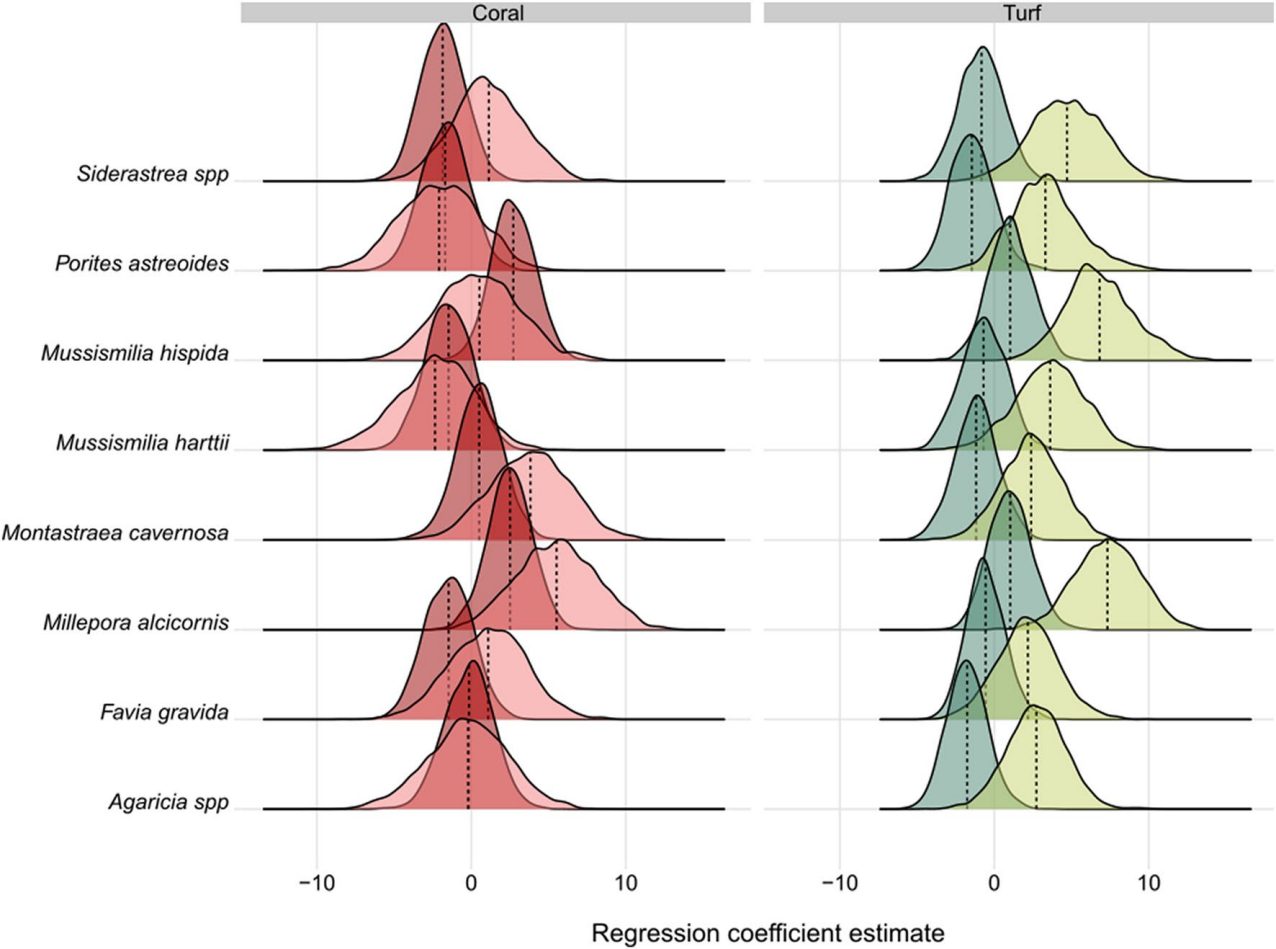
Assemblage-wide response to coral cover and turf algae. Ridgeline plots showing fish assemblage response to coral cover (red) and turf algae (green). The assemblage-wide response was obtained by averaging the parameters β_1k_ and β_2k_ across the 107 adult (darker colors) and 49 juvenile fish (lighter colors). These parameters were estimated through a site-occupancy model fitted to data of the entire fish assemblage in Bayesian-Inference framework. The density of values was produced by considering the whole set of 3000 samples of the posterior probability distribution of the averaged β_1k_ and β_2k_. The vertical dashed line depicts the median regression coefficient across these 3000 posterior probability samples. This figure was produced using R v.4.1.2 (https://www.r-project.org/) and edited using Inkscape v.1.0 (https://inkscape.org/).

**Figure 3:**
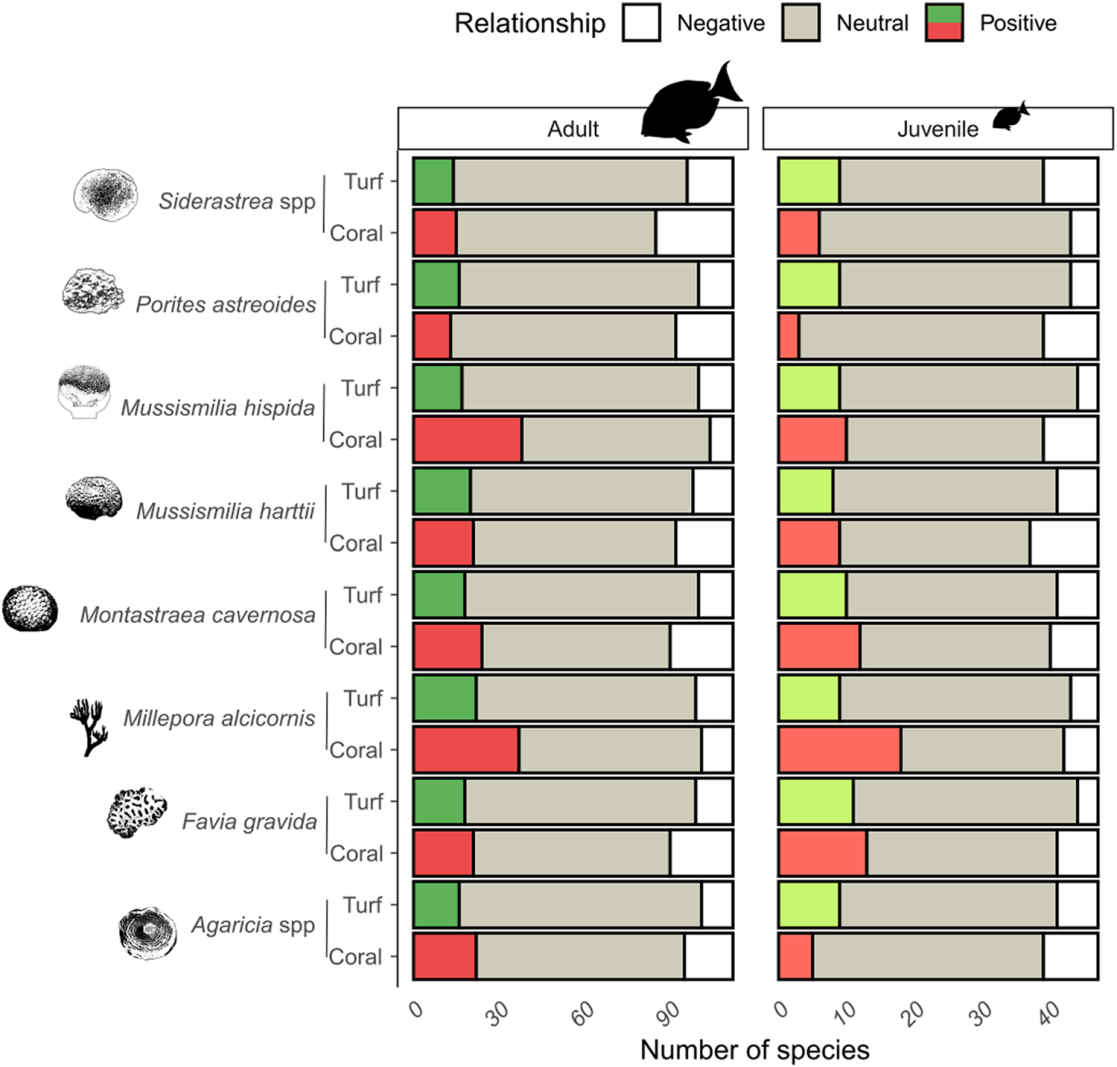
Bar plots depicting fish response to coral and turf cover. Barplots showing the number of fish species showing positive, neutral, and negative response to coral cover and turf cover, for adult and juvenile fish. This figure was produced in R v.4.1.2 (https://www.r-project.org/) and edited using Inkscape v.1.0 (https://inkscape.org/).

Fish assemblage response to coral and turf algal cover varied with fish life stage and coral species (Fig. 2). Juvenile fish presented a more positive response to coral cover than adult fish, except for *M. hispida*, while the response to turf algae was generally negative for adult fish and always positive for juvenile fish (Fig. 2). The assemblage-wide response was more positive (i.e., averaged β_1k_ across species generally higher than zero) when considering *M. alcicornis*, *M. hispida* and *Montastraea cavernosa* covers relative to other corals. A greater number of fish species presented a positive response to coral than to turf cover, and a greater number of fish species presented a neutral relationship to turf algae than to coral cover (Fig. 3).

### Coral-associated fish and scenarios of primary fish loss

**Figure 4:**
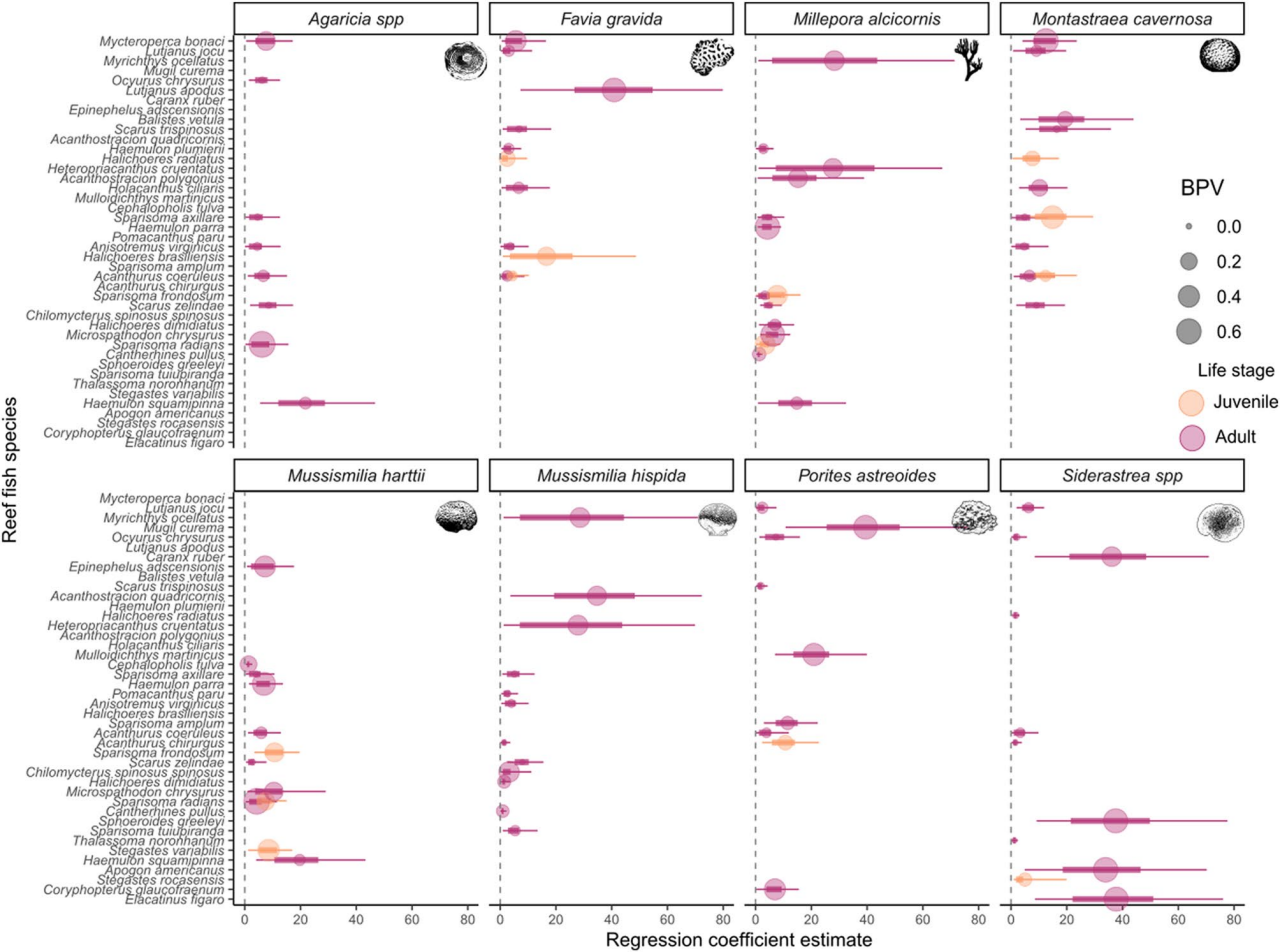
Coefficient plots showing the response of juvenile and adult coral-associated fish to live coral cover, per coral species. The coefficients of regression β_1k_ represent the strength of live coral cover influence on fish occurrence; coefficients for adult fish are shown in dark red, and juvenile fish in orange. Point size represents the Bayesian P-Value (BPV), a goodness-of-fit statistic for models ran in Bayesian-Inference framework (good fit at BPV ~ 0.5). Species are ordered according to their body size along the Y-axis, being *Mycteroperca bonaci* (150 cm) and *Coryphopterus glaucofraenum* (8 cm) the species with the largest and smallest maximum body size, respectively. Coefficient average, 90% and 50% Credible Intervals (points, thin and thick bars, respectively) were produced by analyzing the 3,000 samples of the posterior distribution of β_1k_ across the *K* fish species. The vertical line passing through zero identifies the zone of no effect of coral cover on fish site-occupancy probability. This figure was produced using R v.4.1.2 (https://www.r-project.org/) and edited using Inkscape v.1.0 (https://inkscape.org/).

A total of 42 (37%) out of 113 analyzed species were identified as coral-associated fish. Models presented reasonable to good fit to data (Fig. 4, Supporting Information file S2, Table S2.1), and all estimated parameters had a Rhat statistic lower than 1.1, indicating reliable posterior distributions of estimated parameters across the Monte Carlo Markov Chains. The hydrocoral *M. alcicornis* had the highest number of coral-associated fish among the eight analyzed coral species (Figs. 3 and 4). For coral-associated fish with individuals at both juvenile and adult stages, we found that juvenile fish generally had a more positive response to coral cover than adult fish—as it was the case of *Sparisoma radians*, *S. axillare*, *Acanthurus coeruleus*, and *Scarus zelindae* (Fig. 4). *Millepora alcicornis*, *Montastraea cavernosa* and *Favia gravida* were the corals with the highest number of coral-associated juvenile fish, and *M. alcicornis* and *M. hispida* were the corals with the highest number of coral-associated adult fish (Figs. 3 and 4).

**Figure 5:**
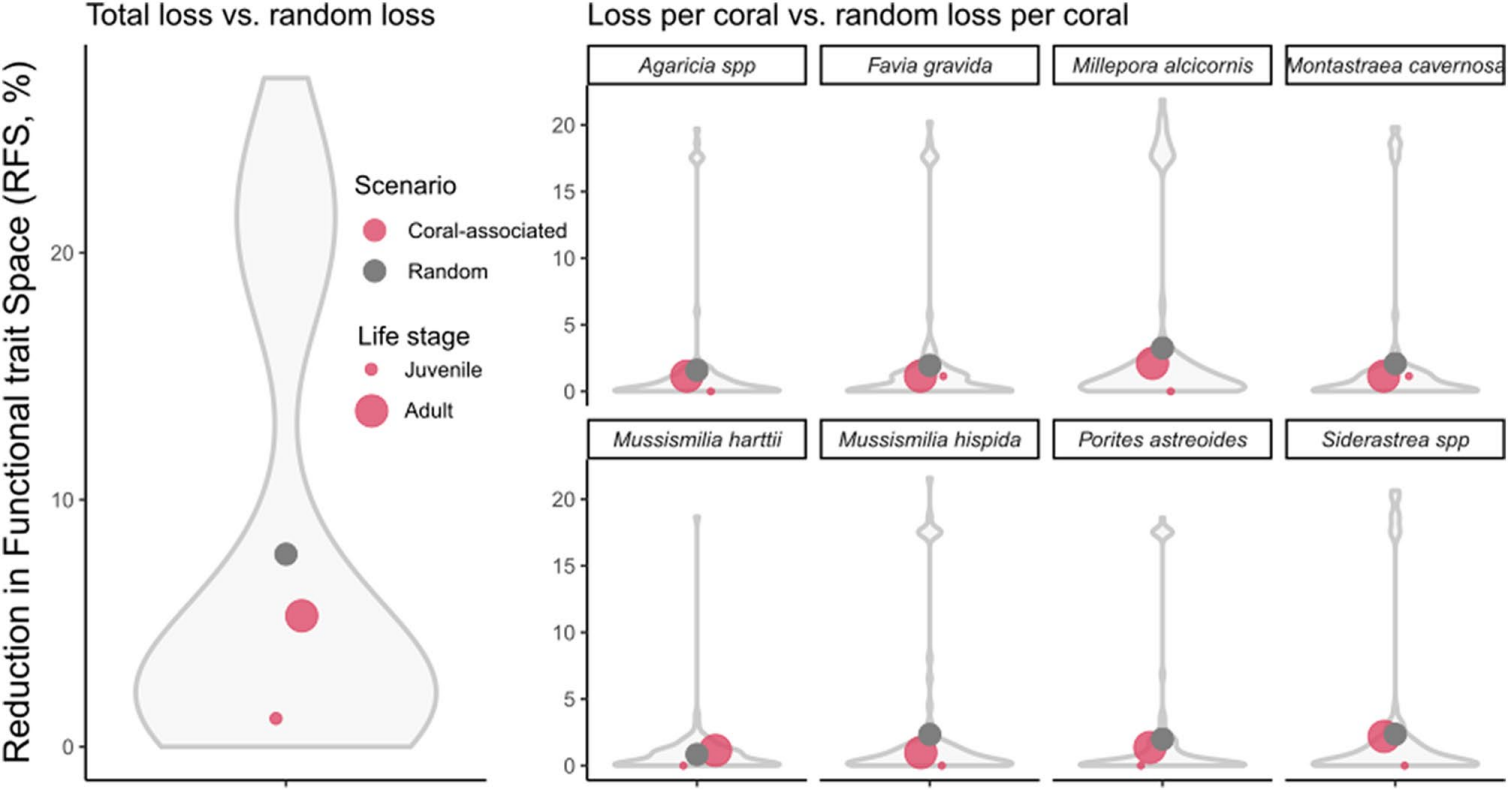
Reduction in the Functional trait Space area (RFS) between scenarios of loss of coral-associated fish and random loss. The effect of coral declines on functional space was analyzed by building spaces based on four fish traits (group size, maximum body size, trophic level, aspect ratio), and then comparing the total loss and loss per coral of coral-associated fish with the simulated losses, for adult and juvenile fish. Points depicted the average for each scenario and fish life stage. This figure was produced using R v.4.1.2 (https://www.r-project.org/) and edited using Inkscape v.1.0 (https://inkscape.org/).

We found that the removal of coral-associated fish had minimal influence on the complete (assemblage-wide) functional trait space. The reduction in functional space in the scenario of total coral loss was 5.30% for adult fish and 1.14% for juvenile fish, whereas the average reduction caused by a random loss was 8.09 ± 8.54% (Fig. 5). Reduction caused by the scenario of loss per coral species was even lower (0 to 2.19%), with values laying within the interval of random reductions (Fig. 5). The small reduction in functional space with the removal of coral-associated fish was particularly caused by the removal of *Acanthurus*, *Acanthostracion*, *Sparisoma* and *Caranx* tropical fish genera with traits such as high maximum preferred temperatures, low maximum depth, and large group size (Supporting Information file S3, Fig. S3.2). The alternative randomization procedure, where most functionally distinct species were more likely to be lost, resulted in a loss of functional space around four times greater than the observed loss (Supporting Information file S3, Fig. S3.3).

## Discussion

We found that Brazilian reef fish assemblages had a positive response to coral cover, implying in higher and lower probabilities of fish occurrence with increasing and decreasing coral cover, respectively. These findings underscore the role of corals as keystone species^22^ to sustain fish populations in marginal reefs of the SW Atlantic, despite occurring in relatively low abundances (4–20%) and coping with the dominance of algal turfs^30^. Coral-fish association was observed for both juveniles and adult fish, varying according to coral species coverage. Turf algae positively influenced juvenile fish, possibly acting as important foraging areas. Finally, the simulated removal of coral-associated fish did not compromise the range of delivered ecological functions, revealing that Brazilian reef fish assemblages are somewhat resilient to the primary loss of coral-associated fish.

If fish assemblages respond to the most abundant benthic resource on reefs, we would expect SW Atlantic reef fish assemblages to present a positive relationship with turf algae and less so with corals. Our results indicate the opposite: a greater number of fish species showed a positive relationship with corals than with turf algae, revealing the keystone role of coral species^22^ through their positive effect on the occurrence of fishes. As expected, adults were less influenced by corals than juveniles, whom, in their turn, were more influenced by turf algae. Turf algae dominates SW Atlantic reefs^30^ but add low complexity to reef habitats when compared to corals^51^. In reefs where algal turfs are thicker, they could function as important foraging habitats for juvenile fish, or still nursery areas when algal turfs are also associated with corals or arborescent benthic invertebrates (e.g. sponges, bryozoans), conferring easy access to multiple food sources^7^. Many fish species can perform daily and ontogenetic migrations from these algae-dominated habitats, seeking habitats with higher coral cover^52^, and therefore having a critical role in cross-habitat nutrient transport, thus promoting nutrient cycling and reef fertilization^39^. This might be the case, for instance, of herbivorous species such as surgeonfishes and parrotfishes (*Acanthurus coeruleus, A. chirurgus,* and *Sparisoma axillare*) which were identified here as coral-associated fish but found abundantly in algae-dominated habitats (e.g., *Sargassum* beds)^40^. In the adult stage, fish usually set territories, scape from predators and reproduce nearby corals and often have their distribution restricted to the vicinity of coral colonies^7,15,24^. However, corals with different morphologies might provide distinct habitats for fish^7^. For instance, the largest number of juvenile and adult coral-associated fish was detected for the branching hydrocoral *Millepora alcicornis*, whose branching structure add reef complexity and can be used by fish for sheltering and food acquisition^24,35^. Such complexity delivered by *M. alcicornis* to SW Atlantic reefs is threatened by ocean warming, presenting high mortality after consecutive massive bleaching events^53^. Also, this species is highly susceptible to benthic competitors that provide lower structural complexity, potentially impacting coral-associated fish occurrence^54^. While branching corals are indisputably important for fish, SW Atlantic reefs are dominated by massive corals^6,30,31^. Among them, *Mussismilia hispida* and *Montastraea cavernosa* were of high importance for fish. *Mussismilia hispida* is a widespread coral that forms extensive colonies, mainly in rocky reefs of southeastern Brazil^55^. *Montastraea cavernosa* is widespread in tropical reefs, and can form reef boulders with a disproportionately high cover below 20 m^56^. In the Caribbean, for instance, several juvenile fish find shelter and feed close to the convolutions of *Montastraea*^8^. These spatially extensive habitats, build up by the growth of massive corals such as *M. cavernosa*, are used by numerous reef fish throughout the SW Atlantic^57^.

In climate change scenarios, coral declines are expected to cause a non-random or trait-biased fish loss, with sedentary, small ranged and coral associated fish being more likely to disappear in the near future^20^. A recent estimate suggests that coral declines would cause tropical reefs to lose 50% of their reef fish richness and 23% of their fish functional entities^20^. In fact, the loss of species does not parallel with the loss of functional diversity, mainly because fish assemblages are characterized by overall functional redundancy^27,41^. We detected that a 37% loss of fish richness would merely cause a 5% loss in functional diversity. This challenges the view of low functional redundancy in SW Atlantic reefs by showing that functionally redundant fish, in a species-poor assemblage, could still buffer the loss of coral-associated fish in the region. The loss of functional diversity per coral species was even lower, which possibly means that fish associated with other corals could fill the functional space of fish associated to specific coral species.

Three processes might explain the functional redundancy and resilience found here. First, every fish assemblage seems to have an inherent redundancy and therefore a degree of resilience to disturbances. Recently, it was found that both tropical and temperate reefs might host a persistent core of key ecological functions, with a varied degree of functional redundancy across space^38,41^. Despite its limited redundancy, SW Atlantic marginal reefs might still tolerate primary extinctions of coral-associated fish as functions could be secured by species that are less dependent on corals^41^. Second, the SW Atlantic region lacks obligate corallivorous fishes, with most species presenting wide geographic distribution, generalist diets and habitat uses^33,58^. Coral assemblages in SW Atlantic reefs are dominated by massive corals^29,30^ that do not seem to promote similar chances for diversification in comparison to those offered by branching corals in Indo-Pacific and Caribbean reefs^65^, potentially limiting morphological and feeding specialization, leading to lower functional diversity. Biting on prey attached to or in between coral branches and within reef crevices boosted the jaw and body shape differentiation among reef fish in Indo-Pacific and Caribbean reefs^65^. This macroevolutionary process seems to not have occurred or occurred to a lesser extent in SW Atlantic reefs. SW Atlantic reef fish feed on invertebrates, the epilitical algal matrix and detritus on the surface of massive corals through suction and/or biting, seldom feed on corals^58,66,67^. This process is even observed in the reef fish family Chaeodontidae, globally recognized for their high trophic specialization and strong association with corals^65^. Therefore, the functional loss in SW Atlantic reef fish assemblages caused by reliance on corals and mortality of branching corals is likely to be less severe than those projected for the Indo-Pacific and Caribbean, where there is a greater number feeding specialist fishes^9,18,19,59^. Third, despite being considered an isolated outpost of the diverse Caribbean reefs, Brazilian reefs host a second center of diversification within the Atlantic biogeographic region^32–34^ and, for that reason, evolutionary processes may have caused lineage diversification and the accumulation of functional redundancy over time.

We identified that Brazilian reef fish assemblages are likely to maintain its range of ecological functions despite climate-induced coral declines and consequent loss of coral-associated fish. However, scenarios for more diverse reefs often show functional loss with coral declines^20,25,59^. Our approach differs in a fundamental aspect when compared to these other initiatives: we explicitly considered the chance that fish would not be detected in truly occupied sites. It allows a more robust inference on species occurrence-habitat relationship, and more reliable estimates of species distributions in environmentally suitable sites (i.e., species occurring in more sites than suggested by the raw data)^36,45,60^. Yet, we have not accounted for four important aspects of the vulnerability of reef functioning to fish loss. First, we are not certain whether reef functions are already compromised due to declines in reef fish abundance with climate change and local impacts such as overfishing. For instance, using a meta-analytical approach, Wilson et al.^9^ found that 62% out of 55 fishes were coral reliant and showed declines in abundance following coral loss in reefs from six marine biogeographical regions (i.e., Caribbean, Arabian Gulf, Indian Ocean, Indo-Australia, Southern Japan and East Pacific). Also, fish standing biomass in reef food webs facing ocean warming was predicted to severely drop, impairing reef productivity and diversity^61^. Thus, not accessing abundance prevents quantification of the intensity of ecosystem functions. Second, we do not know whether resilience would be maintained under secondary extinctions, where species with trophic interactions with coral-associated fish would be locally extinct. Under this secondary extinction’s scenario, the loss of corals and coral-associated fish could cause a cascading effect on the ecosystem that we could not anticipate or model. In this case, losing corals means losing ecosystem functions and in Brazil, where there is a high endemism level, this also represents erosion of the Tree of Life. Third, although unreported for most reefs in the SW Atlantic, reef area and cover were possibly larger in the past than they are today^62^ due to secular anthropogenic impacts causing the loss of coral cover and reef complexity^6^. Extinctions may have already taken place^63^, and what we currently see is a depleted assemblage of fish and invertebrates. Finally, redundancy might be limited to the major sampled habitats (i.e., shallow depths and horizontal reef tops). As coral species distribute at depths greater than those sampled here^64^, more interactions between fishes and corals such as *Mussismilia hispida* and *Montastraea cavernosa* are likely to be discovered in the future by surveying deeper waters and reef walls^5,64^. These are areas that demand investigation to anticipate the effects of coral declines on marginal reef assemblages.

## Conclusion

We assessed coral-fish association and functional vulnerability of fish assemblages to coral declines, which are overlooked processes underlying the influence of corals on fish occurrence in marginal reefs of the SW Atlantic with low coral cover^2,3^. Indeed, we found corals to be keystone species^22^ in SW Atlantic reefs due to their positive influence on the occurrence of multiple reef fish species despite their low abundance. Corals are nonetheless declining globally due to multiple threats such as ocean warming and acidification, pollution, and overfishing^4,11,12,14,18^, being the reduction in the occurrence of fish strictly dependent on coral cover the most immediate effect of coral declines^9,20,25^. Our simulations highlighted that the collective loss of coral-associated fish would minimally reduce reef fish functional diversity in SW Atlantic. Therefore, SW Atlantic reef fish assemblages might be diverse and redundant enough to be resilient to the loss of coral-associated fishes maintaining critical functions. While trait similarity corresponds to functional redundancy, an insurance against species loss on ecosystems, we are aware that fish species could still differ in other, unmeasured trait dimensions. In such case, species extinctions could cause functional losses to SW Atlantic reef fish assemblages. Exploring a direct causal link between traits and functions through experimental and empirical approaches could strengthen inferences on resilience and reef ecosystems functioning^37,39^ avoiding oversimplifications and underestimates of functional loss. Our results should not minimize the importance of corals neither for fish occurrence nor for the persistence of resilient assemblages. Instead, it represents an opportunity for reef management with a focus on maintaining and improving ecological functioning in scenarios of global change. For instance, considering that several fish species had a positive association with coral cover, would managing reefs to maintain coral cover increase fish biomass and fish stocks? Overall, our study presents an optimistic scenario of relatively low vulnerability of fish assemblages to the loss of coral-associated fish in SW Atlantic marginal reefs.

## Supplementary Information

Supplementary Information.

## Data Availability

We provide all fish and benthic data, as well as R and WinBUGS code ensuring full replicability of analyses, at https://github.com/Sinbiose-Reefs/coral_fish_project.git. All data are available on this GitHub page. Benthic data are also available on Dryad: https://doi.org/10.5061/dryad.f5s90. Stable and standardized versions of the datasets used in this study will be deposited in the OBIS database.
