# Supplementary material for: Low functional vulnerability of fish assemblages to coral loss in Southwestern Atlantic marginal reefs
Source: Sci Rep. 2022 Oct 13;12:17164. doi: 10.1038/s41598-022-20919-9 (PMC9562355; doi:10.1038/s41598-022-20919-9)
Supplement: Supplementary file 1 — Supplementary Information. [file 41598_2022_20919_MOESM1_ESM.docx]

**Supplementary information file S1: Description of the framework, studied sites and species.**


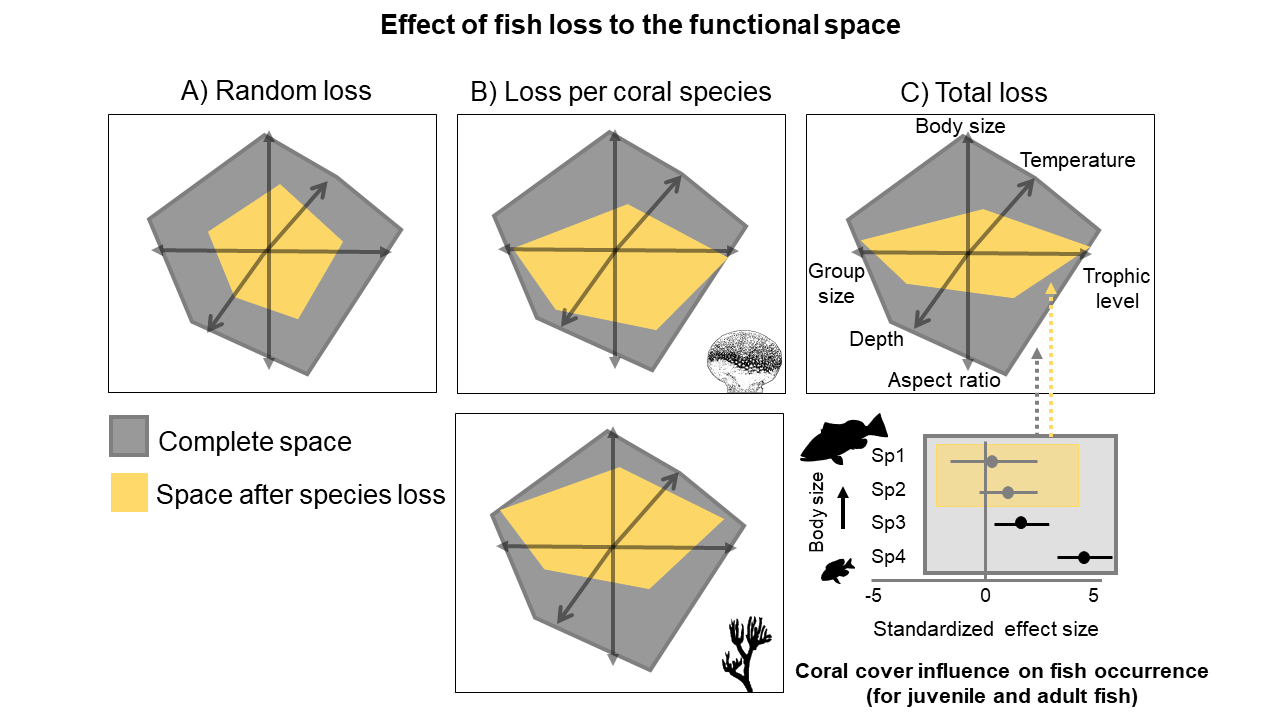


**Figure S1.1: Framework used to analyze the influence of coral and turf algal cover on the occurrence of juvenile and adult fish, and estimate the implications of losing coral-associated fish to the functional trait space.**

We represented the effect of fish loss by comparing the complete functional trait space area (made up by all fish in the dataset) with the area left after the simulated loss of fish in different scenarios (A, B, C). The simulated loss involved the removal of coral-associated fish (i.e., fish presenting positive influence of coral cover (represented by the standard effect size) and a neutral or negative influence of turf algae (fish with black bars and points in the coefficient plot at the bottom-right position)). Zero depicts the zone of no coral and turf algal cover effect on fish occurrence probability (as show for species with gray bars and points). In this hypothetical coefficient plot (bottom-right plot), fishes used to build the complete and the reduced functional trait space are represented using gray and yellow boxes, respectively. The standardized effect size of coral cover on fish occurrence is shown in the x-axis, and the list of fish species ordered by their body size is shown in the y-axis. This figure was produced and edited in Microsoft PowerPoint (https://www.microsoft.com/en-us/microsoft-365/powerpoint).

Table S1.1: Studied sites. We show geographic coordinates of the sites, and the cover of turf algae and corals, averaged across the plots deployed in each site. Sites used in site-occupancy modeling were the combination of locality, place and depth, as found in Aued et al. (2018) and Longo et al. (2019).

| Site | Longitude | Latitude | Turf algal cover | Coral cover | | | | | | | |
| --- | --- | --- | --- | --- | --- | --- | --- | --- | --- | --- | --- |
|  |  |  |  | *Agaricia* spp | *Favia gravida* | *Millepora alcicornis* | *Montastraea cavernosa* | *Mussismilia harttii* | *Mussismilia hispida* | *Porites astreoides* | *Siderastrea* spp |
| Abrolhos \|chapeirao \|shallow | -38.663 | -17.963 | 0.318 | 0.002 | 0.001 | 0.006 | 0.011 | 0.019 | 0.008 | 0.017 | 0.057 |
| Abrolhos \| portinho norte \| shallow | -38.698 | -17.961 | 0.694 | 0.000 | 0.006 | 0.004 | 0.001 | 0.000 | 0.001 | 0.000 | 0.019 |
| Abrolhos \| siriba \| shallow | -38.716 | -17.971 | 0.639 | 0.000 | 0.000 | 0.004 | 0.004 | 0.002 | 0.002 | 0.000 | 0.009 |
| Arraial \| anequim \| deep | -41.984 | -22.980 | 0.575 | 0.000 | 0.000 | 0.023 | 0.000 | 0.000 | 0.001 | 0.000 | 0.029 |
| Arraial \| anequim \| shallow | -41.984 | -22.980 | 0.668 | 0.000 | 0.000 | 0.013 | 0.000 | 0.000 | 0.000 | 0.000 | 0.000 |
| Arraial \| cardeiros \| deep | -42.002 | -22.965 | 0.440 | 0.000 | 0.000 | 0.015 | 0.000 | 0.000 | 0.002 | 0.001 | 0.008 |
| Arraial \| porcos oeste \| deep | -41.994 | -22.966 | 0.507 | 0.000 | 0.000 | 0.037 | 0.000 | 0.000 | 0.018 | 0.000 | 0.029 |
| Arraial \| porcos oeste \| shallow | -41.994 | -22.966 | 0.519 | 0.000 | 0.000 | 0.103 | 0.000 | 0.000 | 0.044 | 0.000 | 0.006 |
| Costa dos Corais \| barra da gale \| shallow | -35.193 | -9.033 | 0.653 | 0.001 | 0.000 | 0.058 | 0.000 | 0.034 | 0.007 | 0.001 | 0.001 |
| Costa dos Corais \| gales \| shallow | -35.191 | -9.024 | 0.591 | 0.000 | 0.000 | 0.034 | 0.000 | 0.030 | 0.001 | 0.000 | 0.001 |
| Costa dos Corais \| taocas \| shallow | -35.181 | -8.999 | 0.766 | 0.000 | 0.000 | 0.013 | 0.000 | 0.007 | 0.000 | 0.000 | 0.002 |
| Espirito Santo \| escalvada \| deep | -40.408 | -20.700 | 0.596 | 0.000 | 0.000 | 0.000 | 0.000 | 0.000 | 0.000 | 0.000 | 0.002 |
| Espirito Santo \| ilhas rasas \| deep | -40.366 | -20.677 | 0.570 | 0.000 | 0.000 | 0.002 | 0.000 | 0.000 | 0.000 | 0.000 | 0.005 |
| Espirito Santo \| tres ilhas \| deep | -40.379 | -20.612 | 0.581 | 0.000 | 0.000 | 0.000 | 0.000 | 0.000 | 0.000 | 0.000 | 0.000 |
| Espirito Santo \| tres ilhas \| shallow | -40.379 | -20.612 | 0.420 | 0.000 | 0.000 | 0.000 | 0.000 | 0.000 | 0.000 | 0.000 | 0.000 |
| Ilhabela \| ilha das cabras \| deep | -45.394 | -23.830 | 0.778 | 0.000 | 0.000 | 0.000 | 0.000 | 0.000 | 0.001 | 0.000 | 0.000 |
| Ilhabela \| ilha das cabras \| shallow | -45.394 | -23.830 | 0.808 | 0.000 | 0.000 | 0.000 | 0.000 | 0.000 | 0.000 | 0.000 | 0.000 |
| Ilhabela \| saco do diogo \| deep | -45.284 | -23.935 | 0.569 | 0.000 | 0.000 | 0.000 | 0.000 | 0.000 | 0.027 | 0.000 | 0.000 |
| Ilhabela \| saco do sombrio \| deep | -45.244 | -23.893 | 0.779 | 0.000 | 0.000 | 0.000 | 0.000 | 0.000 | 0.014 | 0.000 | 0.000 |
| Ilhabela \| saco do sombrio \| shallow | -45.244 | -23.893 | 0.768 | 0.000 | 0.000 | 0.000 | 0.000 | 0.000 | 0.000 | 0.000 | 0.000 |
| Ilha SC North \| deserta norte \| deep | -48.332 | -27.264 | 0.691 | 0.000 | 0.000 | 0.000 | 0.000 | 0.000 | 0.000 | 0.000 | 0.000 |
| Ilha SC North \| deserta norte \| shallow | -48.332 | -27.264 | 0.788 | 0.000 | 0.000 | 0.000 | 0.000 | 0.000 | 0.000 | 0.000 | 0.000 |
| Ilha SC North \| saco dagua \| deep | -48.367 | -27.274 | 0.643 | 0.000 | 0.000 | 0.000 | 0.000 | 0.000 | 0.000 | 0.000 | 0.000 |
| Ilha SC South \| xavier ponta sul \| deep | -48.388 | -27.604 | 0.686 | 0.000 | 0.000 | 0.000 | 0.000 | 0.000 | 0.000 | 0.000 | 0.000 |
| Ilha SC South \| xavier ponta sul \| shallow | -48.388 | -27.604 | 0.673 | 0.000 | 0.000 | 0.000 | 0.000 | 0.000 | 0.000 | 0.000 | 0.000 |
| Manuel Luis \| ana cristina \| deep | -44.264 | -0.870 | 0.184 | 0.000 | 0.000 | 0.000 | 0.000 | 0.000 | 0.000 | 0.000 | 0.005 |
| RGNorte Natal \| batente das agulhas \| deep | -35.073 | -5.564 | 0.371 | 0.000 | 0.000 | 0.000 | 0.000 | 0.000 | 0.000 | 0.000 | 0.000 |
| RGNorte Natal \| pedra do silva \| deep | -35.090 | -5.564 | 0.502 | 0.000 | 0.000 | 0.000 | 0.000 | 0.000 | 0.000 | 0.000 | 0.001 |
| RGNor Parrachos \| maracajau \| shallow | -35.259 | -5.394 | 0.521 | 0.000 | 0.001 | 0.000 | 0.000 | 0.000 | 0.000 | 0.003 | 0.070 |
| RGNor Parrachos \| parrachos de rio do fogo \| shallow | -35.363 | -5.262 | 0.152 | 0.000 | 0.001 | 0.000 | 0.000 | 0.000 | 0.000 | 0.000 | 0.002 |
| Rocas \| ancoras \| shallow | -33.804 | -3.875 | 0.752 | 0.000 | 0.000 | 0.000 | 0.000 | 0.000 | 0.001 | 0.004 | 0.017 |
| Rocas \| falsa barreta \| shallow | -33.819 | -3.860 | 0.591 | 0.000 | 0.000 | 0.000 | 0.000 | 0.000 | 0.000 | 0.000 | 0.043 |
| Rocas \| piscina das rocas \| shallow | -33.792 | -3.869 | 0.146 | 0.000 | 0.000 | 0.000 | 0.000 | 0.000 | 0.000 | 0.000 | 0.002 |
| Rocas \| podes crer \| shallow | -33.812 | -3.873 | 0.660 | 0.000 | 0.000 | 0.000 | 0.000 | 0.000 | 0.000 | 0.000 | 0.057 |
| Rocas \| salao \| deep | -33.809 | -3.875 | 0.301 | 0.000 | 0.000 | 0.000 | 0.000 | 0.000 | 0.000 | 0.000 | 0.192 |
| Rocas \| tartarugas \| shallow | -33.809 | -3.873 | 0.802 | 0.000 | 0.000 | 0.000 | 0.000 | 0.000 | 0.000 | 0.003 | 0.075 |

**Supplementary information file S2: Estimating the influence of coral and turf cover on fish occurrence using site-occupancy modeling**

We used site-occupancy modeling to estimate the strength of influence of coral and turf algal cover on fish occurrence probability, for fish in juvenile and adult life stages. Site-occupancy modeling allows to establish occupancy-environment relationships while assessing uncertainty in the process of observation of each species within the assemblage [1]. Such a hierarchical model has two sub models: ‘occupancy' and ‘observation’ models.

The ‘occupancy model’ deals with the biological processes (i.e., spatial variation on coral and turf cover) underlying the probability of site occupancy by the species. Occupancy of site *i* by the species *k* is denoted by a binary, partially observable variable z_ik_ that will be z_ik_ = 1 if site *i* is occupied by species *k*, and z_ik_ = 0 otherwise [2]. The probability of z_ik_ = 1 (i.e., the species *k* is truly present) is given by ψ_ik_, the site-occupancy probability, where

1.1 z_ik_ ~ Bernoulli (ψ_ik_)

[2]. In this case, z_ik_ is the realization of a Bernoulli process with success rate ψ_ik_. The parameter ψ_ik_ can be modeled in function of covariates through a generalized linear model (GLM) with a logit link function

1.2  *logit* (ψ_ik_) = β_0k_ + β_1k_ * X_coral[i]_ + β_2k_ * X_turf[i]_

[2]. Parameters β_0k_, β_1k_ and β_2k_ are the intercept and two regression coefficients of coral and turf influence on species *k*, respectively. The X_i_ depict covariates with values varying from *i* to I sites, being them, coral and turf algal cover. The regression coefficients are interpreted as standardized effect sizes of coral and turf cover on fish occupancy, in units of standard deviations from the intercept (average occupancy probability) produced by varying values of the covariates. Fish assemblage information was considered when modeling β_1k_. The *K* species were treated as random effects in the model, where

1.3 β_1k_ ~ Normal (µ_k_, σ^2^_k_)

[1]. Regression coefficients from *k* to K species were taken from a Normal distribution with average µ and variance σ^2^. Treating species as random effects means that assemblage species can show identical, but not any possible and independent, response to coral and turf cover [1].

The ‘observation model’ deals with the processes influencing species’ detection in truly occupied sites [1]. The observation model considers species detection (1) and non-detection (0) data across different sampling occasions (frames of 10 minutes of video plot recordings). Each video plot frame was considered as one sampling occasion for fish observation because fish sampling was not temporally replicated (as usually required in site-occupancy modeling, Mackenzie et al. [2]). Marine biologists use to maximize sampling effort over space as long travels and large expenses often challenge repeating marine surveys in the same sites over several days (e.g., Katsanevakis et al. [3]). The ‘time-for-space substitution’ strategy can then be applied to such spatially replicated data [1,4]. By doing so we assume that spatial samples are informative about the observation process (i.e., fishes will not be detected in some samples within a site), and there is no to negligible difference in occupancy probability across recording frames (i.e., any difference is caused by imperfect detection).

The history of observations *y* of the species *k* in site *i*, sampling occasion *j*, is conditional on the true site-occupancy state z_ik_, where

2.1 y_ijk_ | z_ik_ ~ Bernoulli (z_ik_p_ijk_)

[2]. An observation is a Bernoulli process with success rate depending on the product of z_ik_ and p_ijk_. This way, we will have detection in site *i*, sampling occasion *j*, if the site is truly occupied by the species *k*; if z_ik_ = 0 then y_ijk_ = 0; if z_ik_ = 1, then the species will be detected in a truly occupied site with probability p_ijk_. Like ψ_ik_, p_ijk_ also has its own GLM with logit link function

2.2  *logit* (p_ijk_) = α_0jk_ + α_1k_ * X_ij_

[2]. This model has a random-intercept where

2.3 α_0jk_ ~ Normal (µ_jk_, _σ_^2^_jk_)

The average probability of detecting the species *k* in the occasion *j*, in a truly occupied site, comes from a normal distribution (in the logit scale) with average µ and variance σ^2^. Such a random-intercept model was used to account for random spatial variation induced by deploying several video plots across space. We used this strategy as we lack data on the precise spatial location of transects and video plots (as required for modeling spatial autocorrelation in such a time-for-space substitution model [5]). Our inferences about fish response to coral cover, using spatially replicated data and replacing time for space, were valid because we found considerable variation in species detection probability across sampling occasions (range of 0.19 to 0.75 across plots, species, and models considering different coral species), indicating that the different video plots were informative about the process of fish observation.

The parameter α_1k_ in equation 2.2 is the regression coefficient of species *k*, and X_ij_ is a matrix of covariates with values varying from *i* to I sites, and *j* to *J* sampling occasions [2]. Thus, X_ij_ can have information on the sampling process across I sites and J occasions, or just across either I sites or J occasions. Here, we considered depth (a factor with the levels “1-7” and “8-15” m.), varying across I sites, could influence fish detection probability. Video observer was not considered to influence fish detection because all video recordings were either analyzed or supervised by the senior researcher of this study.

*Model goodness-of-fit, parameter convergence, and Monte-Carlo Markov Chain settings*

We used posterior predictive checks, based on the aggregation of detection data, to evaluate the fit of our models relative to the entry data (based on Kéry & Royle [6]). First, we aggregated detection data to obtain site-specific total number of detections for three data sets: 1) observed, 2) simulated under model structure, 3) expected given model structure. Second, we calculated a Chi-square discrepancy measure between observed vs. expected data, and between simulated vs. expected data. Then a Bayesian P-value was calculated based on the mentioned discrepancies, indicating whether a similar proportion of observed and simulated data deviates from expected number of detections [6]. A fitted and plausible model has a Bayesian P-value (BPV) close to 0.5 (i.e., observed and simulated data deviate equally relative to expected data). We assessed the convergence of estimated parameters using the Rhat value, which represents parameter variance across the independent MCMC. We considered an appropriate convergence at Rhat < 1.1.

Parameters were estimated using Bayesian inference, which involves the estimation of the posterior probability distribution and associated uncertainty of model parameters given prior knowledge on parameter distribution and the available data [1]. We used weak priors and hyperpriors in our models. We ran three parallel Monte-Carlo Markov Chains, with each chain consisting of 100,000 iterations, a burn-in phase of 50,000 iterations, and an adaptive phase of 30,000 iterations. We used a thinning of 50 iterations to extract samples of the posterior distribution of estimated parameters. These settings yielded 3,000 samples of the posterior distribution of each parameter. The codes were written in BUGS language, and models were implemented using the software JAGS [7], called from R environment [8] through the package “jagsUI” [9].

References

1. Kéry, M. & Royle, J.A. *Applied hierarchical modeling in ecology: analysis of distribution, abundance and species richness in R and BUGS. Vol. 1 Prelude and static models*. (eds. Kéry, M. & Royle, J.A.) 783 p. (Academic Press, London, UK, 2016)

2. MacKenzie, D.I. *et al.* Estimating site occupancy rates when detection probabilities are less than one. *Ecology* **83**, 2248–2255; 10.1890/0012-9658(2002)083[2248:ESORWD]2.0.CO;2; (2002)

3. Katsanevakis, S. *et al.* Monitoring marine populations and communities: methods dealing with imperfect detectability. *Aquat. Biol.* **16**, 31–52; 10.3354/ab00426 (2012)

4. Srivathsa, A., Puri, M., Kumar, N.S., Jathanna, D., & Karanth, K.U. Substituting space for time: Empirical evaluation of spatial replication as a surrogate for temporal replication in occupancy modelling. *J. Appl. Ecol.* **55**, 754-765; 10.1111/1365-2664.13005 (2018)

5. Kéry, M. & Royle, J.A. *Applied Hierarchical Modeling in Ecology: Analysis of Distribution, Abundance and Species Richness in R and BUGS: Volume 2: Dynamic and Advanced Models*. (eds. Kéry, M., Royle, J.A.) 820 p. (Academic Press, London, UK., 2020)

6. Hines, J.E. *et al.* Tigers on trails: occupancy modeling for cluster sampling. *Ecol. Appl.* **20**, 1456–1466; 10.1890/09-0321.1 (2010)

7. Plummer, M. JAGS: *A program for analysis of Bayesian graphical models using Gibbs sampling.* Proceedings of the 3rd international workshop on distributed statistical computing, 124, 1-10; (2003)

8. R Core Team. *R: A Language and Environment for Statistical Computing* (R Foundation for Statistical Computing, 2021)

9. Kellner, K. *jagsUI: A Wrapper Around 'rjags' to Streamline 'JAGS' Analyses.* R package version 1.5.1. <https://CRAN.R-project.org/package=jagsUI> (2019)


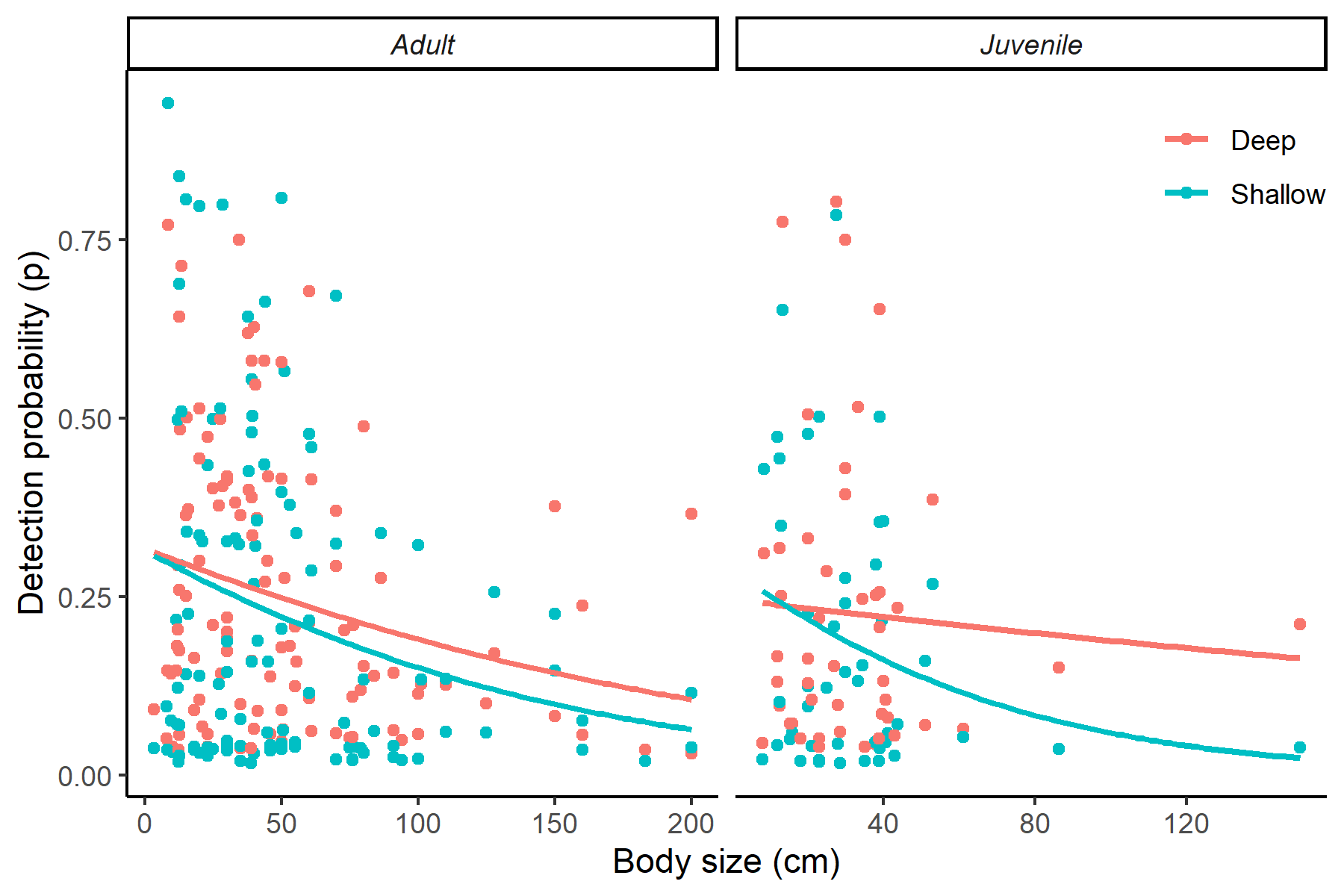


**Fig. S2.1. Detection probability of reef fish relative to maximum body size, sampling depth, and fish life stage.**

Detection probability *p* of reef fish was estimated for juvenile and adult fish across models with cover of turf algae and corals. The detection model had depth as a covariate (‘deep’ = 8-15 m, ‘shallow’ = 1-7m) and a random intercept. Points represent the average *p* across the 3,000 samples of the posterior distribution of the detection probability of each fish species in each coral model and age class. This figure was produced and edited in R v.4.1.2 (https://www.r-project.org/).

Table S2.1: Coefficients for all fishes, showing the influence of coral cover on fish occurrence probability. Bayesian P-value (BPV) of assemblage site-occupancy models using fish detection and non-detection data from video plots. A BPV close to 0.5 indicates good fit. BPV values for species with negative coefficients and too imprecise parameter estimates are not shown as they were not used to made inference on coral influence on fish occurrence. Average and credible intervals around estimates were obtained using 1,200 samples of the posterior probability distribution of each parameter. Age classes: 1- adult, 2-juvenile.

| Coral | Fish | Life stage class | intercept | low.int | high.int | estimate.coral | low.coral | high.coral | estimate.turf | low.turf | high.turf | Bayesian P-value |
| --- | --- | --- | --- | --- | --- | --- | --- | --- | --- | --- | --- | --- |
| *Agaricia spp* | *Acanthurus chirurgus* | 1 | 0.89 | 0.22 | 1.62 | -0.53 | -1.38 | 0.19 | -0.61 | -1.5 | 0.15 | 0 |
| *Agaricia spp* | *Chaetodon ocellatus* | 1 | -2.93 | -5.03 | -1.44 | -4.24 | -12.02 | -0.15 | -0.85 | -1.75 | -0.04 | 0.627 |
| *Agaricia spp* | *Haemulon plumierii* | 1 | 1.76 | -0.65 | 4.88 | 8.21 | -0.06 | 18.21 | -2.55 | -8.3 | -0.2 | 0.009 |
| *Agaricia spp* | *Halichoeres dimidiatus* | 1 | -1.44 | -4.07 | 1.41 | -6.19 | -22.33 | 5.25 | -3.18 | -14.04 | 0.02 | 0.089 |
| *Agaricia spp* | *Ocyurus chrysurus* | 1 | 0.13 | -1.13 | 1.66 | 6.24 | 1.44 | 12.69 | -0.18 | -0.95 | 0.61 | 0.003 |
| *Agaricia spp* | *Pomacanthus arcuatus* | 1 | -2.39 | -3.67 | -1.27 | -0.91 | -3.76 | 0.64 | 0.54 | -0.58 | 1.8 | 0.333 |
| *Agaricia spp* | *Pseudupeneus maculatus* | 1 | 2.1 | 0.71 | 4.56 | 1.22 | -1.45 | 8.61 | -1.08 | -2.93 | 0.17 | 0.01 |
| *Agaricia spp* | *Sparisoma axillare* | 1 | 2.56 | 1.16 | 4.68 | 4.59 | 0.16 | 12.66 | -0.86 | -1.94 | 0.03 | 0.002 |
| *Agaricia spp* | *Sparisoma frondosum* | 1 | 2.25 | 0.91 | 4.36 | 5.05 | 0.32 | 13.58 | 0.04 | -0.66 | 0.69 | 0.034 |
| *Agaricia spp* | *Thalassoma noronhanum* | 1 | -0.97 | -1.66 | -0.32 | -0.05 | -0.91 | 0.69 | -0.63 | -1.34 | 0.04 | 0.001 |
| *Agaricia spp* | *Abudefduf saxatilis* | 1 | 5.05 | 2.7 | 8.4 | 3.21 | -0.71 | 10.83 | -0.29 | -2.79 | 1.54 | 0 |
| *Agaricia spp* | *Acanthurus bahianus* | 1 | 2.41 | 1.04 | 4.64 | 5.01 | 0.34 | 13.89 | 0.17 | -0.52 | 0.85 | 0.009 |
| *Agaricia spp* | *Acanthurus coeruleus* | 1 | 1.78 | 0.3 | 3.86 | 6.63 | 1.07 | 15.14 | -0.97 | -1.88 | -0.18 | 0.065 |
| *Agaricia spp* | *Anisotremus virginicus* | 1 | 2.77 | 1.26 | 5.12 | 4.44 | 0.09 | 12.83 | -0.22 | -1.82 | 0.94 | 0.013 |
| *Agaricia spp* | *Bodianus rufus* | 1 | 0.76 | -0.13 | 2.35 | 0.6 | -1.12 | 5.72 | 0.49 | -0.29 | 1.29 | 0.008 |
| *Agaricia spp* | *Carangoides bartholomaei* | 1 | -0.81 | -2.22 | 0.48 | -4.16 | -10.23 | -0.37 | -0.29 | -1.18 | 0.56 | 0.022 |
| *Agaricia spp* | *Caranx ruber* | 1 | -1.02 | -4.6 | 2.5 | -9.61 | -54.97 | 17.97 | -5.16 | -23.04 | 0.48 | 0.364 |
| *Agaricia spp* | *Haemulon aurolineatum* | 1 | 1.29 | 0.38 | 2.54 | 2.77 | 0.17 | 7.28 | 0.22 | -0.43 | 0.84 | 0 |
| *Agaricia spp* | *Haemulon parra* | 1 | 0.65 | -1.02 | 3.16 | 5.8 | -0.26 | 16.42 | -0.87 | -1.89 | 0.03 | 0.479 |
| *Agaricia spp* | *Halichoeres bivittatus* | 1 | -0.32 | -3.43 | 2.61 | 12.71 | -24.4 | 47.27 | -32.07 | -66.58 | -4.87 | 0.286 |
| *Agaricia spp* | *Halichoeres brasiliensis* | 1 | 1.84 | 0.38 | 4.08 | 6.05 | 0.76 | 14.66 | 0.72 | 0.06 | 1.44 | 0 |
| *Agaricia spp* | *Halichoeres poeyi* | 1 | 1.34 | 0.47 | 2.58 | -0.2 | -1.41 | 1.93 | 0.35 | -0.55 | 1.28 | 0.008 |
| *Agaricia spp* | *Holacanthus ciliaris* | 1 | 0.65 | -1.08 | 2.99 | 5.06 | -0.14 | 13.38 | -1.18 | -3.54 | 0.06 | 0.077 |
| *Agaricia spp* | *Holocentrus adscensionis* | 1 | 1.4 | 0.58 | 2.37 | -0.35 | -1.35 | 0.4 | 0.21 | -0.61 | 1.05 | 0.001 |
| *Agaricia spp* | *Lutjanus jocu* | 1 | -0.14 | -1.38 | 1.77 | 2.92 | -0.24 | 10.84 | -0.63 | -1.54 | 0.16 | 0.06 |
| *Agaricia spp* | *Pomacanthus paru* | 1 | 0.35 | -0.39 | 1.41 | 0.72 | -0.49 | 4.05 | -0.02 | -0.73 | 0.66 | 0.005 |
| *Agaricia spp* | *Scarus trispinosus* | 1 | -0.61 | -1.99 | 1.65 | 3.08 | -0.17 | 12.64 | -1.26 | -4.28 | -0.02 | 0.005 |
| *Agaricia spp* | *Scarus zelindae* | 1 | 1.45 | -0.18 | 3.68 | 8.57 | 1.9 | 17.34 | -0.21 | -0.92 | 0.48 | 0.002 |
| *Agaricia spp* | *Sparisoma amplum* | 1 | 0.63 | -1.26 | 3.05 | 6.96 | 0.05 | 17.42 | 0.19 | -0.76 | 1.22 | 0.122 |
| *Agaricia spp* | *Sparisoma radians* | 1 | 1.67 | -0.25 | 4.34 | 6.18 | 0.22 | 15.68 | -1.01 | -3.1 | 0.64 | 0.737 |
| *Agaricia spp* | *Sphyraena barracuda* | 1 | -0.87 | -4.45 | 2.44 | 11.63 | -6.32 | 34.3 | -7.21 | -39.3 | -0.26 | 0.233 |
| *Agaricia spp* | *Stegastes variabilis* | 1 | 0.37 | -1.33 | 2.72 | 5.63 | 0.12 | 15.15 | 0.79 | -0.36 | 2.05 | 0.353 |
| *Agaricia spp* | *Chaetodon striatus* | 1 | 3.15 | 1.17 | 6 | 2.77 | -0.68 | 10.52 | 1.14 | -0.2 | 2.7 | 0 |
| *Agaricia spp* | *Lutjanus apodus* | 1 | -0.6 | -3.98 | 2.36 | 15.6 | -33.82 | 62.15 | -20.36 | -59.22 | -1.78 | 0.681 |
| *Agaricia spp* | *Stegastes fuscus* | 1 | 2.15 | 0.78 | 4.3 | 5.25 | 0.53 | 13.52 | 0.69 | 0 | 1.43 | 0.016 |
| *Agaricia spp* | *Acanthostracion polygonius* | 1 | -0.06 | -2.89 | 2.76 | -19.46 | -53.33 | 4.48 | -30.91 | -67.79 | 1.92 | 0.405 |
| *Agaricia spp* | *Amblycirrhitus pinos* | 1 | -0.74 | -4.35 | 2.42 | 9.01 | -25.18 | 42.28 | -21.66 | -59.18 | 1.02 | 0.484 |
| *Agaricia spp* | *Anisotremus surinamensis* | 1 | 0.16 | -2.74 | 3.21 | -32.93 | -75.48 | 0.35 | 3.5 | -5.89 | 13.72 | 0.342 |
| *Agaricia spp* | *Aulostomus maculatus* | 1 | -0.29 | -3.25 | 2.65 | 6.65 | -37.94 | 45.45 | -33.1 | -68.89 | -2.46 | 0.711 |
| *Agaricia spp* | *Cantherhines pullus* | 1 | -2.24 | -4.42 | -0.83 | -5.19 | -13.92 | -0.36 | -0.43 | -1.02 | 0.48 | 0.112 |
| *Agaricia spp* | *Canthidermis maculata* | 1 | -1.13 | -2.84 | 1.01 | 5.65 | 0.24 | 16.07 | 0.13 | -1.03 | 1.56 | 0.12 |
| *Agaricia spp* | *Cephalopholis fulva* | 1 | -1.3 | -2.04 | -0.57 | 0.14 | -0.64 | 0.89 | -0.19 | -0.93 | 0.59 | 0.202 |
| *Agaricia spp* | *Chromis multilineata* | 1 | 1.06 | -0.26 | 3.1 | 2.04 | -0.93 | 9.31 | 0.97 | -0.03 | 2.15 | 0.005 |
| *Agaricia spp* | *Clepticus brasiliensis* | 1 | -0.62 | -3.76 | 2.59 | -6.99 | -51.39 | 11.88 | -0.15 | -4.82 | 5.37 | 0.398 |
| *Agaricia spp* | *Epinephelus adscensionis* | 1 | -0.15 | -1.95 | 2.69 | 5.65 | -0.27 | 18.19 | 0.25 | -0.93 | 1.48 | 0.442 |
| *Agaricia spp* | *Haemulon squamipinna* | 1 | -0.23 | -2.69 | 2.22 | 21.76 | 5.48 | 46.76 | -37.35 | -69.25 | -11.49 | 0.075 |
| *Agaricia spp* | *Halichoeres penrosei* | 1 | -0.37 | -3.22 | 2.38 | -17 | -44.05 | 1.3 | -33.58 | -67.79 | -6.95 | 0.054 |
| *Agaricia spp* | *Microspathodon chrysurus* | 1 | -0.41 | -2.06 | 2.29 | 5.06 | -0.31 | 17.16 | -0.07 | -1.2 | 1.03 | 0.387 |
| *Agaricia spp* | *Ophioblennius macclurei* | 1 | -0.18 | -2.94 | 2.63 | 1.79 | -46.02 | 43.01 | -29.04 | -63.66 | 4.09 | 0.79 |
| *Agaricia spp* | *Scomberomorus regalis* | 1 | -0.25 | -3.33 | 2.64 | 0.16 | -47.13 | 41.9 | -27.89 | -66.61 | 8.07 | 0.791 |
| *Agaricia spp* | *Serranus baldwini* | 1 | -1.62 | -4.16 | 1.4 | -15.2 | -58.08 | -0.57 | 2.74 | -0.74 | 16.08 | 0.013 |
| *Agaricia spp* | *Halichoeres radiatus* | 1 | -2.05 | -4.27 | -0.63 | -5.79 | -14.98 | -0.61 | -0.29 | -1 | 0.38 | 0.001 |
| *Agaricia spp* | *Holacanthus tricolor* | 1 | -0.66 | -3.76 | 2.61 | -4.79 | -36.33 | 13.61 | -7.93 | -45 | 0.74 | 0.32 |
| *Agaricia spp* | *Lutjanus analis* | 1 | -0.38 | -3.5 | 2.53 | 10.77 | -38.4 | 58.74 | -25.41 | -61.86 | -2.69 | 0.717 |
| *Agaricia spp* | *Mycteroperca bonaci* | 1 | 0.29 | -1.61 | 2.79 | 7.63 | 0.53 | 17.2 | -0.01 | -1.62 | 1.51 | 0.314 |
| *Agaricia spp* | *Pseudocaranx dentex* | 1 | 0.03 | -2.85 | 2.96 | -25.41 | -61.97 | 2.33 | 18.14 | 1.81 | 43.49 | 0.483 |
| *Agaricia spp* | *Bothus ocellatus* | 1 | -0.56 | -3.99 | 2.56 | 13.46 | -40.75 | 57.78 | 17.42 | 0.27 | 54.46 | 0.513 |
| *Agaricia spp* | *Coryphopterus glaucofraenum* | 1 | -0.17 | -3.16 | 2.71 | -34.01 | -76.49 | -2.22 | -1.47 | -9.33 | 4.3 | 0.514 |
| *Agaricia spp* | *Malacanthus plumieri* | 1 | -2.79 | -4.86 | -1.36 | -4.54 | -12.57 | -0.12 | 0.37 | -0.43 | 1.34 | 0.7 |
| *Agaricia spp* | *Stegastes rocasensis* | 1 | -2.58 | -4.62 | -1.26 | -4.47 | -12.07 | -0.18 | -0.19 | -0.9 | 0.55 | 0.47 |
| *Agaricia spp* | *Apogon americanus* | 1 | -0.03 | -3.06 | 2.94 | -21.47 | -58.87 | 10.59 | -5.39 | -55.67 | 51.08 | 0.706 |
| *Agaricia spp* | *Aluterus scriptus* | 1 | -0.63 | -2.7 | 2.42 | 2.06 | -5.7 | 14.75 | 0.48 | -0.86 | 2.15 | 0.226 |
| *Agaricia spp* | *Melichthys niger* | 1 | -3.64 | -5.97 | -2.05 | -3.87 | -11.9 | 0.23 | -0.31 | -1.33 | 0.71 | 0.389 |
| *Agaricia spp* | *Rypticus saponaceus* | 1 | -0.2 | -3.18 | 2.65 | 18.24 | -2.29 | 49.91 | 30.15 | 3.63 | 64.83 | 0.766 |
| *Agaricia spp* | *Mulloidichthys martinicus* | 1 | -0.18 | -2.37 | 2.55 | 9.72 | 1.31 | 22.05 | 0.08 | -1.45 | 1.67 | 0.504 |
| *Agaricia spp* | *Caranx latus* | 1 | -1.06 | -4.04 | 1.97 | 9.9 | -0.91 | 32.78 | 18.61 | 1.03 | 63.91 | 0.407 |
| *Agaricia spp* | *Diodon hystrix* | 1 | -0.02 | -2.96 | 2.94 | -16.25 | -50.87 | 9.15 | -27 | -64.78 | 9.57 | 0.425 |
| *Agaricia spp* | *Cryptotomus roseus* | 1 | 1.13 | -0.82 | 3.58 | 8.38 | 0.12 | 18.5 | 0.13 | -0.64 | 0.94 | 0.001 |
| *Agaricia spp* | *Sparisoma tuiupiranga* | 1 | -0.61 | -1.45 | 0.83 | 1.6 | -0.17 | 7.49 | 0 | -0.7 | 0.72 | 0.103 |
| *Agaricia spp* | *Odontoscion dentex* | 1 | -2.13 | -5.14 | 1.45 | 0.78 | -18.02 | 34.65 | 13.48 | 0.73 | 49.28 | 0.367 |
| *Agaricia spp* | *Aluterus monoceros* | 1 | -0.67 | -3.76 | 2.29 | 42.45 | 14.24 | 78.59 | 2.47 | -4.84 | 11.65 | 0.333 |
| *Agaricia spp* | *Haemulon steindachneri* | 1 | -0.64 | -3.89 | 2.3 | 20.16 | -5.96 | 51.62 | 33.2 | 10.39 | 66.17 | 0.49 |
| *Agaricia spp* | *Lactophrys trigonus* | 1 | -0.63 | -3.59 | 2.13 | 41.89 | 12.56 | 80.93 | 2.96 | -5.28 | 13.81 | 0.331 |
| *Agaricia spp* | *Mycteroperca acutirostris* | 1 | -0.66 | -2.15 | 1.25 | -5.04 | -27.87 | 3.23 | 6.23 | 1.29 | 23.88 | 0.248 |
| *Agaricia spp* | *Synodus intermedius* | 1 | 0.16 | -2.18 | 2.62 | 47.38 | 19.47 | 82.25 | 2.48 | -5.07 | 11.35 | 0.433 |
| *Agaricia spp* | *Balistes vetula* | 1 | -0.25 | -3.63 | 2.92 | -30.32 | -66.21 | -1.41 | -10.47 | -36.92 | 20.56 | 0.312 |
| *Agaricia spp* | *Canthigaster figueiredoi* | 1 | -0.79 | -3.33 | 1.57 | -19.11 | -50.29 | -2.88 | 5.78 | 0.58 | 15.62 | 0 |
| *Agaricia spp* | *Malacoctenus delalandii* | 1 | -0.18 | -3.34 | 2.86 | -10.88 | -59.22 | 31.46 | 15.48 | -25.15 | 58.9 | 0.625 |
| *Agaricia spp* | *Anisotremus moricandi* | 1 | -1.01 | -4.85 | 2.46 | -1.87 | -39.01 | 23.26 | -5.96 | -55.8 | 26.73 | 0.423 |
| *Agaricia spp* | *Chilomycterus spinosus spinosus* | 1 | -0.17 | -3.15 | 2.9 | -28.74 | -68.98 | -1.12 | -8.51 | -27.47 | 10.7 | 0.153 |
| *Agaricia spp* | *Diplodus argenteus* | 1 | -1.85 | -4.04 | -0.34 | -6.96 | -15.4 | -1.18 | 1.39 | 0.53 | 2.42 | 0.282 |
| *Agaricia spp* | *Sphoeroides spengleri* | 1 | -1.14 | -3.51 | 1 | -13.05 | -28.04 | -2.7 | 4.43 | 1.01 | 9.57 | 0.094 |
| *Agaricia spp* | *Dactylopterus volitans* | 1 | -0.24 | -3.8 | 2.88 | -13.18 | -56.11 | 19.07 | 8.44 | -43.31 | 58.14 | 0.548 |
| *Agaricia spp* | *Kyphosus sectatrix* | 1 | 0.23 | -2.68 | 3.2 | -23.12 | -56.61 | 3.88 | -3.36 | -54.52 | 54.76 | 0.574 |
| *Agaricia spp* | *Aulostomus strigosus* | 1 | -0.18 | -3.52 | 2.87 | -15.91 | -53.22 | 13.53 | -8.93 | -65.58 | 45.12 | 0.62 |
| *Agaricia spp* | *Caranx crysos* | 1 | 0.11 | -2.92 | 3.03 | -20.53 | -55.8 | 3.28 | -15.95 | -64.37 | 42.02 | 0.555 |
| *Agaricia spp* | *Fistularia tabacaria* | 1 | 0.04 | -3 | 2.97 | -32.55 | -71.83 | -1.2 | 2.22 | -22.49 | 30.56 | 0.642 |
| *Agaricia spp* | *Chaetodon sedentarius* | 1 | -2.73 | -4.81 | -1.35 | -4.44 | -12.33 | -0.1 | 0 | -0.78 | 0.85 | 0.602 |
| *Agaricia spp* | *Priacanthus arenatus* | 1 | -1.69 | -5.42 | 2.24 | 3.53 | -15.93 | 24.72 | 0.37 | -5.27 | 5.46 | 0.46 |
| *Agaricia spp* | *Bodianus pulchellus* | 1 | -2.43 | -4.44 | -1.08 | -4.73 | -12.39 | -0.21 | 0.26 | -0.48 | 1.06 | 0.094 |
| *Agaricia spp* | *Elacatinus figaro* | 1 | -0.28 | -3.95 | 2.97 | -13.93 | -51.01 | 15.86 | -7.72 | -62.19 | 45.69 | 0.534 |
| *Agaricia spp* | *Sphoeroides greeleyi* | 1 | -0.41 | -4.2 | 2.83 | -13.54 | -51.79 | 17.02 | -6.46 | -56.12 | 47.75 | 0.521 |
| *Agaricia spp* | *Stephanolepis hispidus* | 1 | -2.25 | -4.37 | -0.88 | -5.37 | -13.68 | -0.38 | 0.28 | -0.4 | 1.03 | 0.015 |
| *Agaricia spp* | *Epinephelus morio* | 1 | -0.44 | -3.48 | 2.8 | -24.49 | -63.88 | 3.86 | 14.55 | 0.42 | 41.11 | 0.243 |
| *Agaricia spp* | *Heteropriacanthus cruentatus* | 1 | -0.31 | -3.86 | 2.7 | -8.89 | -51.04 | 29.44 | -27.88 | -69.19 | 8.27 | 0.475 |
| *Agaricia spp* | *Labrisomus nuchipinnis* | 1 | 0.01 | -3.14 | 3.05 | -29.88 | -70.17 | 2.1 | 6.56 | -17.66 | 32.93 | 0.473 |
| *Agaricia spp* | *Myrichthys ocellatus* | 1 | -0.34 | -3.93 | 2.67 | -4.56 | -44.45 | 32.44 | -28.71 | -66.69 | 0.73 | 0.462 |
| *Agaricia spp* | *Acanthostracion quadricornis* | 1 | -0.25 | -3.58 | 2.66 | 3.76 | -39.12 | 40.92 | -26.39 | -62.29 | 1.02 | 0.391 |
| *Agaricia spp* | *Epinephelus marginatus* | 1 | -2.48 | -5.07 | 0.06 | -5.92 | -17.21 | 2.73 | 8.36 | 1.27 | 25.7 | 0.099 |
| *Agaricia spp* | *Mugil curema* | 1 | -0.55 | -4.1 | 2.58 | 7.24 | -38.57 | 49.67 | -22.96 | -61.14 | 0.3 | 0.402 |
| *Agaricia spp* | *Orthopristis ruber* | 1 | 0.07 | -2.47 | 2.79 | 21.74 | -28.21 | 65.63 | 28.09 | 6.17 | 63.06 | 0.138 |
| *Agaricia spp* | *Kyphosus vaigiensis* | 1 | -2.16 | -6.44 | 2.22 | 9.19 | -19.29 | 52.05 | 8.86 | -0.22 | 39.81 | 0.134 |
| *Agaricia spp* | *Mycteroperca interstitialis* | 1 | -0.74 | -4.46 | 2.38 | 15.44 | -15.91 | 52.12 | 14.82 | -0.2 | 48.61 | 0.551 |
| *Agaricia spp* | *Eucinostomus melanopterus* | 1 | -0.74 | -4.5 | 2.34 | 25.3 | -12.15 | 65.34 | 13.41 | 1.1 | 45.87 | 0.569 |
| *Agaricia spp* | *Lutjanus cyanopterus* | 1 | -0.86 | -5.03 | 2.56 | 23.22 | -27.17 | 69.05 | 12.98 | 0.7 | 41.56 | 0.316 |
| *Agaricia spp* | *Calamus penna* | 1 | -0.2 | -3.46 | 2.77 | -16.92 | -55.8 | 13 | -14.1 | -63.17 | 42.86 | 0.615 |
| *Agaricia spp* | *Seriola rivoliana* | 1 | -0.11 | -3.55 | 2.84 | -13.58 | -48.99 | 14.87 | -18.66 | -63.56 | 29.87 | 0.601 |
| *Agaricia spp* | *Parablennius pilicornis* | 1 | -0.37 | -3.68 | 2.69 | -4.74 | -49.64 | 36.45 | 19.95 | -8.37 | 59.51 | 0.716 |
| *Agaricia spp* | *Pareques acuminatus* | 1 | -0.27 | -3.59 | 2.66 | -1.48 | -51.71 | 44.49 | 20.52 | -8.07 | 57.88 | 0.694 |
| *Agaricia spp* | *Rhomboplites aurorubens* | 1 | -1.39 | -5.5 | 2.13 | 11.94 | -6.47 | 35.2 | 2.48 | -0.48 | 6.91 | 0.565 |
| *Agaricia spp* | *Sphoeroides testudineus* | 1 | -0.01 | -3.05 | 3.03 | -0.71 | -50.81 | 42.2 | 24.32 | -14.93 | 61.88 | 0.791 |
| *Agaricia spp* | *Myripristis jacobus* | 1 | -0.79 | -4.09 | 2.13 | 40.47 | 11.23 | 76.64 | 0.6 | -7.94 | 10.59 | 0.53 |
| *Agaricia spp* | *Acanthurus bahianus* | 2 | -0.38 | -1.66 | 1.48 | 2.77 | -0.42 | 10.67 | 1 | -0.02 | 2.31 | 0.005 |
| *Agaricia spp* | *Acanthurus chirurgus* | 2 | -2.79 | -4.81 | -1.34 | -4.44 | -12.19 | -0.21 | -1.21 | -2.12 | -0.38 | 0.574 |
| *Agaricia spp* | *Sparisoma frondosum* | 2 | -0.62 | -2.37 | 1.82 | -4.44 | -16.45 | 0.63 | -2 | -6.67 | 0.01 | 0.451 |
| *Agaricia spp* | *Sparisoma axillare* | 2 | -0.18 | -3.38 | 2.9 | -29.1 | -66.93 | -0.45 | -17.93 | -41.96 | -0.61 | 0.34 |
| *Agaricia spp* | *Sparisoma radians* | 2 | 0.37 | -1.31 | 2.89 | 6.52 | -0.03 | 17.11 | -0.89 | -1.98 | -0.01 | 0.238 |
| *Agaricia spp* | *Stegastes variabilis* | 2 | 0.4 | -1.49 | 3.22 | 3.9 | -0.14 | 12.41 | 0.01 | -1.54 | 1.36 | 0.535 |
| *Agaricia spp* | *Abudefduf saxatilis* | 2 | 2.4 | 0.87 | 4.73 | 5.11 | 0.46 | 13.68 | 1.16 | 0.29 | 2.1 | 0.033 |
| *Agaricia spp* | *Acanthurus coeruleus* | 2 | -1.38 | -2.38 | -0.28 | 1.11 | -0.19 | 4.8 | -0.79 | -1.79 | 0.03 | 0.072 |
| *Agaricia spp* | *Chromis multilineata* | 2 | 0.93 | -0.84 | 3.71 | 3.55 | -0.49 | 11.88 | 0.21 | -1.06 | 1.43 | 0.031 |
| *Agaricia spp* | *Halichoeres brasiliensis* | 2 | -0.36 | -3.34 | 2.94 | 9.15 | -9.93 | 34.57 | -1.4 | -16.74 | 7.73 | 0.214 |
| *Agaricia spp* | *Halichoeres poeyi* | 2 | 0.03 | -0.85 | 2.03 | 1.4 | -0.66 | 9.96 | 0.26 | -0.38 | 0.92 | 0.007 |
| *Agaricia spp* | *Holacanthus tricolor* | 2 | -0.27 | -3.31 | 2.67 | 1.09 | -45.27 | 42.82 | -32.1 | -67.3 | -0.76 | 0.777 |
| *Agaricia spp* | *Microspathodon chrysurus* | 2 | -0.5 | -3.77 | 2.35 | 19.45 | -29.14 | 65.73 | -21.23 | -56.96 | -0.45 | 0.636 |
| *Agaricia spp* | *Anisotremus virginicus* | 2 | 0.08 | -3.05 | 3.11 | -33.2 | -73 | -1.01 | 5.47 | -18.67 | 32.15 | 0.395 |
| *Agaricia spp* | *Haemulon aurolineatum* | 2 | -0.4 | -2.25 | 2.38 | -9.85 | -45.45 | 6.97 | 10.1 | 0.74 | 36.15 | 0.221 |
| *Agaricia spp* | *Stegastes fuscus* | 2 | -0.19 | -1.51 | 2.42 | 3.13 | -0.4 | 14.13 | 1.1 | 0.15 | 2.26 | 0.14 |
| *Agaricia spp* | *Stegastes rocasensis* | 2 | -2.39 | -4.56 | -0.98 | -4.78 | -12.81 | -0.28 | -0.21 | -0.96 | 0.56 | 0.138 |
| *Agaricia spp* | *Thalassoma noronhanum* | 2 | -2.57 | -4.62 | -1.25 | -4.49 | -12.51 | -0.15 | -0.19 | -0.93 | 0.56 | 0.209 |
| *Agaricia spp* | *Halichoeres radiatus* | 2 | 0.12 | -1.71 | 3.14 | -2.97 | -10.6 | 0.71 | 0.02 | -1.6 | 1.81 | 0.244 |
| *Agaricia spp* | *Cantherhines pullus* | 2 | -1.21 | -4.78 | 2.46 | -18.44 | -59.12 | 6.04 | -0.34 | -19.42 | 19.62 | 0.26 |
| *Agaricia spp* | *Cryptotomus roseus* | 2 | -1.87 | -3 | -0.81 | -0.94 | -3.86 | 0.55 | 0.63 | -0.32 | 1.74 | 0.033 |
| *Agaricia spp* | *Haemulon plumierii* | 2 | -1.05 | -4.12 | 1.77 | 40.48 | 12.5 | 76.93 | 1.57 | -5.18 | 9.58 | 0.037 |
| *Agaricia spp* | *Canthigaster figueiredoi* | 2 | 0.7 | -1.76 | 3.62 | -9.07 | -53.45 | 18.47 | 9.74 | -0.82 | 34.28 | 0.414 |
| *Agaricia spp* | *Chaetodon striatus* | 2 | -0.34 | -3.24 | 2.51 | 13.63 | -23.27 | 49.85 | 19.69 | 0.14 | 57.52 | 0.269 |
| *Agaricia spp* | *Mycteroperca bonaci* | 2 | -0.73 | -3.72 | 2.01 | 40.9 | 13.9 | 77.44 | 2 | -5.51 | 11.18 | 0.318 |
| *Agaricia spp* | *Pomacanthus paru* | 2 | 0.03 | -2.65 | 3 | -18.23 | -48.56 | 4.96 | 30.89 | 4.17 | 64.98 | 0.313 |
| *Agaricia spp* | *Scarus zelindae* | 2 | 0.91 | -0.96 | 3.35 | 15.15 | 6.44 | 26.09 | 0.28 | -1.08 | 1.9 | 0.134 |
| *Agaricia spp* | *Sphoeroides spengleri* | 2 | -0.73 | -1.82 | 0.41 | -2.43 | -6.89 | 0.2 | 2.02 | 0.34 | 5.12 | 0.076 |
| *Agaricia spp* | *Ocyurus chrysurus* | 2 | -0.32 | -3.71 | 2.74 | -9.63 | -55.96 | 32.87 | 16.5 | -21.55 | 60.04 | 0.615 |
| *Agaricia spp* | *Labrisomus nuchipinnis* | 2 | -0.37 | -3.75 | 2.67 | 7.08 | -43.68 | 51.19 | 25.66 | -0.24 | 65.98 | 0.696 |
| *Agaricia spp* | *Bodianus pulchellus* | 2 | 0.01 | -2.86 | 2.97 | -17.05 | -52.57 | 8.87 | 25.12 | -0.64 | 60.3 | 0.64 |
| *Agaricia spp* | *Chilomycterus spinosus spinosus* | 2 | -0.3 | -3.9 | 2.71 | -12.09 | -48.09 | 16.64 | -7.49 | -51.19 | 39.01 | 0.525 |
| *Agaricia spp* | *Holocentrus adscensionis* | 2 | -0.11 | -3.16 | 2.85 | -19.36 | -53 | 4.07 | -28.96 | -67.26 | 3.8 | 0.317 |
| *Agaricia spp* | *Pseudupeneus maculatus* | 2 | -2.54 | -4.88 | 0.24 | -7.67 | -35.09 | 0.08 | 0.43 | -3.68 | 6.85 | 0.405 |
| *Agaricia spp* | *Serranus baldwini* | 2 | -1.53 | -4.2 | 1.64 | -16.92 | -65.04 | -0.5 | -0.29 | -15.85 | 13.32 | 0.384 |
| *Agaricia spp* | *Stephanolepis hispidus* | 2 | -3.02 | -5.21 | -1.61 | -4.27 | -12.46 | -0.02 | 0.01 | -0.83 | 0.92 | 0.432 |
| *Agaricia spp* | *Bodianus rufus* | 2 | -1.96 | -4.05 | 1.05 | 5.66 | -0.13 | 26.66 | 4.14 | -0.17 | 24.73 | 0.402 |
| *Agaricia spp* | *Diplodus argenteus* | 2 | -0.03 | -2.92 | 3.04 | -33.33 | -70.23 | -3.4 | 8.29 | -19.32 | 33.88 | 0.509 |
| *Agaricia spp* | *Halichoeres dimidiatus* | 2 | -1.3 | -4.83 | 2.21 | -4.49 | -38.69 | 28.33 | -23.93 | -67.23 | 0.73 | 0.168 |
| *Agaricia spp* | *Sparisoma tuiupiranga* | 2 | -0.45 | -3.67 | 2.52 | -4.9 | -44.39 | 35.1 | -32.84 | -71.68 | -0.3 | 0.188 |
| *Agaricia spp* | *Eucinostomus melanopterus* | 2 | -0.66 | -4.38 | 2.39 | 30.76 | -0.35 | 64.73 | 7.16 | 1.02 | 15.7 | 0.515 |
| *Agaricia spp* | *Sphoeroides greeleyi* | 2 | -0.32 | -3.51 | 2.65 | 6.25 | -45.95 | 52.73 | 23.6 | -0.85 | 61.96 | 0.701 |
| *Agaricia spp* | *Sphoeroides testudineus* | 2 | -0.29 | -3.32 | 2.66 | 6.81 | -45.26 | 50.17 | 25.39 | 0.51 | 63.24 | 0.695 |
| *Agaricia spp* | *Coryphopterus glaucofraenum* | 2 | -0.25 | -3.31 | 2.8 | 3.87 | -43.12 | 46.89 | 27.04 | 1.56 | 61.03 | 0.633 |
| *Agaricia spp* | *Synodus synodus* | 2 | -0.3 | -3.62 | 2.62 | -4.26 | -52.63 | 41.83 | 22.98 | -5.34 | 61.8 | 0.696 |
| *Agaricia spp* | *Pareques acuminatus* | 2 | -0.19 | -3.23 | 2.75 | -1.98 | -50.81 | 40.47 | 26.36 | -13.46 | 67.31 | 0.795 |
| *Agaricia spp* | *Sparisoma amplum* | 2 | -0.81 | -4.11 | 2.13 | 40.63 | 10.52 | 78.94 | 1.15 | -7.4 | 11.39 | 0.506 |
| *Favia gravida* | *Acanthurus chirurgus* | 1 | 1.07 | 0.32 | 1.94 | 1.52 | -0.11 | 4.44 | -0.37 | -1.16 | 0.39 | 0 |
| *Favia gravida* | *Chaetodon ocellatus* | 1 | -2.79 | -4.53 | -1.48 | -4.51 | -11.11 | -0.23 | -1.27 | -2.44 | -0.26 | 0.608 |
| *Favia gravida* | *Haemulon plumierii* | 1 | -0.07 | -1.09 | 1.55 | 3.03 | 0.4 | 7.56 | -0.68 | -2.47 | 0.41 | 0.049 |
| *Favia gravida* | *Halichoeres dimidiatus* | 1 | -2.41 | -5 | 0.1 | -7.76 | -19.8 | 0.2 | -1.84 | -6.67 | -0.15 | 0.1 |
| *Favia gravida* | *Ocyurus chrysurus* | 1 | -0.42 | -1.37 | 0.94 | 4.01 | 0.71 | 9.95 | 0.09 | -0.8 | 1.01 | 0.004 |
| *Favia gravida* | *Pomacanthus arcuatus* | 1 | -2.35 | -3.64 | -1.27 | 2.55 | 0.41 | 5.98 | 0.83 | -0.45 | 2.4 | 0.354 |
| *Favia gravida* | *Pseudupeneus maculatus* | 1 | 2.09 | 0.71 | 4.78 | 1.85 | -0.36 | 8.14 | -0.93 | -3.09 | 0.34 | 0.012 |
| *Favia gravida* | *Sparisoma axillare* | 1 | 2.12 | 0.97 | 4.13 | 2.5 | -0.25 | 10.36 | -0.89 | -2.02 | 0.08 | 0 |
| *Favia gravida* | *Sparisoma frondosum* | 1 | 2.07 | 0.9 | 3.74 | 4.89 | 0.55 | 11.82 | 0.31 | -0.46 | 1.06 | 0.038 |
| *Favia gravida* | *Thalassoma noronhanum* | 1 | -2.07 | -3.66 | -0.91 | -6.04 | -12.84 | -1.28 | -1.11 | -2.05 | -0.31 | 0 |
| *Favia gravida* | *Abudefduf saxatilis* | 1 | 4.96 | 2.7 | 8.15 | 3.85 | -0.5 | 11.79 | 0.07 | -2.36 | 1.86 | 0 |
| *Favia gravida* | *Acanthurus bahianus* | 1 | 1.39 | 0.71 | 2.16 | 0.5 | -0.52 | 2.2 | 0.18 | -0.51 | 0.9 | 0.009 |
| *Favia gravida* | *Acanthurus coeruleus* | 1 | 0.87 | -0.07 | 2.48 | 2.51 | 0.02 | 8.75 | -0.91 | -1.88 | -0.1 | 0.05 |
| *Favia gravida* | *Anisotremus virginicus* | 1 | 2.6 | 1.21 | 4.85 | 3.66 | 0.04 | 10.19 | -0.12 | -1.91 | 1.28 | 0.01 |
| *Favia gravida* | *Bodianus rufus* | 1 | -0.94 | -2.83 | 0.52 | -8.9 | -17.76 | -2.28 | 0.04 | -0.97 | 1.13 | 0.011 |
| *Favia gravida* | *Carangoides bartholomaei* | 1 | -1.31 | -3.08 | 0.1 | -8.99 | -20.23 | -1.83 | -1.22 | -3.12 | 0.07 | 0.035 |
| *Favia gravida* | *Caranx ruber* | 1 | -1.78 | -5.45 | 2.06 | -8.09 | -51.01 | 16.5 | -3.47 | -17.51 | 0.4 | 0.373 |
| *Favia gravida* | *Haemulon aurolineatum* | 1 | 1.49 | 0.47 | 2.74 | 4.1 | 0.48 | 9.34 | 0.46 | -0.29 | 1.23 | 0 |
| *Favia gravida* | *Haemulon parra* | 1 | -0.04 | -1.51 | 2.47 | -5.57 | -26.62 | 3.58 | -2.12 | -8.02 | 0.04 | 0.511 |
| *Favia gravida* | *Halichoeres bivittatus* | 1 | -0.2 | -3.31 | 2.64 | 14.85 | -23.12 | 49.02 | -32.2 | -67.78 | -5.37 | 0.293 |
| *Favia gravida* | *Halichoeres brasiliensis* | 1 | 0.66 | 0.03 | 1.33 | 0.83 | -0.3 | 2.65 | 0.7 | 0.06 | 1.43 | 0.001 |
| *Favia gravida* | *Halichoeres poeyi* | 1 | 1.33 | 0.5 | 2.38 | 0.73 | -0.45 | 2.71 | 0.45 | -0.34 | 1.32 | 0.01 |
| *Favia gravida* | *Holacanthus ciliaris* | 1 | 0.98 | -0.94 | 3.66 | 6.66 | 0.5 | 17.82 | -1.97 | -4.84 | -0.19 | 0.064 |
| *Favia gravida* | *Holocentrus adscensionis* | 1 | 1.38 | 0.63 | 2.29 | 0.45 | -0.54 | 1.96 | 0.32 | -0.45 | 1.14 | 0 |
| *Favia gravida* | *Lutjanus jocu* | 1 | -0.19 | -1.4 | 1.88 | 3.14 | 0.22 | 11.45 | -0.72 | -1.98 | 0.18 | 0.057 |
| *Favia gravida* | *Pomacanthus paru* | 1 | 0.3 | -0.35 | 1.02 | 0.89 | -0.19 | 2.73 | 0 | -0.68 | 0.64 | 0.004 |
| *Favia gravida* | *Scarus trispinosus* | 1 | -0.1 | -1.9 | 2.52 | 6.75 | 0.86 | 18.31 | -3.19 | -9.18 | -0.07 | 0.003 |
| *Favia gravida* | *Scarus zelindae* | 1 | -0.13 | -0.82 | 0.66 | 1.19 | -0.09 | 3.31 | -0.18 | -0.93 | 0.53 | 0.001 |
| *Favia gravida* | *Sparisoma amplum* | 1 | -1.41 | -4.04 | 1.7 | -15.51 | -54.45 | -1.08 | -0.52 | -5.74 | 2.38 | 0.165 |
| *Favia gravida* | *Sparisoma radians* | 1 | 0.21 | -2.56 | 3.18 | -35.81 | -76.33 | -5.35 | -2.47 | -11.23 | 4.31 | 0.714 |
| *Favia gravida* | *Sphyraena barracuda* | 1 | -1.6 | -5.65 | 2.11 | 9.09 | -10.91 | 35.08 | -6.52 | -38.4 | -0.42 | 0.208 |
| *Favia gravida* | *Stegastes variabilis* | 1 | -1.06 | -3.61 | 2 | -13.92 | -58.7 | 2.98 | 0.1 | -5.29 | 2.94 | 0.261 |
| *Favia gravida* | *Chaetodon striatus* | 1 | 3.58 | 1.34 | 7.08 | 4.75 | 0.01 | 12.13 | 1.72 | 0.17 | 4.08 | 0 |
| *Favia gravida* | *Lutjanus apodus* | 1 | -0.5 | -3.41 | 2.29 | 40.85 | 7.25 | 79.85 | -0.52 | -18.01 | 10.01 | 0.589 |
| *Favia gravida* | *Stegastes fuscus* | 1 | 1.86 | 0.83 | 3.21 | 4.65 | 0.82 | 10.08 | 1.03 | 0.25 | 1.87 | 0.018 |
| *Favia gravida* | *Acanthostracion polygonius* | 1 | -0.01 | -3.03 | 2.96 | -15.87 | -50.94 | 9.22 | -30.55 | -69.04 | -1.03 | 0.394 |
| *Favia gravida* | *Amblycirrhitus pinos* | 1 | -1.02 | -4.81 | 2.49 | 12.04 | -14.25 | 45.65 | -21.23 | -60.72 | 0.92 | 0.451 |
| *Favia gravida* | *Anisotremus surinamensis* | 1 | 0.18 | -2.78 | 3.26 | -30.75 | -72.68 | 1.35 | 2.56 | -6.03 | 11.6 | 0.34 |
| *Favia gravida* | *Aulostomus maculatus* | 1 | -0.26 | -3.33 | 2.53 | 7.16 | -35.64 | 46.07 | -30.35 | -65.41 | -0.86 | 0.727 |
| *Favia gravida* | *Cantherhines pullus* | 1 | -2.2 | -3.89 | -0.91 | -5.57 | -12.76 | -0.62 | -0.6 | -1.55 | 0.3 | 0.099 |
| *Favia gravida* | *Canthidermis maculata* | 1 | -1.78 | -2.95 | -0.71 | 3.28 | 0.66 | 7.34 | 0.17 | -0.92 | 1.46 | 0.218 |
| *Favia gravida* | *Cephalopholis fulva* | 1 | -1.98 | -3.45 | -0.89 | -4.39 | -10.83 | -0.33 | -0.63 | -1.65 | 0.27 | 0.182 |
| *Favia gravida* | *Chromis multilineata* | 1 | -0.75 | -2.61 | 0.89 | -7.32 | -16.54 | -0.85 | 0.95 | -0.29 | 2.5 | 0.005 |
| *Favia gravida* | *Clepticus brasiliensis* | 1 | -1.08 | -4.08 | 2.04 | -4.64 | -25.25 | 10.76 | -0.14 | -3.26 | 2.5 | 0.4 |
| *Favia gravida* | *Epinephelus adscensionis* | 1 | -1.16 | -2.34 | 0 | -0.55 | -4.16 | 4.52 | 0.35 | -0.86 | 1.88 | 0.621 |
| *Favia gravida* | *Haemulon squamipinna* | 1 | -1.58 | -2.97 | 0.64 | -0.09 | -4.03 | 6.69 | -0.9 | -4.54 | 0.57 | 0.361 |
| *Favia gravida* | *Halichoeres penrosei* | 1 | -0.59 | -3.96 | 2.45 | -14.4 | -42.34 | 4.77 | -30.19 | -68.66 | -1.2 | 0.076 |
| *Favia gravida* | *Microspathodon chrysurus* | 1 | -2.52 | -5.07 | -0.82 | -8.12 | -19.49 | -1.02 | -0.63 | -2.21 | 0.68 | 0.65 |
| *Favia gravida* | *Ophioblennius macclurei* | 1 | -0.19 | -3.16 | 2.71 | 3.9 | -42.76 | 44.88 | -30.53 | -68.42 | 2.37 | 0.803 |
| *Favia gravida* | *Scomberomorus regalis* | 1 | -0.17 | -3.23 | 2.7 | 2.06 | -41.85 | 44.08 | -28.67 | -65.19 | -0.03 | 0.79 |
| *Favia gravida* | *Serranus baldwini* | 1 | -2.34 | -4.68 | -0.79 | -8.03 | -20.71 | -0.81 | -0.03 | -1.3 | 0.88 | 0.018 |
| *Favia gravida* | *Halichoeres radiatus* | 1 | -1.68 | -3.12 | -0.59 | -5.27 | -11.53 | -0.83 | -0.69 | -1.62 | 0.13 | 0.002 |
| *Favia gravida* | *Holacanthus tricolor* | 1 | 0.06 | -3.08 | 3.57 | 3.43 | -19.9 | 20.9 | -2.85 | -23.4 | 0.89 | 0.391 |
| *Favia gravida* | *Lutjanus analis* | 1 | -0.57 | -3.77 | 2.4 | 16.39 | -31.67 | 63.94 | -24.11 | -58.29 | -2.59 | 0.725 |
| *Favia gravida* | *Mycteroperca bonaci* | 1 | -0.17 | -1.98 | 2.52 | 5.58 | 0.55 | 16.42 | -0.49 | -3.21 | 1.26 | 0.414 |
| *Favia gravida* | *Pseudocaranx dentex* | 1 | 0.75 | -1.22 | 3.34 | 11.2 | 1.85 | 22.97 | 9.31 | 2.29 | 21.67 | 0.562 |
| *Favia gravida* | *Bothus ocellatus* | 1 | -0.85 | -4.6 | 2.29 | 14.58 | -34.32 | 58.66 | 16.49 | 0.26 | 49.5 | 0.544 |
| *Favia gravida* | *Coryphopterus glaucofraenum* | 1 | 0.31 | -2.34 | 3.28 | -32.77 | -74.54 | -0.86 | -3.21 | -10.41 | 0.84 | 0.507 |
| *Favia gravida* | *Malacanthus plumieri* | 1 | -2.81 | -5.03 | -1.38 | -5.27 | -14.17 | -0.25 | 0.19 | -0.72 | 1.19 | 0.676 |
| *Favia gravida* | *Stegastes rocasensis* | 1 | -2.22 | -3.62 | -1.19 | -3.3 | -8.64 | -0.02 | -0.39 | -1.2 | 0.4 | 0.441 |
| *Favia gravida* | *Apogon americanus* | 1 | -0.16 | -3.37 | 2.76 | -19.95 | -58.71 | 12.49 | 1.12 | -48.6 | 56.81 | 0.67 |
| *Favia gravida* | *Aluterus scriptus* | 1 | -1.47 | -2.82 | 0.36 | 2.72 | 0.29 | 8.25 | 0.54 | -0.66 | 1.87 | 0.283 |
| *Favia gravida* | *Melichthys niger* | 1 | -3.76 | -5.94 | -2.13 | -4.81 | -13.44 | 0.11 | -0.56 | -1.68 | 0.55 | 0.391 |
| *Favia gravida* | *Rypticus saponaceus* | 1 | -0.18 | -3.2 | 2.64 | 0.21 | -51.83 | 44.71 | 29.48 | 2.07 | 66.97 | 0.816 |
| *Favia gravida* | *Mulloidichthys martinicus* | 1 | -1.15 | -4.09 | 2.13 | -12.71 | -59.41 | 10.22 | -0.1 | -5.32 | 4.18 | 0.403 |
| *Favia gravida* | *Caranx latus* | 1 | -0.91 | -4.11 | 2.22 | -0.62 | -42.47 | 36.94 | 26.08 | 1.02 | 65.78 | 0.474 |
| *Favia gravida* | *Diodon hystrix* | 1 | -0.02 | -3.02 | 2.89 | -14.83 | -51.44 | 11.43 | -29.72 | -68.79 | 0.9 | 0.437 |
| *Favia gravida* | *Cryptotomus roseus* | 1 | -0.6 | -1.29 | 0.09 | 1 | -0.1 | 2.69 | 0.33 | -0.39 | 1.09 | 0.001 |
| *Favia gravida* | *Sparisoma tuiupiranga* | 1 | -0.72 | -1.48 | 0.05 | 1.62 | 0.12 | 4.03 | 0.01 | -0.69 | 0.74 | 0.132 |
| *Favia gravida* | *Odontoscion dentex* | 1 | -1.67 | -4.74 | 1.51 | 9.74 | -11.27 | 45.87 | 17.41 | 0.68 | 54.17 | 0.316 |
| *Favia gravida* | *Aluterus monoceros* | 1 | -1.1 | -5.02 | 2.09 | 38.74 | 5.06 | 79.54 | 0.94 | -9.2 | 10.51 | 0.413 |
| *Favia gravida* | *Haemulon steindachneri* | 1 | -0.55 | -3.39 | 2.13 | 24.54 | 2.88 | 55.76 | 32.33 | 10.55 | 64.3 | 0.514 |
| *Favia gravida* | *Lactophrys trigonus* | 1 | -1.15 | -5.33 | 2.08 | 37.59 | 4.22 | 78.46 | 1.91 | -7.62 | 11.29 | 0.407 |
| *Favia gravida* | *Mycteroperca acutirostris* | 1 | -0.13 | -1.29 | 1.22 | 2.62 | -0.16 | 7.64 | 3.37 | 1.27 | 6.66 | 0.317 |
| *Favia gravida* | *Synodus intermedius* | 1 | -0.19 | -3.31 | 3.12 | 8.75 | -1.81 | 25.55 | 14 | -9.94 | 62.23 | 0.668 |
| *Favia gravida* | *Balistes vetula* | 1 | -1.01 | -4.54 | 2.83 | -23.58 | -65.61 | 0.17 | -3.12 | -31.24 | 26.02 | 0.3 |
| *Favia gravida* | *Canthigaster figueiredoi* | 1 | -0.06 | -2.43 | 2.45 | -16.66 | -48.76 | -1.25 | 5.65 | 0.21 | 14.88 | 0.002 |
| *Favia gravida* | *Malacoctenus delalandii* | 1 | -0.21 | -3.46 | 2.97 | -5.78 | -52.37 | 35.53 | 18.7 | -23.08 | 64.25 | 0.603 |
| *Favia gravida* | *Anisotremus moricandi* | 1 | -1.6 | -5.49 | 2.02 | 0.07 | -27.82 | 22.94 | -4.64 | -44.22 | 6.28 | 0.43 |
| *Favia gravida* | *Chilomycterus spinosus spinosus* | 1 | -0.76 | -4.01 | 2.66 | -22.27 | -58.68 | -0.54 | -4.98 | -20.18 | 8.82 | 0.219 |
| *Favia gravida* | *Diplodus argenteus* | 1 | -1.24 | -2.89 | -0.1 | -4.65 | -11.44 | -0.57 | 1.27 | 0.38 | 2.34 | 0.285 |
| *Favia gravida* | *Sphoeroides spengleri* | 1 | -0.29 | -2.46 | 1.88 | -9.86 | -25.43 | -0.51 | 4.21 | 0.62 | 9.8 | 0.088 |
| *Favia gravida* | *Dactylopterus volitans* | 1 | 0.64 | -2.51 | 3.77 | 14.89 | -32.98 | 48.06 | 10.92 | -11.19 | 55.82 | 0.576 |
| *Favia gravida* | *Kyphosus sectatrix* | 1 | 0.48 | -2.37 | 3.52 | -19.85 | -60.84 | 17.47 | 5.83 | -43.45 | 61.16 | 0.6 |
| *Favia gravida* | *Aulostomus strigosus* | 1 | -0.32 | -3.94 | 2.84 | -13.31 | -49.83 | 13.71 | -12.93 | -62.93 | 33 | 0.602 |
| *Favia gravida* | *Caranx crysos* | 1 | 0.09 | -2.86 | 3.03 | -20.3 | -56.23 | 3.74 | -10.15 | -62.93 | 53.52 | 0.546 |
| *Favia gravida* | *Fistularia tabacaria* | 1 | -0.07 | -3.39 | 3.27 | -30.08 | -69.04 | 0.44 | 3.74 | -18.24 | 31.18 | 0.617 |
| *Favia gravida* | *Chaetodon sedentarius* | 1 | -2.9 | -5.09 | -1.45 | -5.72 | -14.45 | -0.38 | -0.26 | -1.23 | 0.7 | 0.582 |
| *Favia gravida* | *Priacanthus arenatus* | 1 | -1.78 | -5.56 | 2.01 | 2.04 | -20.92 | 23.75 | 1.84 | -18.25 | 32.76 | 0.454 |
| *Favia gravida* | *Bodianus pulchellus* | 1 | -2.57 | -4.69 | -1.18 | -5.79 | -14.42 | -0.44 | 0.08 | -0.75 | 0.96 | 0.106 |
| *Favia gravida* | *Elacatinus figaro* | 1 | -0.4 | -4.28 | 2.87 | -12.14 | -50.79 | 16.39 | -1.92 | -54.6 | 51.24 | 0.551 |
| *Favia gravida* | *Sphoeroides greeleyi* | 1 | -0.27 | -3.85 | 2.8 | -14.47 | -51.69 | 13.71 | -11.32 | -64.55 | 44.58 | 0.535 |
| *Favia gravida* | *Stephanolepis hispidus* | 1 | -2.39 | -4.58 | -0.91 | -6.59 | -15.97 | -0.7 | 0.07 | -0.68 | 0.91 | 0.013 |
| *Favia gravida* | *Epinephelus morio* | 1 | -0.59 | -3.76 | 2.69 | -18.74 | -60.12 | 6.23 | 11.03 | 0 | 35.37 | 0.243 |
| *Favia gravida* | *Heteropriacanthus cruentatus* | 1 | -0.6 | -4.23 | 2.54 | -3.81 | -47.52 | 34.64 | -27.36 | -68.92 | 3.36 | 0.455 |
| *Favia gravida* | *Labrisomus nuchipinnis* | 1 | -0.1 | -3.51 | 3.2 | -28.07 | -68.16 | 3.47 | 6.07 | -14.47 | 32.88 | 0.444 |
| *Favia gravida* | *Myrichthys ocellatus* | 1 | -0.45 | -3.97 | 2.63 | -1.93 | -42.19 | 36.46 | -26.16 | -67.12 | 3.87 | 0.45 |
| *Favia gravida* | *Acanthostracion quadricornis* | 1 | -0.69 | -4.46 | 2.52 | 1.41 | -40.92 | 36.24 | -21.77 | -62.18 | 1.59 | 0.41 |
| *Favia gravida* | *Epinephelus marginatus* | 1 | -1.99 | -4.45 | 0.5 | -2.42 | -13.93 | 10.52 | 7.34 | 1.25 | 22.38 | 0.09 |
| *Favia gravida* | *Mugil curema* | 1 | -0.47 | -4.16 | 2.6 | 13.15 | -30.93 | 55.41 | -21.82 | -62.69 | 0.41 | 0.394 |
| *Favia gravida* | *Orthopristis ruber* | 1 | -0.07 | -2.93 | 2.9 | -10.38 | -50.94 | 17.03 | 28.75 | 2.79 | 65.42 | 0.161 |
| *Favia gravida* | *Kyphosus vaigiensis* | 1 | -2.36 | -6.45 | 1.8 | 5.97 | -31.37 | 50.06 | 9.44 | -0.49 | 42.23 | 0.13 |
| *Favia gravida* | *Mycteroperca interstitialis* | 1 | -0.85 | -4.44 | 2.21 | 16.18 | -15.03 | 52.19 | 15.56 | -0.17 | 47.25 | 0.512 |
| *Favia gravida* | *Eucinostomus melanopterus* | 1 | -0.81 | -4.45 | 2.24 | 28.4 | -5.45 | 67.36 | 11.63 | 1.25 | 33.12 | 0.586 |
| *Favia gravida* | *Lutjanus cyanopterus* | 1 | -1.51 | -5.71 | 1.96 | 21.93 | -20.26 | 65.57 | 11.98 | 0.65 | 40.27 | 0.322 |
| *Favia gravida* | *Calamus penna* | 1 | -0.27 | -3.76 | 3.02 | -12.45 | -49.77 | 15.45 | -14.58 | -62.12 | 31.44 | 0.599 |
| *Favia gravida* | *Seriola rivoliana* | 1 | -0.24 | -3.69 | 2.83 | -13.95 | -51.12 | 15.44 | -9.85 | -61.21 | 44.17 | 0.603 |
| *Favia gravida* | *Parablennius pilicornis* | 1 | -0.49 | -3.78 | 2.54 | 1.91 | -54.46 | 47.06 | 19.89 | -7.58 | 54.64 | 0.705 |
| *Favia gravida* | *Pareques acuminatus* | 1 | -0.31 | -3.58 | 2.81 | 0.14 | -51.59 | 46.96 | 21.92 | -3.77 | 59.37 | 0.691 |
| *Favia gravida* | *Rhomboplites aurorubens* | 1 | -1.5 | -5.43 | 1.91 | 11.81 | -6.88 | 34.02 | 2.31 | -0.54 | 6.38 | 0.551 |
| *Favia gravida* | *Sphoeroides testudineus* | 1 | -0.15 | -3.36 | 2.88 | -0.45 | -51.93 | 42.74 | 24.5 | -14.03 | 66.66 | 0.788 |
| *Favia gravida* | *Myripristis jacobus* | 1 | -0.28 | -3.49 | 2.73 | -2.02 | -52.79 | 41.53 | 21 | -13.38 | 61.04 | 0.764 |
| *Favia gravida* | *Acanthurus bahianus* | 2 | -0.76 | -1.64 | 0.15 | 1.72 | 0.07 | 4.46 | 1.13 | 0.14 | 2.38 | 0.006 |
| *Favia gravida* | *Acanthurus chirurgus* | 2 | -1.08 | -2.65 | 1.6 | 4.15 | -1.46 | 17.06 | -1.05 | -2.09 | -0.06 | 0.382 |
| *Favia gravida* | *Sparisoma frondosum* | 2 | -0.02 | -1.52 | 2.13 | 3.22 | -2.27 | 12.19 | -0.85 | -2.81 | 0.42 | 0.463 |
| *Favia gravida* | *Sparisoma axillare* | 2 | -0.84 | -3.83 | 2.43 | -19.08 | -63.27 | 10.44 | -11.19 | -36.36 | -0.56 | 0.405 |
| *Favia gravida* | *Sparisoma radians* | 2 | -1.15 | -2.18 | -0.13 | -1.54 | -5.24 | 1.77 | -0.81 | -1.95 | 0.08 | 0.406 |
| *Favia gravida* | *Stegastes variabilis* | 2 | 0.79 | -1.6 | 4.08 | -6.24 | -33.05 | 8.13 | -2.53 | -11.05 | 0.9 | 0.556 |
| *Favia gravida* | *Abudefduf saxatilis* | 2 | 3.22 | 1.03 | 6.78 | 8.98 | 0.65 | 23.69 | 1.98 | 0.72 | 3.53 | 0.02 |
| *Favia gravida* | *Acanthurus coeruleus* | 2 | -1.19 | -2.34 | 0.22 | 4.36 | 0.94 | 10.25 | -0.48 | -1.5 | 0.55 | 0.039 |
| *Favia gravida* | *Chromis multilineata* | 2 | 0.17 | -2.44 | 3 | -34.81 | -76.82 | -2.81 | -4.08 | -12.43 | 0.63 | 0.028 |
| *Favia gravida* | *Halichoeres brasiliensis* | 2 | -0.94 | -3.94 | 2.35 | 16.62 | 0.94 | 48.74 | -20.3 | -59.97 | 1.57 | 0.294 |
| *Favia gravida* | *Halichoeres poeyi* | 2 | -0.21 | -0.82 | 0.39 | 0.69 | -0.22 | 2.1 | 0.32 | -0.31 | 0.99 | 0.006 |
| *Favia gravida* | *Holacanthus tricolor* | 2 | -0.22 | -3.37 | 2.72 | 4.36 | -41.22 | 42.94 | -28.5 | -65.23 | 1.09 | 0.767 |
| *Favia gravida* | *Microspathodon chrysurus* | 2 | -0.68 | -4.36 | 2.27 | 20.52 | -21.63 | 63.7 | -18.49 | -50.31 | -0.53 | 0.63 |
| *Favia gravida* | *Anisotremus virginicus* | 2 | 0.2 | -2.17 | 3.15 | 7.87 | -1.66 | 20.25 | 0.75 | -3.56 | 4.96 | 0.394 |
| *Favia gravida* | *Haemulon aurolineatum* | 2 | -0.34 | -1.68 | 1.63 | 6.93 | 1.5 | 15.37 | 4.24 | 1.16 | 14.19 | 0.344 |
| *Favia gravida* | *Stegastes fuscus* | 2 | -0.47 | -1.28 | 0.36 | 3.14 | 0.4 | 6.85 | 1.62 | 0.42 | 2.88 | 0.199 |
| *Favia gravida* | *Stegastes rocasensis* | 2 | -1.99 | -3.39 | -0.88 | -4.08 | -10.4 | -0.16 | -0.53 | -1.51 | 0.38 | 0.118 |
| *Favia gravida* | *Thalassoma noronhanum* | 2 | -2.19 | -3.54 | -1.16 | -3.28 | -8.67 | -0.06 | -0.36 | -1.14 | 0.42 | 0.214 |
| *Favia gravida* | *Halichoeres radiatus* | 2 | -0.18 | -1.6 | 2.36 | 2.5 | 0.02 | 9.7 | -0.04 | -1.16 | 0.96 | 0.195 |
| *Favia gravida* | *Cantherhines pullus* | 2 | -1.47 | -5.04 | 2.34 | -17.13 | -61.5 | 5.73 | 1.54 | -14.76 | 21.69 | 0.275 |
| *Favia gravida* | *Cryptotomus roseus* | 2 | -1.7 | -2.69 | -0.77 | 2.04 | 0.25 | 5.03 | 0.85 | -0.24 | 2.1 | 0.032 |
| *Favia gravida* | *Haemulon plumierii* | 2 | -4.11 | -7.71 | -0.31 | 11.62 | 1.32 | 45.51 | 2.13 | -1.02 | 6.78 | 0.329 |
| *Favia gravida* | *Canthigaster figueiredoi* | 2 | -0.6 | -2.88 | 2.49 | 6.77 | 0.54 | 18.99 | 0.78 | -2.08 | 3.75 | 0.529 |
| *Favia gravida* | *Chaetodon striatus* | 2 | -1.33 | -4.27 | 1.8 | 19.36 | 1.13 | 56.72 | 13.47 | 0.21 | 44.2 | 0.355 |
| *Favia gravida* | *Mycteroperca bonaci* | 2 | -1.1 | -5.01 | 2.03 | 37.96 | 4.97 | 76.08 | 1.62 | -8.89 | 11.57 | 0.401 |
| *Favia gravida* | *Pomacanthus paru* | 2 | 0.23 | -2.38 | 3.47 | 4.04 | -3.47 | 15.11 | 16.84 | 0.11 | 56.47 | 0.325 |
| *Favia gravida* | *Scarus zelindae* | 2 | -1.27 | -2.41 | 0.33 | 2.73 | 0.28 | 8.09 | 0.68 | -0.43 | 1.97 | 0.17 |
| *Favia gravida* | *Sphoeroides spengleri* | 2 | -0.27 | -1.17 | 0.84 | 1.53 | -0.15 | 4.59 | 1.61 | 0.35 | 3.7 | 0.107 |
| *Favia gravida* | *Ocyurus chrysurus* | 2 | -0.4 | -3.92 | 2.8 | -5.22 | -52.43 | 35.24 | 16.05 | -21.27 | 60.34 | 0.604 |
| *Favia gravida* | *Labrisomus nuchipinnis* | 2 | -0.37 | -3.56 | 2.67 | 5.26 | -51.69 | 54.01 | 22.98 | -0.76 | 61.09 | 0.694 |
| *Favia gravida* | *Bodianus pulchellus* | 2 | 0.1 | -2.92 | 3.08 | -18.99 | -61.29 | 9.72 | 25.34 | -6.26 | 66.88 | 0.646 |
| *Favia gravida* | *Chilomycterus spinosus spinosus* | 2 | -0.4 | -4.31 | 2.76 | -12.66 | -51.06 | 15.97 | -8.39 | -58.79 | 42.05 | 0.534 |
| *Favia gravida* | *Holocentrus adscensionis* | 2 | -0.11 | -3.35 | 2.91 | -14.81 | -46.84 | 9.1 | -25.12 | -62.93 | 7.85 | 0.298 |
| *Favia gravida* | *Pseudupeneus maculatus* | 2 | -2.86 | -5.14 | -1.3 | -5.25 | -14.96 | 0.1 | 0.15 | -0.95 | 1.38 | 0.457 |
| *Favia gravida* | *Serranus baldwini* | 2 | -2.34 | -4.94 | 0.06 | -9.88 | -33.33 | -0.86 | -0.33 | -2.11 | 1.2 | 0.504 |
| *Favia gravida* | *Stephanolepis hispidus* | 2 | -3.13 | -5.41 | -1.64 | -5.28 | -14.36 | -0.17 | -0.19 | -1.1 | 0.75 | 0.418 |
| *Favia gravida* | *Bodianus rufus* | 2 | -2.77 | -5.22 | -0.27 | -3.43 | -15.06 | 9.08 | 3.79 | -0.76 | 29.89 | 0.51 |
| *Favia gravida* | *Diplodus argenteus* | 2 | 0.08 | -2.89 | 3.23 | -32.33 | -69.81 | -1.83 | 8.97 | -15.2 | 35.49 | 0.466 |
| *Favia gravida* | *Halichoeres dimidiatus* | 2 | -1.42 | -5.06 | 2.16 | -0.09 | -29.86 | 32.77 | -22.77 | -62.81 | 0.38 | 0.168 |
| *Favia gravida* | *Sparisoma tuiupiranga* | 2 | -0.24 | -3.42 | 2.75 | 1.92 | -43.6 | 39.45 | -32.15 | -70.25 | 0.71 | 0.193 |
| *Favia gravida* | *Eucinostomus melanopterus* | 2 | -1.24 | -5.03 | 1.87 | 28.33 | -2.5 | 65.91 | 6.8 | 0.84 | 14.7 | 0.489 |
| *Favia gravida* | *Sphoeroides greeleyi* | 2 | -0.41 | -3.69 | 2.48 | 13.07 | -33.08 | 57.41 | 23.25 | 0.5 | 58.32 | 0.692 |
| *Favia gravida* | *Sphoeroides testudineus* | 2 | -0.43 | -3.7 | 2.47 | 11.03 | -38.73 | 54.36 | 23.31 | 0.05 | 59.01 | 0.683 |
| *Favia gravida* | *Coryphopterus glaucofraenum* | 2 | -0.25 | -3.19 | 2.58 | 14.21 | -36.47 | 62.51 | 29.27 | 3.28 | 66.14 | 0.638 |
| *Favia gravida* | *Synodus synodus* | 2 | -0.38 | -3.79 | 2.68 | 2.8 | -48.07 | 50.49 | 23.81 | -1.82 | 63.52 | 0.702 |
| *Favia gravida* | *Pareques acuminatus* | 2 | -0.15 | -3.22 | 3.01 | 0.81 | -50.45 | 42.83 | 25.89 | -8.58 | 65.77 | 0.766 |
| *Favia gravida* | *Sparisoma amplum* | 2 | -0.17 | -3.31 | 2.89 | -3.16 | -50.79 | 40.86 | 21.09 | -13.85 | 59.95 | 0.772 |
| *Millepora alcicornis* | *Acanthurus chirurgus* | 1 | 0.96 | 0.29 | 1.71 | 0.69 | -0.26 | 2.02 | -0.45 | -1.25 | 0.22 | 0 |
| *Millepora alcicornis* | *Chaetodon ocellatus* | 1 | -3.1 | -5.47 | -1.51 | -3.38 | -8.84 | -0.06 | -0.74 | -1.6 | 0.05 | 0.615 |
| *Millepora alcicornis* | *Haemulon plumierii* | 1 | 0.24 | -0.92 | 1.92 | 2.8 | 0.08 | 6.47 | -1.03 | -2.82 | -0.04 | 0.037 |
| *Millepora alcicornis* | *Halichoeres dimidiatus* | 1 | 0.81 | -1.13 | 3.34 | 6.99 | 1.19 | 13.82 | -1.83 | -5.01 | 0 | 0.061 |
| *Millepora alcicornis* | *Ocyurus chrysurus* | 1 | -0.79 | -1.56 | 0.04 | 0.26 | -0.91 | 1.83 | -0.22 | -0.92 | 0.48 | 0.002 |
| *Millepora alcicornis* | *Pomacanthus arcuatus* | 1 | -2.45 | -3.87 | -1.16 | -1.94 | -4.97 | 0.45 | 1.48 | -0.54 | 2.15 | 0.305 |
| *Millepora alcicornis* | *Pseudupeneus maculatus* | 1 | 1.75 | 0.76 | 3.15 | 1.68 | -0.01 | 4.18 | -0.57 | -1.76 | 0.29 | 0.017 |
| *Millepora alcicornis* | *Sparisoma axillare* | 1 | 2.92 | 1.32 | 5.32 | 4.44 | 0.67 | 10.32 | -0.81 | -1.82 | 0.02 | 0 |
| *Millepora alcicornis* | *Sparisoma frondosum* | 1 | 2.28 | 0.81 | 4.55 | 3.38 | 0.04 | 9.15 | -0.01 | -0.7 | 0.68 | 0.024 |
| *Millepora alcicornis* | *Thalassoma noronhanum* | 1 | -1.03 | -1.74 | -0.39 | -0.43 | -1.52 | 0.44 | -0.61 | -1.33 | 0.07 | 0 |
| *Millepora alcicornis* | *Abudefduf saxatilis* | 1 | 4.97 | 2.58 | 8.34 | 1.67 | -0.96 | 6.46 | -0.3 | -2.79 | 1.46 | 0 |
| *Millepora alcicornis* | *Acanthurus bahianus* | 1 | 2.77 | 1.23 | 5.26 | 4.64 | 0.76 | 10.75 | 0.13 | -0.54 | 0.79 | 0.013 |
| *Millepora alcicornis* | *Acanthurus coeruleus* | 1 | 0.97 | -0.17 | 3.24 | 1.04 | -0.94 | 6.45 | -1.16 | -2.32 | -0.27 | 0.041 |
| *Millepora alcicornis* | *Anisotremus virginicus* | 1 | 2.38 | 1.12 | 4.4 | 2.05 | -0.15 | 5.84 | -0.16 | -1.56 | 0.97 | 0.005 |
| *Millepora alcicornis* | *Bodianus rufus* | 1 | 1.16 | 0.06 | 2.85 | 2.06 | -0.51 | 6.34 | 0.47 | -0.25 | 1.26 | 0.004 |
| *Millepora alcicornis* | *Carangoides bartholomaei* | 1 | -0.47 | -1.34 | 0.43 | -0.55 | -1.93 | 0.58 | -0.17 | -0.87 | 0.54 | 0.027 |
| *Millepora alcicornis* | *Caranx ruber* | 1 | -1.04 | -4.65 | 2.58 | -21.01 | -65.96 | 1.05 | -11.56 | -40.59 | 0.7 | 0.303 |
| *Millepora alcicornis* | *Haemulon aurolineatum* | 1 | 0.9 | 0.19 | 1.7 | 0.48 | -0.44 | 1.75 | 0.15 | -0.5 | 0.8 | 0 |
| *Millepora alcicornis* | *Haemulon parra* | 1 | 0.42 | -1.03 | 2.34 | 4.25 | 0.76 | 9.17 | -0.95 | -2 | -0.07 | 0.646 |
| *Millepora alcicornis* | *Halichoeres bivittatus* | 1 | -0.4 | -3.67 | 2.62 | 4.32 | -28.51 | 35.09 | -35.39 | -71.53 | -5.75 | 0.286 |
| *Millepora alcicornis* | *Halichoeres brasiliensis* | 1 | 2.44 | 0.76 | 4.85 | 6.38 | 2.08 | 12.38 | 0.74 | 0.04 | 1.52 | 0 |
| *Millepora alcicornis* | *Halichoeres poeyi* | 1 | 1.55 | 0.55 | 3.17 | 1.48 | -0.2 | 5.12 | 0.38 | -0.33 | 1.16 | 0.012 |
| *Millepora alcicornis* | *Holacanthus ciliaris* | 1 | -0.32 | -1.66 | 1.39 | -2.56 | -7.36 | 0.05 | -2.6 | -7.03 | -0.16 | 0.092 |
| *Millepora alcicornis* | *Holocentrus adscensionis* | 1 | 1.6 | 0.74 | 2.59 | 1.54 | -0.04 | 3.87 | 0.21 | -0.49 | 0.94 | 0 |
| *Millepora alcicornis* | *Lutjanus jocu* | 1 | -0.89 | -2.69 | 1.36 | -8.57 | -46.34 | -0.39 | -1.79 | -7.95 | 0.24 | 0.043 |
| *Millepora alcicornis* | *Pomacanthus paru* | 1 | 0.24 | -0.4 | 0.92 | 0.19 | -0.56 | 1.04 | -0.05 | -0.72 | 0.59 | 0.005 |
| *Millepora alcicornis* | *Scarus trispinosus* | 1 | -1.3 | -2.69 | 0.27 | -1.96 | -5.42 | 0.13 | -1.48 | -5.57 | -0.05 | 0.005 |
| *Millepora alcicornis* | *Scarus zelindae* | 1 | 0.78 | -0.5 | 2.55 | 4.82 | 1.6 | 9.43 | -0.32 | -1.04 | 0.39 | 0.002 |
| *Millepora alcicornis* | *Sparisoma amplum* | 1 | -0.36 | -1.52 | 1.17 | 1.99 | -0.12 | 5.33 | 0.23 | -0.66 | 1.21 | 0.207 |
| *Millepora alcicornis* | *Sparisoma radians* | 1 | 1.52 | -0.49 | 4.34 | 2.58 | -0.42 | 7.57 | -1.01 | -3.32 | 0.88 | 0.74 |
| *Millepora alcicornis* | *Sphyraena barracuda* | 1 | -2.43 | -5.49 | 1.27 | 1.72 | -8.31 | 24.03 | -11.98 | -56.03 | -0.04 | 0.131 |
| *Millepora alcicornis* | *Stegastes variabilis* | 1 | -0.25 | -1.65 | 1.99 | 2.1 | -0.67 | 7.48 | 1.4 | -0.3 | 2.22 | 0.34 |
| *Millepora alcicornis* | *Chaetodon striatus* | 1 | 3.02 | 1.17 | 5.84 | 2.38 | -0.59 | 7.97 | 1 | -0.11 | 2.38 | 0 |
| *Millepora alcicornis* | *Lutjanus apodus* | 1 | -0.32 | -3.82 | 2.73 | 9.92 | -34.93 | 54.7 | -26.14 | -64.33 | -2.43 | 0.728 |
| *Millepora alcicornis* | *Stegastes fuscus* | 1 | 2.67 | 1.07 | 5.18 | 5.34 | 1.32 | 11.42 | 0.65 | 0.01 | 1.34 | 0.024 |
| *Millepora alcicornis* | *Acanthostracion polygonius* | 1 | 0.14 | -2.52 | 2.86 | 15.22 | 0.73 | 38.94 | -36.63 | -71.71 | -7.17 | 0.324 |
| *Millepora alcicornis* | *Amblycirrhitus pinos* | 1 | -1.03 | -4.87 | 2.53 | 2.56 | -26.29 | 31.66 | -27.7 | -67.76 | 0.69 | 0.449 |
| *Millepora alcicornis* | *Anisotremus surinamensis* | 1 | 0.03 | -2.83 | 2.99 | -37.23 | -76.89 | -4.49 | 5.77 | -10.59 | 21.15 | 0.302 |
| *Millepora alcicornis* | *Aulostomus maculatus* | 1 | -0.16 | -3.16 | 2.8 | -2.99 | -43.9 | 30.56 | -32.04 | -69.94 | 0.12 | 0.746 |
| *Millepora alcicornis* | *Cantherhines pullus* | 1 | -1.23 | -2.06 | -0.47 | 1.29 | 0.3 | 2.46 | -0.2 | -0.96 | 0.59 | 0.111 |
| *Millepora alcicornis* | *Canthidermis maculata* | 1 | -2.02 | -3.16 | -1.01 | -1.02 | -3.25 | 0.49 | -0.2 | -1.1 | 0.69 | 0.232 |
| *Millepora alcicornis* | *Cephalopholis fulva* | 1 | -1.01 | -1.98 | 0.25 | 1.58 | -0.24 | 5.59 | -0.35 | -1.17 | 0.45 | 0.159 |
| *Millepora alcicornis* | *Chromis multilineata* | 1 | 0.84 | -0.28 | 2.42 | 1.86 | 0.16 | 5.08 | 1.04 | 0.07 | 2.28 | 0.004 |
| *Millepora alcicornis* | *Clepticus brasiliensis* | 1 | -0.46 | -3.35 | 2.62 | -23.46 | -68.85 | 0.7 | 2.87 | -9.75 | 18.73 | 0.356 |
| *Millepora alcicornis* | *Epinephelus adscensionis* | 1 | -0.18 | -1.75 | 1.65 | 5.87 | 1.59 | 11.71 | 0.05 | -1.14 | 1.41 | 0.65 |
| *Millepora alcicornis* | *Haemulon squamipinna* | 1 | 0.08 | -2.46 | 2.86 | 14.79 | 0.84 | 32.45 | -33.14 | -65.94 | -0.52 | 0.084 |
| *Millepora alcicornis* | *Halichoeres penrosei* | 1 | -0.41 | -3.36 | 2.32 | -15.32 | -37.5 | -1.29 | -38.24 | -74.97 | -9.39 | 0.031 |
| *Millepora alcicornis* | *Microspathodon chrysurus* | 1 | -0.22 | -1.74 | 1.64 | 6.15 | 1.66 | 12.46 | -0.38 | -1.57 | 0.79 | 0.585 |
| *Millepora alcicornis* | *Ophioblennius macclurei* | 1 | -0.15 | -3.14 | 2.73 | -3.73 | -42.67 | 31.8 | -29.3 | -70.84 | 9.83 | 0.802 |
| *Millepora alcicornis* | *Scomberomorus regalis* | 1 | -0.12 | -3.13 | 2.84 | -5.15 | -45.85 | 29.21 | -29.47 | -66.85 | 7.07 | 0.798 |
| *Millepora alcicornis* | *Serranus baldwini* | 1 | 0 | -1.76 | 2.51 | 4.62 | 0.48 | 12.04 | 0.92 | -0.45 | 3.18 | 0.004 |
| *Millepora alcicornis* | *Halichoeres radiatus* | 1 | -0.9 | -1.61 | -0.21 | -0.61 | -1.91 | 0.33 | -0.24 | -0.89 | 0.4 | 0.001 |
| *Millepora alcicornis* | *Holacanthus tricolor* | 1 | -0.05 | -2.24 | 3.05 | 3 | -1.62 | 10.47 | -2.05 | -9.23 | 0.93 | 0.379 |
| *Millepora alcicornis* | *Lutjanus analis* | 1 | -0.2 | -3.32 | 2.82 | 10.52 | -39.05 | 55.26 | -27.08 | -61.89 | -0.94 | 0.751 |
| *Millepora alcicornis* | *Mycteroperca bonaci* | 1 | 0.55 | -2.16 | 3.84 | -29.17 | -72.71 | -0.05 | -5.82 | -18.48 | 2.14 | 0.338 |
| *Millepora alcicornis* | *Pseudocaranx dentex* | 1 | -0.08 | -3.05 | 2.85 | -24.86 | -54.88 | -2.95 | 25.07 | 2.36 | 57.11 | 0.448 |
| *Millepora alcicornis* | *Bothus ocellatus* | 1 | -0.69 | -4.61 | 2.53 | 1.76 | -45.28 | 47.95 | 22.04 | -0.02 | 56.68 | 0.608 |
| *Millepora alcicornis* | *Coryphopterus glaucofraenum* | 1 | -0.2 | -2 | 2.86 | 1.69 | -0.34 | 6.26 | 0.51 | -0.86 | 1.64 | 0.535 |
| *Millepora alcicornis* | *Malacanthus plumieri* | 1 | -3.01 | -5.41 | -1.46 | -3.86 | -9.74 | -0.32 | 0.37 | -0.43 | 1.29 | 0.7 |
| *Millepora alcicornis* | *Stegastes rocasensis* | 1 | -2.87 | -5.16 | -1.38 | -4.01 | -9.63 | -0.47 | -0.16 | -0.83 | 0.57 | 0.447 |
| *Millepora alcicornis* | *Apogon americanus* | 1 | -0.01 | -3.06 | 2.99 | -21.43 | -52.96 | 0.88 | -10.45 | -62.62 | 51.49 | 0.675 |
| *Millepora alcicornis* | *Aluterus scriptus* | 1 | -0.51 | -3.36 | 2.64 | -30.98 | -73.43 | -0.82 | 2.21 | -10.92 | 13.78 | 0.202 |
| *Millepora alcicornis* | *Melichthys niger* | 1 | -3.7 | -6.06 | -2.1 | -2.81 | -8.43 | 0.28 | -0.28 | -1.24 | 0.71 | 0.391 |
| *Millepora alcicornis* | *Rypticus saponaceus* | 1 | 0 | -2.92 | 2.81 | 14.18 | -4.02 | 42.62 | 31.1 | 1.92 | 69.37 | 0.809 |
| *Millepora alcicornis* | *Mulloidichthys martinicus* | 1 | -1.17 | -2.94 | 1.41 | 3.05 | -0.05 | 9.44 | 0.32 | -1.3 | 1.8 | 0.537 |
| *Millepora alcicornis* | *Caranx latus* | 1 | 0.21 | -2.84 | 3.27 | 19.95 | 0.75 | 46.21 | 13.1 | 1.38 | 43.55 | 0.501 |
| *Millepora alcicornis* | *Diodon hystrix* | 1 | 0.4 | -2.33 | 3.39 | 2.78 | -11.15 | 16.08 | -20.88 | -71.89 | 4.74 | 0.516 |
| *Millepora alcicornis* | *Cryptotomus roseus* | 1 | 0.94 | -0.76 | 3.45 | 8.74 | 3.7 | 16.89 | 0.88 | -0.39 | 2.6 | 0.002 |
| *Millepora alcicornis* | *Sparisoma tuiupiranga* | 1 | 1.43 | -1.23 | 4.94 | 8.39 | 0.67 | 18.42 | 0.16 | -0.8 | 1.3 | 0.034 |
| *Millepora alcicornis* | *Odontoscion dentex* | 1 | -2.64 | -5.8 | 0.79 | -4.33 | -18.7 | 8.82 | 10.76 | 0.6 | 48.26 | 0.457 |
| *Millepora alcicornis* | *Aluterus monoceros* | 1 | -0.06 | -3.17 | 3.01 | 8.92 | -42.01 | 58.78 | 21.46 | 0.37 | 58.27 | 0.671 |
| *Millepora alcicornis* | *Haemulon steindachneri* | 1 | -0.98 | -4.57 | 2.07 | 6.47 | -20.06 | 35.06 | 34.6 | 8.6 | 71.63 | 0.507 |
| *Millepora alcicornis* | *Lactophrys trigonus* | 1 | -0.15 | -3.34 | 2.89 | 8.29 | -45.14 | 58.66 | 22.23 | 0.48 | 56.12 | 0.662 |
| *Millepora alcicornis* | *Mycteroperca acutirostris* | 1 | -0.35 | -1.66 | 1.37 | 1.21 | -0.31 | 4.17 | 6.51 | 1.49 | 19.79 | 0.218 |
| *Millepora alcicornis* | *Synodus intermedius* | 1 | 0.15 | -2.69 | 3.04 | 30.52 | 4.16 | 65.44 | 17.61 | 1.64 | 44.83 | 0.668 |
| *Millepora alcicornis* | *Balistes vetula* | 1 | -1.18 | -4.4 | 2.89 | 12.51 | 0.47 | 42.67 | 10.18 | -1.13 | 49.88 | 0.202 |
| *Millepora alcicornis* | *Canthigaster figueiredoi* | 1 | 2.48 | 0.42 | 5.29 | 5.48 | 0.78 | 12.1 | 3.67 | 0.84 | 8.36 | 0.001 |
| *Millepora alcicornis* | *Malacoctenus delalandii* | 1 | -0.08 | -3.33 | 2.98 | -7.13 | -52.56 | 38.25 | 23.28 | -10.94 | 63.06 | 0.639 |
| *Millepora alcicornis* | *Anisotremus moricandi* | 1 | -1.52 | -5.13 | 2.4 | -9.08 | -40.34 | 6.23 | -2.3 | -51.84 | 49.93 | 0.354 |
| *Millepora alcicornis* | *Chilomycterus spinosus spinosus* | 1 | -0.3 | -3.11 | 2.48 | 43.69 | 15.81 | 80.27 | 3.39 | -7.16 | 16.86 | 0.169 |
| *Millepora alcicornis* | *Diplodus argenteus* | 1 | -0.42 | -1.13 | 0.24 | 0.43 | -0.25 | 1.21 | 1.49 | 0.6 | 2.58 | 0.284 |
| *Millepora alcicornis* | *Sphoeroides spengleri* | 1 | 1.12 | -0.53 | 3.4 | 1.18 | -0.49 | 4.88 | 3.37 | 0.75 | 7.12 | 0.093 |
| *Millepora alcicornis* | *Dactylopterus volitans* | 1 | -0.55 | -4.33 | 2.69 | -15.62 | -51.46 | 7.47 | 10.36 | -44.87 | 57.75 | 0.545 |
| *Millepora alcicornis* | *Kyphosus sectatrix* | 1 | -0.01 | -2.99 | 2.91 | -23.29 | -56.34 | -1.8 | -6.25 | -56.8 | 55.97 | 0.513 |
| *Millepora alcicornis* | *Aulostomus strigosus* | 1 | -0.13 | -3.37 | 2.81 | -18.27 | -51.15 | 4.46 | -3.36 | -61.15 | 55.86 | 0.616 |
| *Millepora alcicornis* | *Caranx crysos* | 1 | 0.9 | -1.9 | 3.85 | 5.77 | -2.79 | 16.6 | -11.56 | -57.87 | 18.53 | 0.635 |
| *Millepora alcicornis* | *Fistularia tabacaria* | 1 | -0.12 | -3.15 | 2.85 | 18.55 | 1.5 | 47.26 | 29.14 | 0.63 | 66.31 | 0.549 |
| *Millepora alcicornis* | *Chaetodon sedentarius* | 1 | -2.03 | -3.12 | -1.11 | 1.48 | 0.49 | 2.63 | 0.07 | -0.96 | 1.2 | 0.642 |
| *Millepora alcicornis* | *Priacanthus arenatus* | 1 | -2.5 | -5.89 | 1.62 | -8.31 | -37.65 | 1.44 | -5.74 | -49.2 | 24.37 | 0.334 |
| *Millepora alcicornis* | *Bodianus pulchellus* | 1 | -1.48 | -2.33 | -0.73 | 0.88 | 0.1 | 1.81 | 0.39 | -0.43 | 1.33 | 0.095 |
| *Millepora alcicornis* | *Elacatinus figaro* | 1 | -0.45 | -3.46 | 2.43 | 37.5 | 9.58 | 76.63 | 2.61 | -15.35 | 21.09 | 0.665 |
| *Millepora alcicornis* | *Sphoeroides greeleyi* | 1 | -0.47 | -3.49 | 2.4 | 39.07 | 8.69 | 77.63 | 2.48 | -17.59 | 22.9 | 0.67 |
| *Millepora alcicornis* | *Stephanolepis hispidus* | 1 | -0.92 | -1.84 | 0.72 | 1.74 | 0.24 | 7.03 | 0.52 | -0.38 | 1.81 | 0.008 |
| *Millepora alcicornis* | *Epinephelus morio* | 1 | -0.1 | -3.05 | 2.7 | 15.42 | 2.01 | 36.44 | 32.44 | 3.72 | 72.25 | 0.232 |
| *Millepora alcicornis* | *Heteropriacanthus cruentatus* | 1 | -1.26 | -5.64 | 2.16 | 27.76 | 1.07 | 66.94 | -1.45 | -33.51 | 19.58 | 0.347 |
| *Millepora alcicornis* | *Labrisomus nuchipinnis* | 1 | -0.21 | -3.21 | 2.79 | 21.18 | 1.68 | 51.18 | 28.25 | 1.02 | 66.59 | 0.409 |
| *Millepora alcicornis* | *Myrichthys ocellatus* | 1 | -1.31 | -5.79 | 2.14 | 28.24 | 0.99 | 71.34 | -0.81 | -33.85 | 19.84 | 0.364 |
| *Millepora alcicornis* | *Acanthostracion quadricornis* | 1 | -0.59 | -3.98 | 2.39 | 37.01 | 6.57 | 74.09 | 2.69 | -16.07 | 21.05 | 0.518 |
| *Millepora alcicornis* | *Epinephelus marginatus* | 1 | -3.14 | -6.29 | -0.42 | -6.82 | -15.79 | -1.09 | 6.77 | 1.1 | 31.63 | 0.131 |
| *Millepora alcicornis* | *Mugil curema* | 1 | -0.18 | -2.97 | 2.65 | 40.63 | 10.78 | 77.82 | 0.81 | -17.16 | 16.49 | 0.511 |
| *Millepora alcicornis* | *Orthopristis ruber* | 1 | -0.22 | -3.16 | 2.64 | 22.74 | 1.36 | 53.25 | 28.63 | 6.32 | 61.21 | 0.157 |
| *Millepora alcicornis* | *Kyphosus vaigiensis* | 1 | -2.98 | -6.76 | 1.37 | 0.55 | -24.02 | 37.1 | 9.53 | -0.51 | 46.87 | 0.147 |
| *Millepora alcicornis* | *Mycteroperca interstitialis* | 1 | -1.03 | -5.21 | 2.36 | 6.99 | -29.14 | 45.67 | 21.13 | -0.41 | 57.07 | 0.526 |
| *Millepora alcicornis* | *Eucinostomus melanopterus* | 1 | -0.62 | -4.3 | 2.52 | 9.43 | -42.06 | 60.02 | 23.37 | 0.68 | 60.51 | 0.604 |
| *Millepora alcicornis* | *Lutjanus cyanopterus* | 1 | -1.65 | -6.23 | 2.1 | 11.33 | -27.97 | 56.93 | 15.92 | 0.28 | 48.41 | 0.416 |
| *Millepora alcicornis* | *Calamus penna* | 1 | -0.18 | -3.51 | 2.77 | -17.97 | -50.81 | 3.28 | -5.74 | -62.66 | 51.86 | 0.601 |
| *Millepora alcicornis* | *Seriola rivoliana* | 1 | -0.19 | -3.55 | 3.01 | -18.34 | -52.84 | 2.61 | -5.69 | -62.02 | 49.13 | 0.606 |
| *Millepora alcicornis* | *Parablennius pilicornis* | 1 | -0.31 | -3.72 | 2.63 | -11.35 | -55.98 | 32.46 | 18.9 | -20.53 | 58.34 | 0.753 |
| *Millepora alcicornis* | *Pareques acuminatus* | 1 | -0.16 | -3.43 | 2.93 | -9.56 | -55.82 | 32.1 | 23.56 | -16.18 | 63.95 | 0.745 |
| *Millepora alcicornis* | *Rhomboplites aurorubens* | 1 | -4.03 | -6.71 | -2.06 | -1.83 | -7.24 | 1.37 | 1.11 | -0.67 | 3.4 | 0.421 |
| *Millepora alcicornis* | *Sphoeroides testudineus* | 1 | -0.17 | -3.24 | 2.74 | -8.86 | -50.59 | 27.34 | 23.95 | -24.74 | 66.4 | 0.798 |
| *Millepora alcicornis* | *Myripristis jacobus* | 1 | -0.54 | -3.98 | 2.38 | 34.01 | 3.53 | 74.19 | 0.12 | -34.99 | 27.17 | 0.708 |
| *Millepora alcicornis* | *Acanthurus bahianus* | 2 | -0.14 | -1.61 | 2.45 | 2.3 | -0.52 | 9.18 | 0.97 | -0.05 | 2.15 | 0.007 |
| *Millepora alcicornis* | *Acanthurus chirurgus* | 2 | -3.07 | -5.44 | -1.44 | -4.05 | -9.89 | -0.39 | -1.07 | -1.94 | -0.29 | 0.606 |
| *Millepora alcicornis* | *Sparisoma frondosum* | 2 | 1.68 | -0.88 | 4.89 | 7.76 | 0.45 | 16.11 | -2.12 | -5.59 | -0.31 | 0.383 |
| *Millepora alcicornis* | *Sparisoma axillare* | 2 | -0.29 | -3.6 | 3.38 | -19.68 | -60.12 | 0.28 | -10.14 | -29.87 | 0.45 | 0.347 |
| *Millepora alcicornis* | *Sparisoma radians* | 2 | -0.14 | -1.52 | 1.85 | 3.46 | 0.24 | 8.92 | -0.95 | -2.11 | -0.01 | 0.368 |
| *Millepora alcicornis* | *Stegastes variabilis* | 2 | 1.24 | -1.5 | 4.59 | -6.03 | -30.08 | 8.18 | 0.34 | -2.63 | 4.13 | 0.535 |
| *Millepora alcicornis* | *Abudefduf saxatilis* | 2 | 2.59 | 0.97 | 5.03 | 3.7 | 0.18 | 9.49 | 0.95 | 0.13 | 1.83 | 0.036 |
| *Millepora alcicornis* | *Acanthurus coeruleus* | 2 | -1.57 | -2.87 | -0.31 | -1.73 | -4.82 | 0.14 | -1.07 | -2.72 | -0.01 | 0.096 |
| *Millepora alcicornis* | *Chromis multilineata* | 2 | 0.87 | -0.8 | 3.28 | 3.03 | -0.22 | 8.17 | 0.13 | -1.05 | 1.28 | 0.035 |
| *Millepora alcicornis* | *Halichoeres brasiliensis* | 2 | -1.36 | -4.26 | 2.33 | -18.81 | -62.12 | 0.32 | 8.18 | -3.37 | 36.63 | 0.357 |
| *Millepora alcicornis* | *Halichoeres poeyi* | 2 | 1.4 | -0.07 | 3.22 | 6.9 | 2.91 | 12.02 | 0.4 | -0.38 | 1.27 | 0.008 |
| *Millepora alcicornis* | *Holacanthus tricolor* | 2 | -0.08 | -3.13 | 2.88 | -4.9 | -49.09 | 32.69 | -32.07 | -77.94 | 11.53 | 0.779 |
| *Millepora alcicornis* | *Microspathodon chrysurus* | 2 | -0.53 | -4.25 | 2.57 | 6.43 | -39.66 | 50.77 | -24.9 | -61.6 | 0.05 | 0.668 |
| *Millepora alcicornis* | *Anisotremus virginicus* | 2 | 0.18 | -2.36 | 3.61 | 3.16 | -1.47 | 10.03 | 0.75 | -5.02 | 6.22 | 0.405 |
| *Millepora alcicornis* | *Haemulon aurolineatum* | 2 | -0.62 | -2.13 | 1.92 | 0.14 | -3.22 | 6.66 | 2.84 | 0.6 | 8.56 | 0.289 |
| *Millepora alcicornis* | *Stegastes fuscus* | 2 | 0.42 | -0.93 | 2.19 | 5.01 | 1.08 | 9.94 | 1.12 | 0.09 | 2.37 | 0.107 |
| *Millepora alcicornis* | *Stegastes rocasensis* | 2 | -2.76 | -5.11 | -1.12 | -4.37 | -10.22 | -0.53 | -0.13 | -0.84 | 0.62 | 0.116 |
| *Millepora alcicornis* | *Thalassoma noronhanum* | 2 | -2.89 | -5.3 | -1.37 | -4.05 | -10.01 | -0.5 | -0.13 | -0.84 | 0.6 | 0.212 |
| *Millepora alcicornis* | *Halichoeres radiatus* | 2 | 0.98 | -1.29 | 3.69 | -25.18 | -64.12 | -0.61 | 4.5 | -0.49 | 11.75 | 0.189 |
| *Millepora alcicornis* | *Cantherhines pullus* | 2 | -0.42 | -3.5 | 2.36 | 40.38 | 11.77 | 77.76 | 3.68 | -8.69 | 17.74 | 0.132 |
| *Millepora alcicornis* | *Cryptotomus roseus* | 2 | -0.52 | -2.63 | 2.64 | 15.04 | -0.5 | 53.13 | 12.48 | -0.12 | 46.19 | 0.015 |
| *Millepora alcicornis* | *Haemulon plumierii* | 2 | -2.78 | -5.8 | 1.51 | 5.99 | -6.62 | 48.14 | 5.97 | -0.59 | 31.22 | 0.33 |
| *Millepora alcicornis* | *Canthigaster figueiredoi* | 2 | 0.17 | -2.62 | 3.13 | 23.22 | 3.94 | 49.8 | 29.13 | 2.33 | 63.23 | 0.433 |
| *Millepora alcicornis* | *Chaetodon striatus* | 2 | -0.34 | -3.64 | 2.72 | -1.73 | -47.17 | 40.81 | 27.35 | 0.62 | 60.99 | 0.356 |
| *Millepora alcicornis* | *Mycteroperca bonaci* | 2 | -0.14 | -3.43 | 2.87 | 7.6 | -48.1 | 60.86 | 23.58 | -0.06 | 61.82 | 0.659 |
| *Millepora alcicornis* | *Pomacanthus paru* | 2 | -0.09 | -2.83 | 2.8 | -20.48 | -52.53 | -0.14 | 28.62 | 2.77 | 64.84 | 0.307 |
| *Millepora alcicornis* | *Scarus zelindae* | 2 | 0.46 | -2.51 | 4.37 | 17.82 | 2.54 | 43.38 | 5.12 | -0.09 | 15.81 | 0.1 |
| *Millepora alcicornis* | *Sphoeroides spengleri* | 2 | -0.16 | -1.53 | 1.88 | 4.17 | -0.24 | 22.64 | 8.09 | 0.42 | 46.06 | 0.064 |
| *Millepora alcicornis* | *Ocyurus chrysurus* | 2 | -0.07 | -3.42 | 3 | -4.86 | -55.27 | 39.64 | 17.03 | -16.75 | 55.77 | 0.629 |
| *Millepora alcicornis* | *Labrisomus nuchipinnis* | 2 | -0.24 | -3.37 | 2.69 | 0.86 | -52.14 | 45.2 | 26.25 | -8.98 | 63.47 | 0.724 |
| *Millepora alcicornis* | *Bodianus pulchellus* | 2 | 0.03 | -2.72 | 2.93 | 23.04 | 2.85 | 55.08 | 28.52 | 2.24 | 63.67 | 0.665 |
| *Millepora alcicornis* | *Chilomycterus spinosus spinosus* | 2 | -0.41 | -3.52 | 2.45 | 38.05 | 8.84 | 77.8 | 2.67 | -19.09 | 22.68 | 0.662 |
| *Millepora alcicornis* | *Holocentrus adscensionis* | 2 | -0.52 | -3.73 | 2.38 | 38.25 | 8.7 | 74.69 | 2.92 | -10.81 | 18.48 | 0.313 |
| *Millepora alcicornis* | *Pseudupeneus maculatus* | 2 | -1.29 | -3.34 | 1.42 | 7.57 | 0.48 | 21.78 | 2.82 | -0.59 | 12.67 | 0.224 |
| *Millepora alcicornis* | *Serranus baldwini* | 2 | 0.3 | -1.89 | 3.28 | 8.5 | 0.7 | 20.67 | 2.65 | -0.41 | 10.49 | 0.168 |
| *Millepora alcicornis* | *Stephanolepis hispidus* | 2 | -2.48 | -4.13 | -0.77 | 7.51 | 0.75 | 40.59 | 1.3 | -1.16 | 10.29 | 0.363 |
| *Millepora alcicornis* | *Bodianus rufus* | 2 | -1.95 | -3.98 | 0.67 | 4.3 | 0 | 16.96 | 2.43 | -0.48 | 6.2 | 0.394 |
| *Millepora alcicornis* | *Diplodus argenteus* | 2 | 0.11 | -2.58 | 3.03 | 16.75 | 2.91 | 38.81 | 36.96 | 10.14 | 71.52 | 0.404 |
| *Millepora alcicornis* | *Halichoeres dimidiatus* | 2 | -1.75 | -5.54 | 1.89 | 27.02 | 1.12 | 73.42 | 1.43 | -6.98 | 15 | 0.171 |
| *Millepora alcicornis* | *Sparisoma tuiupiranga* | 2 | -0.83 | -4.46 | 2.3 | 35.67 | 3.23 | 75.26 | 2.71 | -11.28 | 17.99 | 0.256 |
| *Millepora alcicornis* | *Eucinostomus melanopterus* | 2 | -4.26 | -7.37 | -1.83 | 0.33 | -7 | 6.24 | 2.7 | -0.26 | 7.41 | 0.249 |
| *Millepora alcicornis* | *Sphoeroides greeleyi* | 2 | -0.3 | -3.64 | 2.73 | -0.26 | -48.27 | 44.87 | 27 | -2.92 | 64.74 | 0.716 |
| *Millepora alcicornis* | *Sphoeroides testudineus* | 2 | -0.27 | -3.57 | 2.71 | -0.72 | -49.2 | 44.03 | 26.84 | -3.35 | 64.72 | 0.724 |
| *Millepora alcicornis* | *Coryphopterus glaucofraenum* | 2 | -0.16 | -3.27 | 2.84 | -4.51 | -51.34 | 38.87 | 23.46 | -13.9 | 60.11 | 0.63 |
| *Millepora alcicornis* | *Synodus synodus* | 2 | -0.15 | -3.37 | 2.98 | -8.19 | -58.07 | 36.97 | 21.22 | -22.65 | 63.78 | 0.749 |
| *Millepora alcicornis* | *Pareques acuminatus* | 2 | -0.1 | -3.29 | 2.8 | -10.11 | -58.64 | 29.53 | 23.52 | -27.92 | 66.3 | 0.807 |
| *Millepora alcicornis* | *Sparisoma amplum* | 2 | -0.6 | -4 | 2.4 | 35.07 | 3.44 | 75.89 | 0.33 | -33.96 | 23.58 | 0.674 |
| *Montastraea cavernosa* | *Acanthurus chirurgus* | 1 | 0.92 | 0.24 | 1.71 | -0.68 | -1.72 | 0.1 | -0.67 | -1.63 | 0.16 | 0 |
| *Montastraea cavernosa* | *Chaetodon ocellatus* | 1 | -2.59 | -4.19 | -1.39 | -2.93 | -8.52 | 0 | -0.91 | -1.83 | -0.08 | 0.626 |
| *Montastraea cavernosa* | *Haemulon plumierii* | 1 | 1.3 | -0.84 | 4.35 | 6.25 | -0.67 | 16.33 | -2.21 | -7.7 | -0.08 | 0.02 |
| *Montastraea cavernosa* | *Halichoeres dimidiatus* | 1 | -0.76 | -3.12 | 2.13 | -3.25 | -20.89 | 10.89 | -3.13 | -13.28 | 0.02 | 0.078 |
| *Montastraea cavernosa* | *Ocyurus chrysurus* | 1 | 1.22 | -0.64 | 3.65 | 11.84 | 3.52 | 22.72 | 0.03 | -0.8 | 0.9 | 0.002 |
| *Montastraea cavernosa* | *Pomacanthus arcuatus* | 1 | -0.25 | -2.56 | 2.21 | 16.27 | 3.76 | 30.03 | 0.68 | -1.13 | 2.76 | 0.054 |
| *Montastraea cavernosa* | *Pseudupeneus maculatus* | 1 | 1.95 | 0.71 | 4.17 | 0.12 | -2.13 | 6.65 | -1.22 | -3.15 | 0.12 | 0.014 |
| *Montastraea cavernosa* | *Sparisoma axillare* | 1 | 2.59 | 1.22 | 4.81 | 4.91 | 0.11 | 13.44 | -0.85 | -1.9 | 0.07 | 0.002 |
| *Montastraea cavernosa* | *Sparisoma frondosum* | 1 | 2.33 | 0.91 | 4.58 | 5.67 | 0.47 | 14.94 | 0.12 | -0.6 | 0.82 | 0.032 |
| *Montastraea cavernosa* | *Thalassoma noronhanum* | 1 | -1.68 | -3.15 | -0.65 | -3.73 | -9.78 | -0.32 | -0.74 | -1.45 | -0.07 | 0 |
| *Montastraea cavernosa* | *Abudefduf saxatilis* | 1 | 5.03 | 2.76 | 8.22 | 3.58 | -0.73 | 11.97 | -0.21 | -2.63 | 1.57 | 0 |
| *Montastraea cavernosa* | *Acanthurus bahianus* | 1 | 2.49 | 1.09 | 4.76 | 5.61 | 0.42 | 14.87 | 0.23 | -0.47 | 0.92 | 0.009 |
| *Montastraea cavernosa* | *Acanthurus coeruleus* | 1 | 1.73 | 0.26 | 3.95 | 6.59 | 0.94 | 15.75 | -0.94 | -1.93 | -0.14 | 0.063 |
| *Montastraea cavernosa* | *Anisotremus virginicus* | 1 | 2.78 | 1.28 | 5.07 | 4.72 | 0.09 | 13.45 | -0.18 | -1.87 | 1.03 | 0.008 |
| *Montastraea cavernosa* | *Bodianus rufus* | 1 | 0.76 | -0.2 | 2.52 | 0.49 | -1.72 | 7.04 | 0.51 | -0.21 | 1.32 | 0.009 |
| *Montastraea cavernosa* | *Carangoides bartholomaei* | 1 | -1.09 | -2.64 | 0.19 | -5.62 | -12.53 | -0.48 | -0.5 | -1.37 | 0.41 | 0.03 |
| *Montastraea cavernosa* | *Caranx ruber* | 1 | 0.2 | -3 | 3.31 | 10.67 | -17.24 | 30.37 | -1.47 | -7.89 | 0.97 | 0.401 |
| *Montastraea cavernosa* | *Haemulon aurolineatum* | 1 | 2.05 | 0.61 | 4.17 | 6.38 | 0.74 | 15.21 | 0.3 | -0.38 | 0.96 | 0 |
| *Montastraea cavernosa* | *Haemulon parra* | 1 | -0.52 | -2.76 | 2.25 | -14.93 | -55.34 | -0.74 | -2.72 | -10.57 | -0.04 | 0.477 |
| *Montastraea cavernosa* | *Halichoeres bivittatus* | 1 | -0.26 | -3.27 | 2.71 | 14.57 | -18.93 | 48.07 | -29.93 | -65 | -2.06 | 0.265 |
| *Montastraea cavernosa* | *Halichoeres brasiliensis* | 1 | 2.17 | 0.5 | 4.56 | 7.95 | 1.21 | 18.41 | 0.85 | 0.16 | 1.63 | 0.001 |
| *Montastraea cavernosa* | *Halichoeres poeyi* | 1 | 1.41 | 0.41 | 3.02 | 0.2 | -1.74 | 6.14 | 0.31 | -0.53 | 1.2 | 0.009 |
| *Montastraea cavernosa* | *Holacanthus ciliaris* | 1 | 1.09 | -0.65 | 3.42 | 10.21 | 2.87 | 20.25 | -0.79 | -2.29 | 0.2 | 0.221 |
| *Montastraea cavernosa* | *Holocentrus adscensionis* | 1 | 1.36 | 0.54 | 2.3 | -0.68 | -1.87 | 0.18 | 0.16 | -0.69 | 1.01 | 0 |
| *Montastraea cavernosa* | *Lutjanus jocu* | 1 | 1.04 | -0.81 | 3.46 | 9.13 | 0.77 | 19.78 | -0.48 | -1.35 | 0.31 | 0.059 |
| *Montastraea cavernosa* | *Pomacanthus paru* | 1 | 1.72 | 0.15 | 4.02 | 7.66 | 1.25 | 17.28 | 0.09 | -0.59 | 0.77 | 0.009 |
| *Montastraea cavernosa* | *Scarus trispinosus* | 1 | 0.95 | -1.06 | 3.53 | 16.43 | 5.25 | 35.85 | -4.15 | -22.36 | -0.02 | 0.004 |
| *Montastraea cavernosa* | *Scarus zelindae* | 1 | 1.47 | -0.2 | 3.87 | 9.15 | 1.91 | 19.38 | -0.07 | -0.79 | 0.63 | 0.001 |
| *Montastraea cavernosa* | *Sparisoma amplum* | 1 | -0.71 | -2.59 | 2.29 | -8.62 | -46.98 | 6.77 | -0.59 | -5.76 | 1.28 | 0.163 |
| *Montastraea cavernosa* | *Sparisoma radians* | 1 | 0.48 | -2.15 | 3.56 | -31.21 | -69.7 | -1.13 | -3.99 | -11.57 | 0.92 | 0.697 |
| *Montastraea cavernosa* | *Sphyraena barracuda* | 1 | -0.15 | -3.31 | 2.77 | 16.43 | -1.74 | 35.94 | -5.61 | -34.57 | 0.16 | 0.249 |
| *Montastraea cavernosa* | *Stegastes variabilis* | 1 | 1.85 | -0.65 | 5.12 | 13.57 | 2.21 | 28.19 | 1.17 | -0.03 | 2.53 | 0.371 |
| *Montastraea cavernosa* | *Chaetodon striatus* | 1 | 2.58 | 1.13 | 4.69 | 3.27 | 0.04 | 9.55 | 0.92 | -0.17 | 2.24 | 0 |
| *Montastraea cavernosa* | *Lutjanus apodus* | 1 | -0.58 | -3.85 | 2.32 | 16.26 | -31.02 | 60.66 | -22.4 | -64.29 | -1.82 | 0.705 |
| *Montastraea cavernosa* | *Stegastes fuscus* | 1 | 1.73 | 0.7 | 3.2 | 3.7 | 0.32 | 9.68 | 0.76 | 0.09 | 1.47 | 0.019 |
| *Montastraea cavernosa* | *Acanthostracion polygonius* | 1 | 0 | -2.86 | 3.03 | -17.25 | -50.46 | 7.13 | -30.78 | -68.67 | 3.77 | 0.397 |
| *Montastraea cavernosa* | *Amblycirrhitus pinos* | 1 | -0.76 | -4.48 | 2.19 | 11.62 | -18.86 | 45.94 | -23.88 | -64.28 | 1.07 | 0.465 |
| *Montastraea cavernosa* | *Anisotremus surinamensis* | 1 | 0.17 | -2.68 | 3.31 | -30.11 | -72.88 | 2.37 | 3.05 | -4.53 | 11.65 | 0.349 |
| *Montastraea cavernosa* | *Aulostomus maculatus* | 1 | -0.26 | -3.44 | 2.68 | 7.4 | -39.36 | 47.49 | -31.6 | -68.39 | -0.99 | 0.721 |
| *Montastraea cavernosa* | *Cantherhines pullus* | 1 | -2.38 | -4.63 | -0.96 | -5.64 | -15.05 | -0.34 | -0.27 | -0.99 | 0.45 | 0.105 |
| *Montastraea cavernosa* | *Canthidermis maculata* | 1 | -0.17 | -2.29 | 2.22 | 16.92 | 6.4 | 31.05 | 0.11 | -1.68 | 2.06 | 0.24 |
| *Montastraea cavernosa* | *Cephalopholis fulva* | 1 | -2.43 | -4.75 | -0.94 | -5.64 | -15.14 | -0.43 | -0.32 | -1.04 | 0.44 | 0.195 |
| *Montastraea cavernosa* | *Chromis multilineata* | 1 | -1.05 | -3.39 | 0.78 | -8.68 | -19.87 | -1.14 | 1.09 | -0.18 | 2.58 | 0.006 |
| *Montastraea cavernosa* | *Clepticus brasiliensis* | 1 | -0.79 | -3.73 | 2.16 | -4.12 | -29.1 | 10.56 | -0.19 | -3.53 | 2.67 | 0.412 |
| *Montastraea cavernosa* | *Epinephelus adscensionis* | 1 | -1.48 | -4.08 | 1.61 | -15.51 | -59.22 | -0.46 | 4.06 | -1.07 | 23.89 | 0.591 |
| *Montastraea cavernosa* | *Haemulon squamipinna* | 1 | -2.34 | -4.65 | 0.09 | -5.98 | -18.76 | -0.04 | -1.95 | -10.74 | 0.44 | 0.396 |
| *Montastraea cavernosa* | *Halichoeres penrosei* | 1 | -0.31 | -3.15 | 2.61 | -14.09 | -40.2 | 5.38 | -32.35 | -69.76 | -6.79 | 0.047 |
| *Montastraea cavernosa* | *Microspathodon chrysurus* | 1 | -2.19 | -4.37 | -0.65 | -5.63 | -14.63 | -0.23 | 0.06 | -0.91 | 0.98 | 0.62 |
| *Montastraea cavernosa* | *Ophioblennius macclurei* | 1 | -0.13 | -3.02 | 2.74 | 5.25 | -38.75 | 46.85 | -31.55 | -71.29 | -1.76 | 0.795 |
| *Montastraea cavernosa* | *Scomberomorus regalis* | 1 | -0.27 | -3.38 | 2.58 | 2.71 | -45.7 | 45.6 | -31.16 | -71.62 | 0.89 | 0.782 |
| *Montastraea cavernosa* | *Serranus baldwini* | 1 | -1.89 | -4.17 | 0.44 | -8.72 | -36.22 | -0.38 | 1.05 | -0.69 | 9.84 | 0.022 |
| *Montastraea cavernosa* | *Halichoeres radiatus* | 1 | -2.07 | -4.28 | -0.59 | -6.16 | -15.57 | -0.62 | -0.34 | -1.02 | 0.33 | 0 |
| *Montastraea cavernosa* | *Holacanthus tricolor* | 1 | -1.24 | -4.12 | 1.83 | -4.34 | -28.55 | 10.63 | -4.49 | -30.81 | 0.86 | 0.337 |
| *Montastraea cavernosa* | *Lutjanus analis* | 1 | -0.41 | -3.52 | 2.58 | 17.3 | -33.37 | 62.94 | -23.5 | -57.83 | -2.84 | 0.721 |
| *Montastraea cavernosa* | *Mycteroperca bonaci* | 1 | 0.24 | -1.71 | 2.55 | 12.55 | 4.15 | 23.59 | -0.06 | -1.52 | 1.41 | 0.64 |
| *Montastraea cavernosa* | *Pseudocaranx dentex* | 1 | 0.01 | -2.81 | 3.08 | -23.67 | -60.36 | 5.35 | 16.39 | 1.45 | 40.94 | 0.473 |
| *Montastraea cavernosa* | *Bothus ocellatus* | 1 | -0.6 | -4.21 | 2.41 | 15.35 | -35.07 | 55.42 | 15.97 | 0.62 | 54.92 | 0.518 |
| *Montastraea cavernosa* | *Coryphopterus glaucofraenum* | 1 | -0.08 | -2.99 | 3.25 | -28.99 | -70.99 | -0.79 | -1.04 | -8.32 | 3.86 | 0.506 |
| *Montastraea cavernosa* | *Malacanthus plumieri* | 1 | -2.75 | -4.82 | -1.39 | -4.66 | -13.15 | -0.09 | 0.32 | -0.54 | 1.23 | 0.708 |
| *Montastraea cavernosa* | *Stegastes rocasensis* | 1 | -2.63 | -4.72 | -1.24 | -4.92 | -13.38 | -0.2 | -0.26 | -0.99 | 0.54 | 0.43 |
| *Montastraea cavernosa* | *Apogon americanus* | 1 | 0.02 | -3.18 | 3.1 | -20.87 | -58.59 | 13.09 | -11.59 | -53.96 | 41.85 | 0.672 |
| *Montastraea cavernosa* | *Aluterus scriptus* | 1 | -0.21 | -2.36 | 2.42 | 6.13 | -2.49 | 18.95 | 0.44 | -0.8 | 1.84 | 0.206 |
| *Montastraea cavernosa* | *Melichthys niger* | 1 | -3.69 | -5.84 | -2.13 | -4.16 | -12.36 | 0.15 | -0.36 | -1.37 | 0.74 | 0.408 |
| *Montastraea cavernosa* | *Rypticus saponaceus* | 1 | -0.2 | -3.32 | 2.7 | -2.25 | -49.21 | 40.2 | 28.53 | 2.7 | 63.58 | 0.815 |
| *Montastraea cavernosa* | *Mulloidichthys martinicus* | 1 | -1 | -4.18 | 2.31 | -11.26 | -57.43 | 12.2 | 0.13 | -5.21 | 4.75 | 0.433 |
| *Montastraea cavernosa* | *Caranx latus* | 1 | -0.47 | -3.35 | 2.38 | 17.78 | -0.35 | 47.24 | 18.94 | 1.32 | 55.4 | 0.384 |
| *Montastraea cavernosa* | *Diodon hystrix* | 1 | -0.05 | -3.28 | 2.88 | -14.1 | -50.61 | 11.67 | -27.46 | -69.57 | 8.86 | 0.43 |
| *Montastraea cavernosa* | *Cryptotomus roseus* | 1 | 0.12 | -1.2 | 2.04 | 4.05 | -0.6 | 12.31 | 0.24 | -0.5 | 1.01 | 0.002 |
| *Montastraea cavernosa* | *Sparisoma tuiupiranga* | 1 | 0.35 | -0.97 | 2.09 | 7.22 | 1.79 | 14.52 | 0.07 | -0.71 | 0.87 | 0.132 |
| *Montastraea cavernosa* | *Odontoscion dentex* | 1 | -1.77 | -4.9 | 1.61 | 6.33 | -13.91 | 40.99 | 15.63 | 0.72 | 52.63 | 0.338 |
| *Montastraea cavernosa* | *Aluterus monoceros* | 1 | -0.62 | -3.58 | 2.07 | 43.01 | 12.52 | 81.96 | 4.51 | -3 | 14.8 | 0.422 |
| *Montastraea cavernosa* | *Haemulon steindachneri* | 1 | -0.44 | -3.61 | 2.32 | 23.86 | -1.8 | 55.52 | 33.86 | 10.67 | 65.35 | 0.48 |
| *Montastraea cavernosa* | *Lactophrys trigonus* | 1 | -0.61 | -3.51 | 2.28 | 43.63 | 14.33 | 80.89 | 3.2 | -3.13 | 11.34 | 0.447 |
| *Montastraea cavernosa* | *Mycteroperca acutirostris* | 1 | 0.55 | -1.16 | 2.97 | 5.75 | -0.34 | 16.34 | 3.15 | 1.16 | 6.21 | 0.271 |
| *Montastraea cavernosa* | *Synodus intermedius* | 1 | 0.12 | -2.48 | 2.88 | 2.31 | -32.52 | 26.99 | 19.79 | -0.99 | 64.07 | 0.658 |
| *Montastraea cavernosa* | *Balistes vetula* | 1 | -0.12 | -2.87 | 2.75 | 19.44 | 3.28 | 43.92 | -23.65 | -61.55 | 1.32 | 0.183 |
| *Montastraea cavernosa* | *Canthigaster figueiredoi* | 1 | 1.52 | -0.36 | 4.15 | 1.83 | -1.87 | 9.61 | 2.38 | 0.29 | 6.11 | 0.001 |
| *Montastraea cavernosa* | *Malacoctenus delalandii* | 1 | -0.67 | -3.82 | 2.22 | 42.65 | 12.48 | 81.86 | 2.78 | -4.17 | 11.21 | 0.337 |
| *Montastraea cavernosa* | *Anisotremus moricandi* | 1 | -1.23 | -5.11 | 2.19 | -0.42 | -32.9 | 23.95 | -1.41 | -40.83 | 32.04 | 0.438 |
| *Montastraea cavernosa* | *Chilomycterus spinosus spinosus* | 1 | -0.23 | -3.38 | 3.03 | -24.56 | -63.71 | -0.08 | -7.82 | -25.36 | 8.36 | 0.175 |
| *Montastraea cavernosa* | *Diplodus argenteus* | 1 | -1.67 | -3.8 | -0.28 | -6.49 | -15.12 | -1.02 | 1.36 | 0.48 | 2.38 | 0.288 |
| *Montastraea cavernosa* | *Sphoeroides spengleri* | 1 | -0.89 | -3.4 | 1.4 | -11.03 | -26.41 | -1.28 | 3.87 | 0.64 | 9.26 | 0.096 |
| *Montastraea cavernosa* | *Dactylopterus volitans* | 1 | -0.01 | -3.3 | 2.95 | -8.6 | -50.24 | 23.15 | 10.13 | -37.4 | 59.38 | 0.548 |
| *Montastraea cavernosa* | *Kyphosus sectatrix* | 1 | 0.24 | -2.64 | 3.2 | -23.63 | -58.65 | 4.68 | 1 | -50.04 | 56.55 | 0.579 |
| *Montastraea cavernosa* | *Aulostomus strigosus* | 1 | -0.18 | -3.63 | 2.88 | -13.63 | -50.17 | 13.99 | -8.1 | -61.01 | 47.82 | 0.611 |
| *Montastraea cavernosa* | *Caranx crysos* | 1 | 0.04 | -2.98 | 3.03 | -20.63 | -54.93 | 4.09 | -11.55 | -72.5 | 49.88 | 0.559 |
| *Montastraea cavernosa* | *Fistularia tabacaria* | 1 | 0.12 | -2.87 | 3.19 | -29.53 | -70.51 | 2.69 | 1.53 | -20.99 | 28.27 | 0.576 |
| *Montastraea cavernosa* | *Chaetodon sedentarius* | 1 | -2.77 | -4.9 | -1.36 | -4.84 | -13.7 | -0.13 | -0.03 | -0.84 | 0.86 | 0.607 |
| *Montastraea cavernosa* | *Priacanthus arenatus* | 1 | -1.79 | -5.52 | 1.73 | 3.34 | -20.89 | 25.08 | 1.04 | -11.09 | 11.81 | 0.45 |
| *Montastraea cavernosa* | *Bodianus pulchellus* | 1 | -2.49 | -4.62 | -1.11 | -5.21 | -14.28 | -0.28 | 0.22 | -0.54 | 1.03 | 0.088 |
| *Montastraea cavernosa* | *Elacatinus figaro* | 1 | -0.18 | -3.6 | 2.84 | -15.54 | -54.23 | 16.03 | -8.39 | -66.23 | 51.73 | 0.538 |
| *Montastraea cavernosa* | *Sphoeroides greeleyi* | 1 | -0.29 | -3.88 | 2.92 | -13.6 | -51.15 | 16.08 | -7.31 | -59.08 | 42.5 | 0.539 |
| *Montastraea cavernosa* | *Stephanolepis hispidus* | 1 | -2.21 | -4.44 | -0.82 | -5.52 | -14.46 | -0.4 | 0.23 | -0.5 | 1.03 | 0.011 |
| *Montastraea cavernosa* | *Epinephelus morio* | 1 | -0.52 | -3.64 | 2.8 | -18.07 | -59.29 | 10.26 | 10.51 | 0.09 | 34.34 | 0.228 |
| *Montastraea cavernosa* | *Heteropriacanthus cruentatus* | 1 | -0.28 | -3.61 | 2.82 | -3.71 | -48.17 | 34.81 | -27.7 | -68.54 | 5.18 | 0.456 |
| *Montastraea cavernosa* | *Labrisomus nuchipinnis* | 1 | -0.05 | -3.43 | 3.22 | -27.44 | -69.42 | 4.32 | 6.99 | -14.05 | 33.8 | 0.433 |
| *Montastraea cavernosa* | *Myrichthys ocellatus* | 1 | -0.35 | -3.78 | 2.65 | -3.82 | -48.92 | 35.95 | -26.81 | -68.78 | 11.95 | 0.458 |
| *Montastraea cavernosa* | *Acanthostracion quadricornis* | 1 | -0.58 | -4.17 | 2.6 | 4.62 | -38.65 | 39.61 | -21.71 | -62.55 | 1.87 | 0.386 |
| *Montastraea cavernosa* | *Epinephelus marginatus* | 1 | -2.14 | -4.91 | 0.87 | -3.18 | -14.84 | 12.23 | 6.97 | 1.23 | 22.14 | 0.114 |
| *Montastraea cavernosa* | *Mugil curema* | 1 | -0.66 | -4.48 | 2.59 | 10.54 | -35.08 | 52.16 | -20.27 | -56.61 | 0.78 | 0.376 |
| *Montastraea cavernosa* | *Orthopristis ruber* | 1 | -0.18 | -3.16 | 2.7 | -9.28 | -48.81 | 19.06 | 31.84 | 3.61 | 69.69 | 0.162 |
| *Montastraea cavernosa* | *Kyphosus vaigiensis* | 1 | -1.81 | -6.19 | 2.15 | 11.97 | -20.14 | 55.49 | 11 | -0.03 | 46.15 | 0.123 |
| *Montastraea cavernosa* | *Mycteroperca interstitialis* | 1 | -0.68 | -4.33 | 2.51 | 18.22 | -18.37 | 59.88 | 17.11 | 0.12 | 51.88 | 0.522 |
| *Montastraea cavernosa* | *Eucinostomus melanopterus* | 1 | -0.56 | -3.93 | 2.26 | 28.88 | -4.38 | 65.04 | 10.82 | 1.17 | 31.12 | 0.558 |
| *Montastraea cavernosa* | *Lutjanus cyanopterus* | 1 | -0.9 | -4.86 | 2.17 | 29.7 | -13.48 | 73.24 | 10.69 | 0.88 | 32.33 | 0.301 |
| *Montastraea cavernosa* | *Calamus penna* | 1 | -0.04 | -3.37 | 3.09 | -12.77 | -49.95 | 15.99 | -15.85 | -65.36 | 34.05 | 0.601 |
| *Montastraea cavernosa* | *Seriola rivoliana* | 1 | -0.16 | -3.7 | 2.85 | -13.48 | -51.36 | 14.59 | -13.08 | -63.22 | 44.05 | 0.603 |
| *Montastraea cavernosa* | *Parablennius pilicornis* | 1 | -0.3 | -3.68 | 2.71 | -0.68 | -52.42 | 43.89 | 21.53 | -3.64 | 59.96 | 0.698 |
| *Montastraea cavernosa* | *Pareques acuminatus* | 1 | -0.24 | -3.51 | 2.9 | 6.3 | -44.19 | 48.47 | 21.05 | -1.58 | 58.23 | 0.672 |
| *Montastraea cavernosa* | *Rhomboplites aurorubens* | 1 | -1.43 | -5.43 | 1.96 | 12.46 | -6.9 | 35.72 | 2.35 | -0.41 | 6.53 | 0.549 |
| *Montastraea cavernosa* | *Sphoeroides testudineus* | 1 | -0.17 | -3.24 | 2.67 | 0.24 | -47.07 | 40.69 | 26.29 | -5.98 | 65.85 | 0.805 |
| *Montastraea cavernosa* | *Myripristis jacobus* | 1 | -0.2 | -3.26 | 2.74 | 0.07 | -49.14 | 42.34 | 20.39 | -9.8 | 59.35 | 0.76 |
| *Montastraea cavernosa* | *Acanthurus bahianus* | 2 | 0.17 | -1.44 | 2.46 | 5.44 | -0.48 | 15.78 | 0.99 | -0.03 | 2.25 | 0.007 |
| *Montastraea cavernosa* | *Acanthurus chirurgus* | 2 | -2.45 | -4.1 | -1.25 | -3.27 | -9.26 | -0.17 | -1.27 | -2.22 | -0.45 | 0.554 |
| *Montastraea cavernosa* | *Sparisoma frondosum* | 2 | 0.49 | -1.27 | 3.07 | 6.06 | -0.33 | 17.23 | -1.18 | -3.32 | -0.01 | 0.419 |
| *Montastraea cavernosa* | *Sparisoma axillare* | 2 | 0.08 | -2.42 | 2.8 | 14.87 | 3.35 | 29.47 | -2.11 | -4.37 | -0.45 | 0.532 |
| *Montastraea cavernosa* | *Sparisoma radians* | 2 | -1.53 | -3.1 | -0.25 | -3.5 | -10.03 | 0.05 | -0.79 | -1.89 | 0.09 | 0.407 |
| *Montastraea cavernosa* | *Stegastes variabilis* | 2 | 1.2 | -1.31 | 4.44 | 12.86 | 2.08 | 27.91 | 0.54 | -0.67 | 1.9 | 0.652 |
| *Montastraea cavernosa* | *Abudefduf saxatilis* | 2 | 2.15 | 0.81 | 4.08 | 4.2 | 0.33 | 11.32 | 1.2 | 0.31 | 2.18 | 0.03 |
| *Montastraea cavernosa* | *Acanthurus coeruleus* | 2 | 0.12 | -1.67 | 2.34 | 12.39 | 4.27 | 23.64 | -1.3 | -2.86 | -0.09 | 0.059 |
| *Montastraea cavernosa* | *Chromis multilineata* | 2 | -0.08 | -3.01 | 2.88 | -28.61 | -69.63 | -1.82 | -0.84 | -8.71 | 4.52 | 0.024 |
| *Montastraea cavernosa* | *Halichoeres brasiliensis* | 2 | 0.28 | -2.67 | 3.1 | 20.63 | -0.7 | 53.14 | -7.3 | -32.26 | 1.86 | 0.163 |
| *Montastraea cavernosa* | *Halichoeres poeyi* | 2 | -0.25 | -0.89 | 0.42 | 0.05 | -1.12 | 1.08 | 0.26 | -0.37 | 0.91 | 0.004 |
| *Montastraea cavernosa* | *Holacanthus tricolor* | 2 | -0.19 | -3.19 | 2.72 | 2.93 | -45.89 | 42.52 | -29.67 | -66.83 | 1.26 | 0.787 |
| *Montastraea cavernosa* | *Microspathodon chrysurus* | 2 | -0.59 | -3.9 | 2.43 | 20.22 | -25.62 | 64.91 | -19.07 | -49.66 | -0.36 | 0.629 |
| *Montastraea cavernosa* | *Anisotremus virginicus* | 2 | 0.1 | -3.22 | 3.27 | -30.33 | -70.39 | 1.77 | 7.27 | -13 | 30.6 | 0.373 |
| *Montastraea cavernosa* | *Haemulon aurolineatum* | 2 | 0.39 | -1.52 | 2.63 | 7.83 | 0.22 | 18.41 | 2.19 | 0.64 | 4.2 | 0.319 |
| *Montastraea cavernosa* | *Stegastes fuscus* | 2 | -1 | -1.9 | -0.21 | -1.58 | -4.43 | 0.27 | 1.12 | 0.1 | 2.17 | 0.176 |
| *Montastraea cavernosa* | *Stegastes rocasensis* | 2 | -2.47 | -4.72 | -1 | -5.3 | -14.21 | -0.32 | -0.23 | -0.98 | 0.54 | 0.127 |
| *Montastraea cavernosa* | *Thalassoma noronhanum* | 2 | -2.64 | -4.77 | -1.27 | -5.03 | -13.87 | -0.27 | -0.25 | -0.99 | 0.52 | 0.213 |
| *Montastraea cavernosa* | *Halichoeres radiatus* | 2 | 0.8 | -0.92 | 3.17 | 7.69 | 0.47 | 17.17 | -0.31 | -1.31 | 0.63 | 0.154 |
| *Montastraea cavernosa* | *Cantherhines pullus* | 2 | -1.27 | -4.75 | 2.58 | -18.54 | -63.33 | 6.44 | 1.85 | -16.75 | 24.53 | 0.266 |
| *Montastraea cavernosa* | *Cryptotomus roseus* | 2 | -0.04 | -2.12 | 2.22 | 10.23 | 0.36 | 21.5 | 0.8 | -0.39 | 2.27 | 0.009 |
| *Montastraea cavernosa* | *Haemulon plumierii* | 2 | -0.68 | -3.61 | 2.08 | 43.38 | 15.11 | 79.43 | 2.78 | -2.07 | 9.83 | 0.083 |
| *Montastraea cavernosa* | *Canthigaster figueiredoi* | 2 | 0.4 | -1.88 | 3.13 | 6.14 | -22.9 | 21.7 | 3.1 | -1.09 | 19.3 | 0.508 |
| *Montastraea cavernosa* | *Chaetodon striatus* | 2 | -0.19 | -3.05 | 2.6 | 21 | -5.89 | 55.56 | 14.39 | 0.1 | 48.97 | 0.257 |
| *Montastraea cavernosa* | *Mycteroperca bonaci* | 2 | -0.62 | -3.64 | 2 | 42.75 | 13.93 | 80.95 | 3.4 | -2.56 | 11.71 | 0.428 |
| *Montastraea cavernosa* | *Pomacanthus paru* | 2 | 0.22 | -1.84 | 2.7 | 12.01 | 1.82 | 24.04 | 2.88 | -0.51 | 11.08 | 0.302 |
| *Montastraea cavernosa* | *Scarus zelindae* | 2 | 0.1 | -1.61 | 2.09 | 10.31 | 2.7 | 19.53 | 0.54 | -0.61 | 1.87 | 0.135 |
| *Montastraea cavernosa* | *Sphoeroides spengleri* | 2 | 0.32 | -1.09 | 2.33 | 3.82 | -0.67 | 12.57 | 1.62 | 0.3 | 3.84 | 0.067 |
| *Montastraea cavernosa* | *Ocyurus chrysurus* | 2 | -0.73 | -4.02 | 2.13 | 42.64 | 11.63 | 80.04 | 3.3 | -2.93 | 11.64 | 0.338 |
| *Montastraea cavernosa* | *Labrisomus nuchipinnis* | 2 | -0.24 | -3.39 | 2.65 | 11.44 | -35.34 | 52.96 | 24.6 | 0.59 | 63.43 | 0.683 |
| *Montastraea cavernosa* | *Bodianus pulchellus* | 2 | 0.05 | -2.98 | 2.97 | -16.14 | -52.45 | 11.58 | 24.11 | -3.12 | 64.47 | 0.632 |
| *Montastraea cavernosa* | *Chilomycterus spinosus spinosus* | 2 | -0.42 | -4.34 | 2.75 | -12.86 | -51.71 | 15.8 | -2.84 | -58.15 | 50.94 | 0.528 |
| *Montastraea cavernosa* | *Holocentrus adscensionis* | 2 | -0.06 | -3.12 | 3.04 | -17.38 | -53.82 | 7.13 | -28.19 | -68.13 | 6.12 | 0.322 |
| *Montastraea cavernosa* | *Pseudupeneus maculatus* | 2 | -2.64 | -4.86 | -0.69 | -5.46 | -16.61 | 0.35 | 0.16 | -0.8 | 1.57 | 0.435 |
| *Montastraea cavernosa* | *Serranus baldwini* | 2 | -1.92 | -4.22 | 0.96 | -10.2 | -47.44 | -0.42 | 0.03 | -4.81 | 7.54 | 0.506 |
| *Montastraea cavernosa* | *Stephanolepis hispidus* | 2 | -3 | -5.11 | -1.6 | -4.52 | -12.55 | -0.05 | -0.02 | -0.88 | 0.93 | 0.426 |
| *Montastraea cavernosa* | *Bodianus rufus* | 2 | -2.53 | -5.1 | 0.87 | -1.53 | -14.45 | 24.91 | 7 | -0.42 | 42.25 | 0.494 |
| *Montastraea cavernosa* | *Diplodus argenteus* | 2 | 0.11 | -2.83 | 3.2 | -31.43 | -69.43 | -1.03 | 9.21 | -15.4 | 34.66 | 0.46 |
| *Montastraea cavernosa* | *Halichoeres dimidiatus* | 2 | -1.45 | -4.88 | 2.05 | -2.32 | -33.88 | 29.68 | -20.38 | -62.83 | 0.87 | 0.191 |
| *Montastraea cavernosa* | *Sparisoma tuiupiranga* | 2 | -0.23 | -3.28 | 2.72 | 0.89 | -38.49 | 38.1 | -33.83 | -71.52 | -3.4 | 0.182 |
| *Montastraea cavernosa* | *Eucinostomus melanopterus* | 2 | -0.67 | -4.39 | 2.28 | 31.69 | -0.53 | 65.67 | 6.92 | 1.13 | 14.6 | 0.495 |
| *Montastraea cavernosa* | *Sphoeroides greeleyi* | 2 | -0.25 | -3.4 | 2.61 | 11.6 | -39.18 | 55.9 | 24.4 | 0.71 | 60.35 | 0.682 |
| *Montastraea cavernosa* | *Sphoeroides testudineus* | 2 | -0.31 | -3.68 | 2.67 | 11.32 | -38.5 | 56.17 | 25.82 | 0.73 | 63.64 | 0.687 |
| *Montastraea cavernosa* | *Coryphopterus glaucofraenum* | 2 | -0.3 | -3.32 | 2.68 | 6.72 | -45.4 | 51.45 | 24.19 | 0.5 | 58.32 | 0.608 |
| *Montastraea cavernosa* | *Synodus synodus* | 2 | -0.42 | -3.65 | 2.47 | 0.58 | -47.43 | 43.82 | 20.57 | -4.41 | 58.99 | 0.696 |
| *Montastraea cavernosa* | *Pareques acuminatus* | 2 | -0.26 | -3.42 | 2.8 | -0.45 | -51.66 | 42.13 | 23.43 | -17.06 | 66.12 | 0.78 |
| *Montastraea cavernosa* | *Sparisoma amplum* | 2 | -0.2 | -3.35 | 2.73 | -3.51 | -51.68 | 38.23 | 23.11 | -8.27 | 62.74 | 0.757 |
| *Mussismilia harttii* | *Acanthurus chirurgus* | 1 | 0.87 | 0.24 | 1.58 | -0.41 | -1.11 | 0.25 | -0.49 | -1.36 | 0.27 | 0 |
| *Mussismilia harttii* | *Chaetodon ocellatus* | 1 | -2.92 | -5.08 | -1.42 | -3.5 | -9.64 | -0.07 | -0.82 | -1.69 | -0.02 | 0.614 |
| *Mussismilia harttii* | *Haemulon plumierii* | 1 | -0.22 | -1.24 | 1.85 | 1.22 | -0.02 | 4.06 | -1.13 | -4.97 | 0.07 | 0.047 |
| *Mussismilia harttii* | *Halichoeres dimidiatus* | 1 | -1.47 | -3.96 | 1.35 | -6.15 | -23.82 | 2.55 | -3.72 | -18.08 | 0.09 | 0.094 |
| *Mussismilia harttii* | *Ocyurus chrysurus* | 1 | -0.5 | -1.57 | 1.52 | 2.06 | -0.06 | 9.01 | -0.2 | -0.94 | 0.57 | 0.003 |
| *Mussismilia harttii* | *Pomacanthus arcuatus* | 1 | -2.58 | -4.05 | -1.38 | -1.64 | -5.2 | 0.3 | 0.48 | -0.55 | 1.7 | 0.33 |
| *Mussismilia harttii* | *Pseudupeneus maculatus* | 1 | 1.72 | 0.65 | 3.69 | 0.37 | -0.72 | 2.33 | -0.85 | -2.73 | 0.29 | 0.01 |
| *Mussismilia harttii* | *Sparisoma axillare* | 1 | 2.66 | 1.23 | 4.88 | 4.06 | 0.31 | 10.66 | -0.92 | -2 | -0.03 | 0.001 |
| *Mussismilia harttii* | *Sparisoma frondosum* | 1 | 2.29 | 0.9 | 4.48 | 4.28 | 0.36 | 10.82 | 0.03 | -0.69 | 0.71 | 0.029 |
| *Mussismilia harttii* | *Thalassoma noronhanum* | 1 | -1 | -1.68 | -0.37 | 0.09 | -0.65 | 0.73 | -0.62 | -1.34 | 0.03 | 0 |
| *Mussismilia harttii* | *Abudefduf saxatilis* | 1 | 5.02 | 2.69 | 8.41 | 2.55 | -0.75 | 8.93 | -0.23 | -2.58 | 1.55 | 0 |
| *Mussismilia harttii* | *Acanthurus bahianus* | 1 | 2.45 | 1.05 | 4.69 | 4.22 | 0.34 | 11.04 | 0.14 | -0.54 | 0.82 | 0.014 |
| *Mussismilia harttii* | *Acanthurus coeruleus* | 1 | 1.89 | 0.34 | 4.13 | 5.92 | 1.17 | 12.96 | -1.06 | -2.01 | -0.27 | 0.071 |
| *Mussismilia harttii* | *Anisotremus virginicus* | 1 | 2.37 | 1.04 | 4.92 | 1.5 | -0.41 | 5.81 | -0.17 | -1.93 | 1.25 | 0.008 |
| *Mussismilia harttii* | *Bodianus rufus* | 1 | 1.14 | -0.07 | 3.3 | 2.4 | -0.15 | 9.04 | 0.44 | -0.31 | 1.25 | 0.011 |
| *Mussismilia harttii* | *Carangoides bartholomaei* | 1 | -0.43 | -1.25 | 0.45 | 0.13 | -0.7 | 0.87 | -0.21 | -0.94 | 0.49 | 0.023 |
| *Mussismilia harttii* | *Caranx ruber* | 1 | -1.13 | -4.56 | 2.52 | -10.56 | -54.91 | 13.91 | -5.83 | -27.43 | 0.6 | 0.349 |
| *Mussismilia harttii* | *Haemulon aurolineatum* | 1 | 0.91 | 0.18 | 1.88 | 0.43 | -0.61 | 2.72 | 0.18 | -0.48 | 0.84 | 0 |
| *Mussismilia harttii* | *Haemulon parra* | 1 | 0.56 | -1.02 | 2.62 | 6.86 | 1.51 | 13.67 | -1.25 | -2.34 | -0.33 | 0.544 |
| *Mussismilia harttii* | *Halichoeres bivittatus* | 1 | -0.25 | -3.29 | 2.73 | 11 | -23.18 | 42.54 | -31.72 | -64.14 | -4.99 | 0.298 |
| *Mussismilia harttii* | *Halichoeres brasiliensis* | 1 | 1.85 | 0.41 | 4.06 | 5.03 | 0.81 | 11.92 | 0.68 | 0.02 | 1.43 | 0.001 |
| *Mussismilia harttii* | *Halichoeres poeyi* | 1 | 1.37 | 0.47 | 2.65 | -0.36 | -1.27 | 0.5 | 0.47 | -0.34 | 1.39 | 0.007 |
| *Mussismilia harttii* | *Holacanthus ciliaris* | 1 | -0.01 | -1.31 | 2.22 | 0.81 | -1.82 | 7.95 | -1.44 | -4.39 | 0 | 0.098 |
| *Mussismilia harttii* | *Holocentrus adscensionis* | 1 | 1.37 | 0.61 | 2.33 | -0.03 | -0.75 | 0.78 | 0.28 | -0.5 | 1.09 | 0 |
| *Mussismilia harttii* | *Lutjanus jocu* | 1 | -0.49 | -1.47 | 0.92 | -0.59 | -2.06 | 0.41 | -0.96 | -3.01 | 0.07 | 0.062 |
| *Mussismilia harttii* | *Pomacanthus paru* | 1 | 0.18 | -0.53 | 0.88 | -0.7 | -1.75 | 0.1 | -0.07 | -0.77 | 0.61 | 0.004 |
| *Mussismilia harttii* | *Scarus trispinosus* | 1 | -0.94 | -2.03 | 0.69 | -0.26 | -1.83 | 0.67 | -1.45 | -5.46 | -0.07 | 0.005 |
| *Mussismilia harttii* | *Scarus zelindae* | 1 | 0.24 | -0.74 | 1.8 | 2.79 | 0.47 | 7.81 | -0.2 | -0.9 | 0.51 | 0.001 |
| *Mussismilia harttii* | *Sparisoma amplum* | 1 | -0.37 | -1.78 | 1.9 | 2.79 | 0.26 | 9.53 | 0.23 | -0.72 | 1.24 | 0.165 |
| *Mussismilia harttii* | *Sparisoma radians* | 1 | 1.33 | -0.61 | 4.08 | 4.27 | 0.33 | 11.55 | -1.04 | -3.15 | 0.63 | 0.724 |
| *Mussismilia harttii* | *Sphyraena barracuda* | 1 | -0.92 | -4.48 | 2.32 | 11.51 | -4.89 | 37.34 | -11.95 | -52.82 | -0.31 | 0.239 |
| *Mussismilia harttii* | *Stegastes variabilis* | 1 | 0.87 | -1.18 | 3.48 | 6.47 | 0.31 | 15.32 | 0.92 | -0.37 | 2.18 | 0.339 |
| *Mussismilia harttii* | *Chaetodon striatus* | 1 | 3.07 | 1.15 | 6.11 | 1.65 | -1.09 | 8.23 | 1.1 | -0.31 | 2.65 | 0 |
| *Mussismilia harttii* | *Lutjanus apodus* | 1 | -0.35 | -3.45 | 2.63 | 15.68 | -36.96 | 65.78 | -20.95 | -58.72 | -1.9 | 0.688 |
| *Mussismilia harttii* | *Stegastes fuscus* | 1 | 2.18 | 0.77 | 4.34 | 4.35 | 0.48 | 10.84 | 0.67 | 0.02 | 1.37 | 0.02 |
| *Mussismilia harttii* | *Acanthostracion polygonius* | 1 | -0.02 | -2.84 | 2.7 | -18.51 | -50.51 | 2.27 | -31.18 | -65.95 | -2.03 | 0.401 |
| *Mussismilia harttii* | *Amblycirrhitus pinos* | 1 | -0.53 | -4.23 | 2.66 | 10.15 | -19.35 | 41.66 | -24.47 | -65.47 | 1.21 | 0.485 |
| *Mussismilia harttii* | *Anisotremus surinamensis* | 1 | 0.14 | -2.79 | 3.21 | -34.62 | -75.77 | -1.93 | 3.91 | -8.5 | 16.18 | 0.341 |
| *Mussismilia harttii* | *Aulostomus maculatus* | 1 | -0.15 | -3.16 | 2.68 | 5.28 | -38.26 | 42.55 | -32.82 | -69.32 | -1.26 | 0.731 |
| *Mussismilia harttii* | *Cantherhines pullus* | 1 | -2.28 | -4.47 | -0.93 | -4.08 | -10.73 | -0.39 | -0.18 | -0.92 | 0.52 | 0.112 |
| *Mussismilia harttii* | *Canthidermis maculata* | 1 | -1.77 | -2.85 | -0.7 | 0.44 | -0.86 | 1.14 | -0.17 | -1.08 | 0.79 | 0.215 |
| *Mussismilia harttii* | *Cephalopholis fulva* | 1 | -1.35 | -2.21 | -0.54 | 1.37 | 0.38 | 2.67 | -0.24 | -1.05 | 0.57 | 0.224 |
| *Mussismilia harttii* | *Chromis multilineata* | 1 | 0.95 | -0.29 | 2.98 | 1.07 | -0.92 | 6.62 | 1.06 | 0.05 | 2.4 | 0.004 |
| *Mussismilia harttii* | *Clepticus brasiliensis* | 1 | -0.56 | -3.52 | 2.51 | -9.36 | -52.69 | 8.94 | 0.49 | -5.23 | 9.93 | 0.411 |
| *Mussismilia harttii* | *Epinephelus adscensionis* | 1 | -0.29 | -2.37 | 2.4 | 7.26 | 0.8 | 17.67 | -0.72 | -5.34 | 1.33 | 0.433 |
| *Mussismilia harttii* | *Haemulon squamipinna* | 1 | 0.05 | -2.72 | 2.81 | 19.69 | 4.08 | 43.3 | -38.24 | -69.95 | -10.17 | 0.06 |
| *Mussismilia harttii* | *Halichoeres penrosei* | 1 | -0.31 | -3.28 | 2.53 | -14.38 | -39.41 | 0.85 | -31.27 | -63.65 | -5.28 | 0.051 |
| *Mussismilia harttii* | *Microspathodon chrysurus* | 1 | -0.04 | -2.17 | 2.8 | 10.42 | 0.91 | 29.05 | -2.77 | -15.61 | 0.77 | 0.306 |
| *Mussismilia harttii* | *Ophioblennius macclurei* | 1 | -0.09 | -3.21 | 2.79 | -2.98 | -52.71 | 37.76 | -31.06 | -73.41 | 10.28 | 0.805 |
| *Mussismilia harttii* | *Scomberomorus regalis* | 1 | -0.14 | -3.2 | 2.82 | -1.41 | -46.9 | 39.3 | -30.12 | -69.47 | 5.22 | 0.798 |
| *Mussismilia harttii* | *Serranus baldwini* | 1 | -1.52 | -4.17 | 1.64 | -15.21 | -60.76 | -0.63 | 3.91 | -0.69 | 21.49 | 0.01 |
| *Mussismilia harttii* | *Halichoeres radiatus* | 1 | -1.03 | -1.86 | -0.28 | -1.23 | -3.25 | 0.04 | -0.26 | -0.95 | 0.41 | 0.002 |
| *Mussismilia harttii* | *Holacanthus tricolor* | 1 | -0.84 | -3.87 | 2.37 | -4.44 | -30.3 | 10.19 | -7.3 | -40.17 | 0.78 | 0.325 |
| *Mussismilia harttii* | *Lutjanus analis* | 1 | -0.27 | -3.39 | 2.64 | 16.18 | -32.03 | 61.85 | -26.09 | -61.21 | -3.35 | 0.74 |
| *Mussismilia harttii* | *Mycteroperca bonaci* | 1 | 0.83 | -1.66 | 4.11 | 3.45 | -3.75 | 13.93 | -0.91 | -6.54 | 1.84 | 0.334 |
| *Mussismilia harttii* | *Pseudocaranx dentex* | 1 | -0.01 | -3.07 | 3.14 | -26.7 | -60.01 | -1.25 | 22.93 | 2.43 | 51.95 | 0.506 |
| *Mussismilia harttii* | *Bothus ocellatus* | 1 | -0.55 | -4.32 | 2.53 | 13.67 | -35.3 | 59.15 | 18.92 | 0.08 | 56.16 | 0.534 |
| *Mussismilia harttii* | *Coryphopterus glaucofraenum* | 1 | -0.04 | -2.85 | 2.94 | -35.03 | -77.31 | -3.26 | -2.23 | -12.37 | 5.22 | 0.533 |
| *Mussismilia harttii* | *Malacanthus plumieri* | 1 | -2.75 | -5.03 | -1.32 | -3.62 | -10.12 | -0.08 | 0.4 | -0.46 | 1.35 | 0.693 |
| *Mussismilia harttii* | *Stegastes rocasensis* | 1 | -2.61 | -4.71 | -1.29 | -3.7 | -10.14 | -0.16 | -0.17 | -0.92 | 0.58 | 0.443 |
| *Mussismilia harttii* | *Apogon americanus* | 1 | -0.13 | -3.33 | 2.92 | -19.85 | -55.25 | 8.22 | 0.28 | -52.6 | 59.91 | 0.695 |
| *Mussismilia harttii* | *Aluterus scriptus* | 1 | -0.75 | -3.94 | 2.59 | -26.57 | -74.81 | -0.77 | 0.66 | -10.42 | 10.28 | 0.283 |
| *Mussismilia harttii* | *Melichthys niger* | 1 | -3.61 | -5.83 | -2.09 | -2.97 | -9.43 | 0.3 | -0.31 | -1.33 | 0.76 | 0.4 |
| *Mussismilia harttii* | *Rypticus saponaceus* | 1 | -0.05 | -2.82 | 2.79 | 15.53 | -0.58 | 40.5 | 26.15 | 0.16 | 64.7 | 0.742 |
| *Mussismilia harttii* | *Mulloidichthys martinicus* | 1 | -0.15 | -2.45 | 2.49 | 7.54 | 0.61 | 17.16 | 0.04 | -1.54 | 1.66 | 0.498 |
| *Mussismilia harttii* | *Caranx latus* | 1 | 0.08 | -2.51 | 2.73 | 40.98 | 14.34 | 75.38 | 10.56 | 2.41 | 21.04 | 0.263 |
| *Mussismilia harttii* | *Diodon hystrix* | 1 | -0.03 | -2.97 | 2.89 | -17.14 | -53.59 | 7.02 | -26.04 | -68.36 | 14.39 | 0.443 |
| *Mussismilia harttii* | *Cryptotomus roseus* | 1 | -0.5 | -1.33 | 0.76 | 1.42 | 0.07 | 5.11 | 0.28 | -0.45 | 1.07 | 0.001 |
| *Mussismilia harttii* | *Sparisoma tuiupiranga* | 1 | -0.89 | -1.56 | -0.22 | -0.16 | -1.18 | 0.56 | -0.08 | -0.74 | 0.59 | 0.111 |
| *Mussismilia harttii* | *Odontoscion dentex* | 1 | -2.24 | -5.29 | 1.25 | -1.1 | -18.24 | 26.01 | 14.16 | 0.75 | 55.53 | 0.38 |
| *Mussismilia harttii* | *Aluterus monoceros* | 1 | -0.33 | -3.77 | 2.65 | 0.37 | -49.28 | 45.6 | 24.6 | -1.52 | 64.18 | 0.654 |
| *Mussismilia harttii* | *Haemulon steindachneri* | 1 | -0.55 | -3.84 | 2.39 | 16.88 | -6.57 | 43.35 | 35.88 | 10.4 | 70.34 | 0.494 |
| *Mussismilia harttii* | *Lactophrys trigonus* | 1 | -0.33 | -3.67 | 2.73 | -3.55 | -50.98 | 40.66 | 21.89 | -4.49 | 62.11 | 0.682 |
| *Mussismilia harttii* | *Mycteroperca acutirostris* | 1 | -1.3 | -2.98 | 0.33 | -4.52 | -11.25 | -0.22 | 4.13 | 1.29 | 9.27 | 0.347 |
| *Mussismilia harttii* | *Synodus intermedius* | 1 | -0.17 | -3.09 | 2.69 | 20.02 | 1.28 | 53.85 | 25.17 | 0.85 | 62.03 | 0.651 |
| *Mussismilia harttii* | *Balistes vetula* | 1 | -0.16 | -3.49 | 3.34 | -20.68 | -65.7 | 13.58 | 8.92 | -8.67 | 35.56 | 0.282 |
| *Mussismilia harttii* | *Canthigaster figueiredoi* | 1 | -0.18 | -2.23 | 2.07 | -20.37 | -57.48 | -2.41 | 8.35 | 1.01 | 21.32 | 0 |
| *Mussismilia harttii* | *Malacoctenus delalandii* | 1 | 0.37 | -2.19 | 3.05 | 40.23 | -2.33 | 80.48 | 8.05 | -2.28 | 26.26 | 0.341 |
| *Mussismilia harttii* | *Anisotremus moricandi* | 1 | -1.17 | -5.17 | 2.35 | -4.17 | -38.21 | 17.85 | -4.34 | -51.48 | 34.04 | 0.44 |
| *Mussismilia harttii* | *Chilomycterus spinosus spinosus* | 1 | 0.01 | -3.01 | 3.04 | -29.37 | -66.22 | -2.32 | -9.86 | -33.61 | 14.31 | 0.129 |
| *Mussismilia harttii* | *Diplodus argenteus* | 1 | -1.95 | -4.16 | -0.38 | -6.15 | -13.17 | -1.47 | 1.53 | 0.62 | 2.63 | 0.283 |
| *Mussismilia harttii* | *Sphoeroides spengleri* | 1 | -1.39 | -3.96 | 0.83 | -10.9 | -23.21 | -2.45 | 4.21 | 1.15 | 9.04 | 0.123 |
| *Mussismilia harttii* | *Dactylopterus volitans* | 1 | -0.26 | -3.79 | 2.82 | -15.25 | -53.55 | 13.64 | 1.95 | -52.43 | 58.89 | 0.552 |
| *Mussismilia harttii* | *Kyphosus sectatrix* | 1 | 0.1 | -2.79 | 3.06 | -21.83 | -53.61 | 2.22 | -4.32 | -53.33 | 52.38 | 0.57 |
| *Mussismilia harttii* | *Aulostomus strigosus* | 1 | -0.21 | -3.68 | 2.85 | -14.96 | -50.26 | 11.77 | -4.99 | -57.47 | 46.85 | 0.608 |
| *Mussismilia harttii* | *Caranx crysos* | 1 | 0.04 | -2.96 | 2.98 | -21.32 | -52.48 | 0.55 | -0.18 | -56.41 | 60.07 | 0.562 |
| *Mussismilia harttii* | *Fistularia tabacaria* | 1 | 0.04 | -3.06 | 3.17 | -32.35 | -70 | -2.95 | 4.9 | -25.83 | 36.56 | 0.643 |
| *Mussismilia harttii* | *Chaetodon sedentarius* | 1 | -2.76 | -4.91 | -1.36 | -3.71 | -10.08 | -0.12 | 0.01 | -0.78 | 0.88 | 0.592 |
| *Mussismilia harttii* | *Priacanthus arenatus* | 1 | -1.44 | -5.42 | 2.21 | 0.46 | -32.32 | 19.95 | -4.11 | -40.49 | 3.62 | 0.459 |
| *Mussismilia harttii* | *Bodianus pulchellus* | 1 | -2.46 | -4.62 | -1.11 | -3.95 | -10.24 | -0.29 | 0.29 | -0.43 | 1.12 | 0.09 |
| *Mussismilia harttii* | *Elacatinus figaro* | 1 | -0.32 | -4 | 2.88 | -14.68 | -52.16 | 11.89 | -5.81 | -55.54 | 47.39 | 0.526 |
| *Mussismilia harttii* | *Sphoeroides greeleyi* | 1 | -0.08 | -3.4 | 3 | -16.25 | -53.63 | 11.23 | -1.58 | -54.72 | 57.29 | 0.549 |
| *Mussismilia harttii* | *Stephanolepis hispidus* | 1 | -2.24 | -4.47 | -0.83 | -4.44 | -11.25 | -0.51 | 0.32 | -0.38 | 1.07 | 0.008 |
| *Mussismilia harttii* | *Epinephelus morio* | 1 | -0.32 | -3.64 | 3.01 | -22.01 | -57.65 | 1.45 | 15.39 | 0.36 | 42.25 | 0.247 |
| *Mussismilia harttii* | *Heteropriacanthus cruentatus* | 1 | -0.33 | -3.85 | 2.73 | -10.47 | -51.66 | 24.69 | -24.47 | -67.06 | 20.61 | 0.484 |
| *Mussismilia harttii* | *Labrisomus nuchipinnis* | 1 | 0.04 | -3.05 | 3.17 | -31 | -68.52 | -1.33 | 7.93 | -19.94 | 39.02 | 0.462 |
| *Mussismilia harttii* | *Myrichthys ocellatus* | 1 | -0.14 | -3.24 | 2.86 | -9.77 | -52.1 | 27.99 | -27.94 | -70.36 | 16.46 | 0.481 |
| *Mussismilia harttii* | *Acanthostracion quadricornis* | 1 | -0.47 | -4.35 | 2.78 | -2.39 | -43.42 | 29.85 | -17.9 | -57.04 | 13.61 | 0.417 |
| *Mussismilia harttii* | *Epinephelus marginatus* | 1 | -2.73 | -5.53 | -0.35 | -5.51 | -14.43 | -0.08 | 6.84 | 1.35 | 23.02 | 0.108 |
| *Mussismilia harttii* | *Mugil curema* | 1 | -0.47 | -4 | 2.64 | 7.93 | -41.28 | 50.89 | -24.26 | -65.03 | 0.62 | 0.406 |
| *Mussismilia harttii* | *Orthopristis ruber* | 1 | -0.22 | -3.17 | 2.6 | 27.01 | 2.82 | 59.48 | 25.64 | 5.64 | 56.64 | 0.113 |
| *Mussismilia harttii* | *Kyphosus vaigiensis* | 1 | -2.7 | -6.66 | 1.94 | 4.36 | -20.27 | 42.9 | 9.08 | -0.2 | 41.94 | 0.15 |
| *Mussismilia harttii* | *Mycteroperca interstitialis* | 1 | -0.48 | -4.18 | 2.66 | 13.62 | -24.73 | 49.24 | 21.37 | 0.11 | 62.9 | 0.533 |
| *Mussismilia harttii* | *Eucinostomus melanopterus* | 1 | -0.43 | -3.78 | 2.54 | 27.98 | -13.65 | 68.37 | 16.52 | 1.73 | 44.64 | 0.588 |
| *Mussismilia harttii* | *Lutjanus cyanopterus* | 1 | -1.17 | -5.7 | 2.27 | 16.57 | -31.74 | 66.12 | 14.16 | 0.53 | 44.13 | 0.342 |
| *Mussismilia harttii* | *Calamus penna* | 1 | -0.15 | -3.55 | 2.86 | -15.5 | -51.84 | 9.98 | -12.21 | -62.67 | 40.39 | 0.609 |
| *Mussismilia harttii* | *Seriola rivoliana* | 1 | -0.11 | -3.39 | 2.98 | -16.88 | -53.89 | 10.04 | -10.88 | -62.84 | 41.46 | 0.633 |
| *Mussismilia harttii* | *Parablennius pilicornis* | 1 | -0.14 | -3.42 | 2.99 | -9.5 | -53.8 | 34.31 | 20.75 | -16.75 | 64.32 | 0.722 |
| *Mussismilia harttii* | *Pareques acuminatus* | 1 | -0.33 | -3.68 | 2.76 | -5.7 | -52.56 | 37.48 | 21.75 | -14.11 | 62.98 | 0.723 |
| *Mussismilia harttii* | *Rhomboplites aurorubens* | 1 | -1.49 | -5.62 | 2.08 | 9.12 | -5.6 | 27.05 | 2.4 | -0.54 | 6.85 | 0.565 |
| *Mussismilia harttii* | *Sphoeroides testudineus* | 1 | -0.12 | -3.22 | 2.85 | -3.88 | -49.69 | 32.83 | 25.5 | -11.15 | 65.24 | 0.779 |
| *Mussismilia harttii* | *Myripristis jacobus* | 1 | -0.53 | -3.72 | 2.31 | 41.93 | 11.98 | 80.94 | 1.64 | -9.12 | 13.75 | 0.556 |
| *Mussismilia harttii* | *Acanthurus bahianus* | 2 | -0.44 | -1.73 | 2.07 | 1.97 | -0.36 | 10.07 | 0.95 | -0.05 | 2.14 | 0.009 |
| *Mussismilia harttii* | *Acanthurus chirurgus* | 2 | -2.79 | -4.96 | -1.34 | -3.64 | -9.95 | -0.21 | -1.18 | -2.11 | -0.38 | 0.569 |
| *Mussismilia harttii* | *Sparisoma frondosum* | 2 | 1.91 | -0.32 | 4.85 | 10.69 | 3.43 | 19.62 | -2.98 | -9.16 | -0.53 | 0.325 |
| *Mussismilia harttii* | *Sparisoma axillare* | 2 | 0.53 | -2.78 | 3.86 | 4.52 | -23.89 | 22.43 | -2.56 | -9.71 | -0.16 | 0.421 |
| *Mussismilia harttii* | *Sparisoma radians* | 2 | 0.42 | -1.22 | 2.65 | 7.51 | 1.51 | 15.02 | -1.48 | -3.04 | -0.34 | 0.267 |
| *Mussismilia harttii* | *Stegastes variabilis* | 2 | 1.2 | -0.72 | 3.64 | 8.54 | 1.22 | 17.01 | -0.27 | -1.67 | 1.01 | 0.452 |
| *Mussismilia harttii* | *Abudefduf saxatilis* | 2 | 2.35 | 0.88 | 4.58 | 4.05 | 0.44 | 10.44 | 1.12 | 0.29 | 2.04 | 0.03 |
| *Mussismilia harttii* | *Acanthurus coeruleus* | 2 | -1.36 | -2.37 | -0.33 | -0.16 | -1.21 | 0.64 | -0.96 | -2.04 | -0.05 | 0.089 |
| *Mussismilia harttii* | *Chromis multilineata* | 2 | 1.06 | -0.77 | 3.57 | 3.89 | -0.21 | 10.76 | 0.02 | -1.37 | 1.23 | 0.025 |
| *Mussismilia harttii* | *Halichoeres brasiliensis* | 2 | -1.94 | -5 | 2.02 | -14.64 | -59.09 | 0.06 | 1.38 | -15.85 | 22.7 | 0.438 |
| *Mussismilia harttii* | *Halichoeres poeyi* | 2 | -0.06 | -0.83 | 0.97 | 1.35 | 0.05 | 4.48 | 0.28 | -0.36 | 0.96 | 0.005 |
| *Mussismilia harttii* | *Holacanthus tricolor* | 2 | -0.15 | -3.12 | 2.85 | 0.19 | -46.5 | 39.07 | -29.11 | -67.74 | 3.85 | 0.791 |
| *Mussismilia harttii* | *Microspathodon chrysurus* | 2 | -0.42 | -3.68 | 2.47 | 19.84 | -25.49 | 62.16 | -24.18 | -60.83 | -1.11 | 0.655 |
| *Mussismilia harttii* | *Anisotremus virginicus* | 2 | 0.07 | -2.95 | 3.13 | -34.46 | -74.51 | -2.06 | 3.83 | -24.67 | 33.71 | 0.374 |
| *Mussismilia harttii* | *Haemulon aurolineatum* | 2 | -0.85 | -3.13 | 2.04 | -14.02 | -50.29 | -0.2 | 12.28 | 0.72 | 43.35 | 0.211 |
| *Mussismilia harttii* | *Stegastes fuscus* | 2 | -0.79 | -1.6 | 0.01 | 1.23 | 0.22 | 2.55 | 1.18 | 0.27 | 2.29 | 0.192 |
| *Mussismilia harttii* | *Stegastes rocasensis* | 2 | -2.41 | -4.61 | -0.98 | -4.08 | -10.7 | -0.23 | -0.17 | -0.92 | 0.58 | 0.126 |
| *Mussismilia harttii* | *Thalassoma noronhanum* | 2 | -2.63 | -4.74 | -1.23 | -3.79 | -10.24 | -0.19 | -0.17 | -0.89 | 0.56 | 0.209 |
| *Mussismilia harttii* | *Halichoeres radiatus* | 2 | 0.58 | -1.46 | 4.06 | -1.78 | -8.47 | 4.43 | 0.23 | -1.38 | 2.3 | 0.239 |
| *Mussismilia harttii* | *Cantherhines pullus* | 2 | -0.94 | -4.68 | 2.78 | -23.02 | -65.38 | 0.39 | 1.56 | -24.96 | 28.81 | 0.257 |
| *Mussismilia harttii* | *Cryptotomus roseus* | 2 | -2.13 | -3.53 | -1.02 | -2.2 | -6.17 | 0.14 | 0.79 | -0.33 | 1.9 | 0.028 |
| *Mussismilia harttii* | *Haemulon plumierii* | 2 | -3.3 | -6.44 | 1.02 | -2.59 | -21.4 | 19.56 | 6.28 | -0.8 | 43.03 | 0.358 |
| *Mussismilia harttii* | *Canthigaster figueiredoi* | 2 | 0.12 | -2.79 | 3.12 | -35.18 | -72.44 | -4.03 | 6.08 | -22.14 | 34.64 | 0.372 |
| *Mussismilia harttii* | *Chaetodon striatus* | 2 | -0.63 | -4.09 | 2.69 | -2.25 | -52.12 | 43.13 | 21.93 | -8.29 | 61.97 | 0.37 |
| *Mussismilia harttii* | *Mycteroperca bonaci* | 2 | -0.39 | -4.01 | 2.66 | -2.55 | -50.35 | 39.33 | 20.62 | -9.05 | 59.79 | 0.661 |
| *Mussismilia harttii* | *Pomacanthus paru* | 2 | -0.01 | -2.81 | 2.77 | -21.45 | -57.6 | 0.15 | 30.91 | 3.49 | 68.76 | 0.34 |
| *Mussismilia harttii* | *Scarus zelindae* | 2 | 0.52 | -2.14 | 3.35 | 10.4 | 1.29 | 20.61 | 0.68 | -0.77 | 2.53 | 0.106 |
| *Mussismilia harttii* | *Sphoeroides spengleri* | 2 | -1.19 | -2.75 | 0.08 | -4.41 | -10.4 | -0.58 | 2.17 | 0.39 | 5.22 | 0.067 |
| *Mussismilia harttii* | *Ocyurus chrysurus* | 2 | 0.4 | -2.3 | 3.19 | 38.45 | -4.81 | 77.7 | 9.23 | -2.33 | 39.43 | 0.351 |
| *Mussismilia harttii* | *Labrisomus nuchipinnis* | 2 | -0.29 | -3.47 | 2.58 | 6.96 | -42.14 | 46.97 | 23.46 | -3.22 | 58.91 | 0.692 |
| *Mussismilia harttii* | *Bodianus pulchellus* | 2 | 0.03 | -2.96 | 3.03 | -19.28 | -62.11 | 7.12 | 24.77 | -10.16 | 65.95 | 0.634 |
| *Mussismilia harttii* | *Chilomycterus spinosus spinosus* | 2 | -0.29 | -3.76 | 2.8 | -14.51 | -49.45 | 11.66 | -6.97 | -58.82 | 49.14 | 0.544 |
| *Mussismilia harttii* | *Holocentrus adscensionis* | 2 | -0.13 | -3.11 | 2.84 | -22.02 | -59.23 | 1.52 | -23.82 | -67.57 | 24.2 | 0.328 |
| *Mussismilia harttii* | *Pseudupeneus maculatus* | 2 | -2.25 | -4.88 | 1.22 | -10.62 | -49.91 | -0.12 | -0.64 | -19.79 | 10.79 | 0.356 |
| *Mussismilia harttii* | *Serranus baldwini* | 2 | -1.45 | -4.13 | 1.89 | -16.72 | -60.52 | -0.57 | -0.72 | -19.32 | 15.21 | 0.368 |
| *Mussismilia harttii* | *Stephanolepis hispidus* | 2 | -3.04 | -5.11 | -1.64 | -3.53 | -10.15 | 0.01 | 0.04 | -0.81 | 0.97 | 0.41 |
| *Mussismilia harttii* | *Bodianus rufus* | 2 | -2.37 | -4.05 | -0.31 | 2 | -0.19 | 10.65 | 3.57 | -0.29 | 21.72 | 0.507 |
| *Mussismilia harttii* | *Diplodus argenteus* | 2 | 0.03 | -2.95 | 3.02 | -33.7 | -71.31 | -4.33 | 10.08 | -23.6 | 44.58 | 0.524 |
| *Mussismilia harttii* | *Halichoeres dimidiatus* | 2 | -1.35 | -4.86 | 2.3 | -6.41 | -40.33 | 20.6 | -21.68 | -62.54 | 0.49 | 0.194 |
| *Mussismilia harttii* | *Sparisoma tuiupiranga* | 2 | -0.29 | -3.36 | 2.77 | -6.78 | -46.44 | 31 | -29.54 | -70.71 | 8.61 | 0.192 |
| *Mussismilia harttii* | *Eucinostomus melanopterus* | 2 | -0.5 | -4.09 | 2.62 | 29.45 | 1.64 | 62.42 | 8.77 | 1.43 | 18.84 | 0.522 |
| *Mussismilia harttii* | *Sphoeroides greeleyi* | 2 | -0.15 | -3.41 | 2.65 | 7.08 | -41.55 | 48.04 | 26.14 | 0.1 | 63.73 | 0.689 |
| *Mussismilia harttii* | *Sphoeroides testudineus* | 2 | -0.21 | -3.43 | 2.92 | 4.32 | -46.74 | 46.6 | 24.9 | -7.56 | 63.99 | 0.674 |
| *Mussismilia harttii* | *Coryphopterus glaucofraenum* | 2 | -0.16 | -3.26 | 2.83 | 4.55 | -43.3 | 47.61 | 30.06 | 1.53 | 67.06 | 0.636 |
| *Mussismilia harttii* | *Synodus synodus* | 2 | -0.25 | -3.56 | 2.87 | -9.18 | -56.4 | 35.33 | 19.16 | -16.99 | 61.06 | 0.727 |
| *Mussismilia harttii* | *Pareques acuminatus* | 2 | -0.09 | -3.34 | 2.98 | -5.53 | -54.86 | 32.87 | 22.43 | -26.96 | 65.3 | 0.782 |
| *Mussismilia harttii* | *Sparisoma amplum* | 2 | -0.52 | -3.6 | 2.22 | 40.98 | 11.54 | 77.07 | 1.53 | -8.74 | 13.82 | 0.545 |
| *Mussismilia hispida* | *Acanthurus chirurgus* | 1 | 1.15 | 0.41 | 2.05 | 1.49 | 0.06 | 3.65 | -0.43 | -1.21 | 0.27 | 0 |
| *Mussismilia hispida* | *Chaetodon ocellatus* | 1 | -2.94 | -5.02 | -1.48 | -3.05 | -8.19 | 0.04 | -0.79 | -1.66 | 0 | 0.627 |
| *Mussismilia hispida* | *Haemulon plumierii* | 1 | -0.56 | -1.6 | 0.74 | -1.06 | -3.47 | 0.58 | -0.94 | -3.11 | 0.09 | 0.042 |
| *Mussismilia hispida* | *Halichoeres dimidiatus* | 1 | -0.96 | -2.12 | 0.59 | 1.43 | 0.19 | 3.5 | -1.28 | -4.14 | 0.08 | 0.102 |
| *Mussismilia hispida* | *Ocyurus chrysurus* | 1 | 0.01 | -1.54 | 2.86 | 2.54 | -1.45 | 11.01 | -0.18 | -0.93 | 0.55 | 0.002 |
| *Mussismilia hispida* | *Pomacanthus arcuatus* | 1 | -2.55 | -4.03 | -1.32 | -1.19 | -4.32 | 0.75 | 0.53 | -0.55 | 1.81 | 0.326 |
| *Mussismilia hispida* | *Pseudupeneus maculatus* | 1 | 2.32 | 0.8 | 4.97 | 2.69 | -0.03 | 8.25 | -0.94 | -2.87 | 0.22 | 0.013 |
| *Mussismilia hispida* | *Sparisoma axillare* | 1 | 3.09 | 1.34 | 5.82 | 5.13 | 0.73 | 12.36 | -0.87 | -1.9 | 0 | 0.002 |
| *Mussismilia hispida* | *Sparisoma frondosum* | 1 | 2.31 | 0.78 | 5.07 | 3.55 | -0.1 | 10.92 | -0.01 | -0.78 | 0.68 | 0.029 |
| *Mussismilia hispida* | *Thalassoma noronhanum* | 1 | -1.34 | -2.37 | -0.51 | -1.76 | -4.25 | -0.1 | -0.63 | -1.35 | 0.03 | 0 |
| *Mussismilia hispida* | *Abudefduf saxatilis* | 1 | 4.99 | 2.63 | 8.42 | 2.09 | -0.86 | 7.91 | -0.28 | -2.68 | 1.44 | 0 |
| *Mussismilia hispida* | *Acanthurus bahianus* | 1 | 2.93 | 1.19 | 5.65 | 5.31 | 0.78 | 12.59 | 0.14 | -0.55 | 0.8 | 0.013 |
| *Mussismilia hispida* | *Acanthurus coeruleus* | 1 | 0.35 | -0.49 | 1.26 | -1.65 | -3.53 | -0.37 | -1.3 | -2.62 | -0.33 | 0.058 |
| *Mussismilia hispida* | *Anisotremus virginicus* | 1 | 2.89 | 1.29 | 5.3 | 4 | 0.38 | 10.19 | -0.23 | -1.61 | 0.81 | 0.013 |
| *Mussismilia hispida* | *Bodianus rufus* | 1 | 0.65 | -0.13 | 1.65 | 0.15 | -1.06 | 2.3 | 0.53 | -0.22 | 1.37 | 0.008 |
| *Mussismilia hispida* | *Carangoides bartholomaei* | 1 | -1.75 | -3.99 | -0.13 | -6.59 | -15.01 | -1 | -0.41 | -1.45 | 0.63 | 0.02 |
| *Mussismilia hispida* | *Caranx ruber* | 1 | -1.78 | -5.14 | 2.08 | -14.69 | -57.24 | 0.77 | -5.79 | -28.78 | 1.06 | 0.379 |
| *Mussismilia hispida* | *Haemulon aurolineatum* | 1 | 1.92 | 0.58 | 3.88 | 3.91 | 0.47 | 9.39 | 0.13 | -0.51 | 0.81 | 0 |
| *Mussismilia hispida* | *Haemulon parra* | 1 | -0.42 | -1.82 | 1.79 | -0.79 | -4.29 | 4.61 | -0.73 | -1.91 | 0.19 | 0.539 |
| *Mussismilia hispida* | *Halichoeres bivittatus* | 1 | -0.36 | -3.45 | 2.51 | 5.18 | -29.24 | 36.53 | -36.89 | -75.33 | -6.13 | 0.256 |
| *Mussismilia hispida* | *Halichoeres brasiliensis* | 1 | 2.54 | 0.71 | 5.26 | 6.93 | 1.83 | 14.17 | 0.76 | 0.04 | 1.55 | 0.001 |
| *Mussismilia hispida* | *Halichoeres poeyi* | 1 | 1.48 | 0.57 | 2.72 | 1.63 | -0.04 | 4.52 | 0.35 | -0.37 | 1.12 | 0.015 |
| *Mussismilia hispida* | *Holacanthus ciliaris* | 1 | -0.29 | -1.6 | 1.32 | -2.73 | -7.36 | 0.03 | -2.59 | -6.56 | -0.17 | 0.074 |
| *Mussismilia hispida* | *Holocentrus adscensionis* | 1 | 1.56 | 0.71 | 2.59 | 1.4 | -0.08 | 3.68 | 0.23 | -0.5 | 0.98 | 0 |
| *Mussismilia hispida* | *Lutjanus jocu* | 1 | -0.42 | -1.38 | 0.7 | 1.04 | -0.58 | 3.76 | -0.72 | -1.7 | 0.06 | 0.061 |
| *Mussismilia hispida* | *Pomacanthus paru* | 1 | 0.83 | -0.14 | 2.23 | 2.64 | 0.35 | 6.43 | -0.06 | -0.72 | 0.59 | 0.004 |
| *Mussismilia hispida* | *Scarus trispinosus* | 1 | -0.98 | -2.14 | 0.55 | -0.54 | -3.38 | 3.3 | -1.5 | -5.26 | -0.09 | 0.005 |
| *Mussismilia hispida* | *Scarus zelindae* | 1 | 1.97 | 0.05 | 4.65 | 7.98 | 2.52 | 15.52 | -0.28 | -1.01 | 0.46 | 0 |
| *Mussismilia hispida* | *Sparisoma amplum* | 1 | -0.68 | -2.1 | 1.53 | -0.99 | -4.49 | 3.24 | 0.33 | -0.66 | 1.47 | 0.172 |
| *Mussismilia hispida* | *Sparisoma radians* | 1 | 2.2 | 0.07 | 5.15 | 4.98 | -0.85 | 11.79 | -2.56 | -6.38 | 0.41 | 0.734 |
| *Mussismilia hispida* | *Sphyraena barracuda* | 1 | -2.62 | -5.87 | 1.18 | 1.46 | -9.61 | 26.45 | -14.73 | -65.44 | -0.08 | 0.111 |
| *Mussismilia hispida* | *Stegastes variabilis* | 1 | -0.5 | -1.98 | 2.06 | -0.59 | -4.1 | 5.61 | 0.88 | -0.34 | 2.25 | 0.32 |
| *Mussismilia hispida* | *Chaetodon striatus* | 1 | 2.81 | 1.09 | 5.66 | 2.34 | -0.2 | 7.72 | 0.98 | -0.1 | 2.41 | 0 |
| *Mussismilia hispida* | *Lutjanus apodus* | 1 | -0.43 | -3.79 | 2.59 | 11.08 | -28.91 | 52.92 | -28.68 | -65.92 | -3.21 | 0.735 |
| *Mussismilia hispida* | *Stegastes fuscus* | 1 | 2.54 | 0.98 | 5.11 | 5.25 | 1.05 | 11.83 | 0.66 | -0.01 | 1.42 | 0.016 |
| *Mussismilia hispida* | *Acanthostracion polygonius* | 1 | 1 | -1.38 | 3.73 | 3.26 | -2.61 | 12.83 | -32.91 | -69.74 | -4.76 | 0.363 |
| *Mussismilia hispida* | *Amblycirrhitus pinos* | 1 | -1.07 | -4.71 | 2.36 | 0.61 | -38.63 | 32.85 | -23.28 | -67.12 | 10.27 | 0.437 |
| *Mussismilia hispida* | *Anisotremus surinamensis* | 1 | 1.24 | -1.13 | 4.6 | -0.89 | -8.47 | 6.62 | 2.3 | -0.28 | 6.64 | 0.34 |
| *Mussismilia hispida* | *Aulostomus maculatus* | 1 | -0.25 | -3.32 | 2.64 | -3.05 | -46.96 | 33.29 | -28.23 | -66.57 | 17.89 | 0.738 |
| *Mussismilia hispida* | *Cantherhines pullus* | 1 | -1.17 | -1.95 | -0.45 | 0.92 | 0.03 | 2.23 | -0.15 | -0.87 | 0.6 | 0.092 |
| *Mussismilia hispida* | *Canthidermis maculata* | 1 | -1.14 | -2.71 | 1.48 | 2.38 | -1.62 | 11.72 | -0.1 | -1.11 | 1.05 | 0.183 |
| *Mussismilia hispida* | *Cephalopholis fulva* | 1 | -1.54 | -2.51 | -0.71 | -1.2 | -3.47 | 0.21 | -0.24 | -0.96 | 0.47 | 0.206 |
| *Mussismilia hispida* | *Chromis multilineata* | 1 | 0.78 | -0.3 | 2.28 | 2.45 | 0.4 | 6.04 | 0.93 | -0.01 | 2.17 | 0.001 |
| *Mussismilia hispida* | *Clepticus brasiliensis* | 1 | 1.84 | -0.96 | 4.94 | 8.26 | -0.04 | 17.58 | -6.84 | -17.06 | 0.37 | 0.389 |
| *Mussismilia hispida* | *Epinephelus adscensionis* | 1 | -0.85 | -2.34 | 1.78 | -1.36 | -14.28 | 4.53 | 1.77 | -0.7 | 13.54 | 0.632 |
| *Mussismilia hispida* | *Haemulon squamipinna* | 1 | 0.85 | -2.61 | 4.55 | 12.7 | -2.01 | 35.65 | -15.5 | -40.48 | 0.15 | 0.191 |
| *Mussismilia hispida* | *Halichoeres penrosei* | 1 | -0.53 | -3.38 | 2.27 | -13.19 | -33.85 | -1.2 | -36.31 | -75.4 | -9.25 | 0.036 |
| *Mussismilia hispida* | *Microspathodon chrysurus* | 1 | -1.29 | -2.51 | 0.16 | -0.14 | -3.03 | 4.1 | 0.08 | -0.8 | 1.08 | 0.61 |
| *Mussismilia hispida* | *Ophioblennius macclurei* | 1 | -0.14 | -3.15 | 2.89 | -2.46 | -44.31 | 35.3 | -30.99 | -70.5 | 10.31 | 0.792 |
| *Mussismilia hispida* | *Scomberomorus regalis* | 1 | -0.14 | -3.11 | 2.69 | 0.11 | -42.45 | 34.67 | -31.6 | -69.93 | 2.04 | 0.802 |
| *Mussismilia hispida* | *Serranus baldwini* | 1 | -0.9 | -1.79 | 0.03 | 2.21 | 0.5 | 4.51 | 0.2 | -0.65 | 1.11 | 0.014 |
| *Mussismilia hispida* | *Halichoeres radiatus* | 1 | -1.77 | -3.49 | -0.6 | -3.53 | -8.16 | -0.61 | -0.27 | -0.97 | 0.37 | 0.001 |
| *Mussismilia hispida* | *Holacanthus tricolor* | 1 | -1 | -3.9 | 2.25 | -13.49 | -41.6 | -0.49 | -18.54 | -63.21 | 1.06 | 0.237 |
| *Mussismilia hispida* | *Lutjanus analis* | 1 | -0.34 | -3.5 | 2.64 | 8.51 | -34.58 | 49.21 | -27.36 | -64.19 | -1.05 | 0.743 |
| *Mussismilia hispida* | *Mycteroperca bonaci* | 1 | 1.12 | -1.44 | 4.28 | -8.45 | -48.99 | 11.75 | -5.4 | -21.58 | 0.99 | 0.404 |
| *Mussismilia hispida* | *Pseudocaranx dentex* | 1 | 0.56 | -1.42 | 3.23 | 4.35 | 0.07 | 11.67 | 5.27 | 0.7 | 12.11 | 0.607 |
| *Mussismilia hispida* | *Bothus ocellatus* | 1 | -0.66 | -4.55 | 2.57 | 10.15 | -44.83 | 56.05 | 20.67 | -0.17 | 59.84 | 0.598 |
| *Mussismilia hispida* | *Coryphopterus glaucofraenum* | 1 | -0.5 | -2.1 | 1.99 | 4.65 | 0.39 | 13.37 | 1.05 | -0.95 | 2.5 | 0.588 |
| *Mussismilia hispida* | *Malacanthus plumieri* | 1 | -1.83 | -2.76 | -1.01 | 0.19 | -0.71 | 1 | 0.42 | -0.44 | 1.4 | 0.696 |
| *Mussismilia hispida* | *Stegastes rocasensis* | 1 | -2.75 | -4.98 | -1.32 | -3.85 | -9.81 | -0.33 | -0.17 | -0.89 | 0.56 | 0.437 |
| *Mussismilia hispida* | *Apogon americanus* | 1 | -0.12 | -3.54 | 2.88 | -20.25 | -57.06 | 4.56 | 2.72 | -50.33 | 57.7 | 0.666 |
| *Mussismilia hispida* | *Aluterus scriptus* | 1 | -0.81 | -3.67 | 2.53 | -24.33 | -72.8 | -0.16 | 1.24 | -9.79 | 12.99 | 0.227 |
| *Mussismilia hispida* | *Melichthys niger* | 1 | -3.64 | -5.84 | -2.03 | -2.81 | -8.18 | 0.23 | -0.29 | -1.28 | 0.75 | 0.42 |
| *Mussismilia hispida* | *Rypticus saponaceus* | 1 | -0.03 | -2.9 | 2.9 | 9.22 | -26.16 | 44.08 | 33.7 | 6.98 | 68.26 | 0.82 |
| *Mussismilia hispida* | *Mulloidichthys martinicus* | 1 | -0.91 | -3.01 | 2.28 | 1.29 | -3.62 | 9.72 | 0.24 | -1.35 | 1.91 | 0.449 |
| *Mussismilia hispida* | *Caranx latus* | 1 | -0.41 | -2.87 | 2.03 | 30.7 | 5.16 | 63.97 | 18.68 | 4.05 | 42.16 | 0.547 |
| *Mussismilia hispida* | *Diodon hystrix* | 1 | 0.44 | -2.26 | 3.33 | -0.08 | -10.48 | 10.75 | -32.55 | -76.01 | 1.74 | 0.401 |
| *Mussismilia hispida* | *Cryptotomus roseus* | 1 | -0.1 | -1.3 | 2.89 | 2.55 | 0.06 | 10.95 | 0.4 | -0.4 | 1.29 | 0 |
| *Mussismilia hispida* | *Sparisoma tuiupiranga* | 1 | 0.33 | -1.18 | 3.08 | 5.38 | 0.94 | 13.43 | -0.09 | -0.89 | 0.76 | 0.045 |
| *Mussismilia hispida* | *Odontoscion dentex* | 1 | -2.95 | -6.11 | 0.51 | -2.5 | -13.42 | 19.98 | 8.19 | 0.56 | 42.35 | 0.484 |
| *Mussismilia hispida* | *Aluterus monoceros* | 1 | -0.26 | -3.48 | 2.81 | 7.08 | -48.14 | 55.5 | 22.87 | -0.24 | 62.71 | 0.683 |
| *Mussismilia hispida* | *Haemulon steindachneri* | 1 | -0.9 | -4.25 | 2.17 | 10.65 | -9.65 | 39.13 | 34.43 | 9.57 | 69.17 | 0.503 |
| *Mussismilia hispida* | *Lactophrys trigonus* | 1 | -0.28 | -3.65 | 2.74 | 5.99 | -44.41 | 52.77 | 21.36 | -0.69 | 56.64 | 0.663 |
| *Mussismilia hispida* | *Mycteroperca acutirostris* | 1 | -0.33 | -2.15 | 1.81 | 4.65 | 0.78 | 12.59 | 10.36 | 2.01 | 34.19 | 0.208 |
| *Mussismilia hispida* | *Synodus intermedius* | 1 | 0.06 | -2.81 | 3.02 | -18.9 | -60.05 | 12.56 | 26.62 | -4.17 | 67.15 | 0.748 |
| *Mussismilia hispida* | *Balistes vetula* | 1 | -0.23 | -3.53 | 3.37 | 24.44 | 1.03 | 59.77 | 8.81 | -1.05 | 35.66 | 0.219 |
| *Mussismilia hispida* | *Canthigaster figueiredoi* | 1 | 1.12 | -0.62 | 3.93 | 3.28 | 0.56 | 8.84 | 1.88 | 0.13 | 5.14 | 0.002 |
| *Mussismilia hispida* | *Malacoctenus delalandii* | 1 | 0.11 | -3.1 | 3.1 | 11.62 | -43.91 | 60.59 | 19.57 | -2.41 | 56.81 | 0.593 |
| *Mussismilia hispida* | *Anisotremus moricandi* | 1 | -1.68 | -5.61 | 2.34 | -11.74 | -46.66 | 2.2 | -5.81 | -53.01 | 35.96 | 0.368 |
| *Mussismilia hispida* | *Chilomycterus spinosus spinosus* | 1 | -0.62 | -2.52 | 2.91 | 3.19 | 0.29 | 11.2 | -0.42 | -4.24 | 1.58 | 0.402 |
| *Mussismilia hispida* | *Diplodus argenteus* | 1 | -0.32 | -1.14 | 0.47 | 1.8 | 0.47 | 3.71 | 1.81 | 0.74 | 3.04 | 0.281 |
| *Mussismilia hispida* | *Sphoeroides spengleri* | 1 | 0.75 | -0.72 | 3.11 | 1.49 | 0 | 4.69 | 2.77 | 0.56 | 6.36 | 0.154 |
| *Mussismilia hispida* | *Dactylopterus volitans* | 1 | -0.38 | -4.03 | 2.9 | -17.17 | -55.2 | 8.54 | 7.31 | -48.24 | 61.05 | 0.544 |
| *Mussismilia hispida* | *Kyphosus sectatrix* | 1 | 0.36 | -2.58 | 3.43 | 7.31 | 0.47 | 19.27 | 27.64 | -7.07 | 77.71 | 0.599 |
| *Mussismilia hispida* | *Aulostomus strigosus* | 1 | -0.16 | -3.52 | 3.04 | -18.44 | -54.68 | 2.95 | -4.77 | -56.46 | 50.61 | 0.621 |
| *Mussismilia hispida* | *Caranx crysos* | 1 | -0.06 | -2.8 | 2.76 | -20.87 | -53.73 | 0.07 | -14.39 | -63.58 | 46.49 | 0.539 |
| *Mussismilia hispida* | *Fistularia tabacaria* | 1 | -0.25 | -3.28 | 2.71 | 18.88 | 1.47 | 46.67 | 28.09 | 0.37 | 64.21 | 0.557 |
| *Mussismilia hispida* | *Chaetodon sedentarius* | 1 | -1.83 | -2.81 | -0.92 | 1.36 | 0.26 | 3.21 | 0.14 | -0.8 | 1.22 | 0.506 |
| *Mussismilia hispida* | *Priacanthus arenatus* | 1 | -2.75 | -6.07 | 1.55 | -6.82 | -32.51 | 1.38 | 4.37 | -21.07 | 44.71 | 0.34 |
| *Mussismilia hispida* | *Bodianus pulchellus* | 1 | -1.35 | -2.3 | -0.41 | 2.26 | 0.71 | 4.55 | 0.47 | -0.46 | 1.53 | 0.09 |
| *Mussismilia hispida* | *Elacatinus figaro* | 1 | -0.08 | -3.51 | 3.03 | -16.79 | -50.44 | 5.84 | -9.84 | -63.37 | 47.26 | 0.528 |
| *Mussismilia hispida* | *Sphoeroides greeleyi* | 1 | -0.01 | -3.35 | 3.14 | -16.86 | -52.05 | 5.98 | -13.53 | -67.66 | 44.11 | 0.492 |
| *Mussismilia hispida* | *Stephanolepis hispidus* | 1 | -0.94 | -1.83 | -0.04 | 2.6 | 1 | 4.9 | 0.53 | -0.34 | 1.56 | 0.012 |
| *Mussismilia hispida* | *Epinephelus morio* | 1 | -0.41 | -3.34 | 2.58 | 14.95 | 1.49 | 38.08 | 26.1 | 1.3 | 64.14 | 0.247 |
| *Mussismilia hispida* | *Heteropriacanthus cruentatus* | 1 | -1.32 | -5.82 | 2.24 | 27.89 | 1.24 | 69.88 | -2.17 | -39.67 | 19.02 | 0.372 |
| *Mussismilia hispida* | *Labrisomus nuchipinnis* | 1 | -0.32 | -3.43 | 2.53 | 20.87 | 1.61 | 49.89 | 26.15 | 0.59 | 60.04 | 0.423 |
| *Mussismilia hispida* | *Myrichthys ocellatus* | 1 | -1.29 | -5.6 | 2.18 | 28.64 | 1.21 | 70.94 | -2.21 | -39.18 | 21.5 | 0.366 |
| *Mussismilia hispida* | *Acanthostracion quadricornis* | 1 | -0.49 | -3.73 | 2.5 | 34.71 | 3.65 | 72.33 | -1.42 | -39.01 | 21.62 | 0.336 |
| *Mussismilia hispida* | *Epinephelus marginatus* | 1 | -0.63 | -3.63 | 2.3 | 18.71 | 1.87 | 43.48 | 35.58 | 7.78 | 70.51 | 0.024 |
| *Mussismilia hispida* | *Mugil curema* | 1 | -0.46 | -4.05 | 2.56 | 11.14 | -30.55 | 53.25 | -26.35 | -65 | -0.14 | 0.365 |
| *Mussismilia hispida* | *Orthopristis ruber* | 1 | 0.11 | -2.61 | 3.09 | -4.64 | -33.21 | 17.28 | 35.96 | 5.51 | 73.57 | 0.152 |
| *Mussismilia hispida* | *Kyphosus vaigiensis* | 1 | -2.46 | -6.86 | 2.18 | 3.52 | -28.98 | 46.01 | 12.17 | -0.27 | 48.13 | 0.155 |
| *Mussismilia hispida* | *Mycteroperca interstitialis* | 1 | -1 | -5.03 | 2.49 | 7.46 | -36.51 | 46.4 | 20.74 | -0.43 | 55.14 | 0.521 |
| *Mussismilia hispida* | *Eucinostomus melanopterus* | 1 | -0.56 | -4.33 | 2.66 | 16.92 | -36.78 | 66.25 | 19.81 | 1.05 | 53.15 | 0.545 |
| *Mussismilia hispida* | *Lutjanus cyanopterus* | 1 | -1.23 | -5.8 | 2.31 | 11.12 | -39.45 | 60.52 | 17.98 | 0.37 | 50.06 | 0.445 |
| *Mussismilia hispida* | *Calamus penna* | 1 | -0.62 | -3.99 | 2.4 | 35.53 | 3.77 | 75.38 | 1.51 | -27.09 | 24.58 | 0.54 |
| *Mussismilia hispida* | *Seriola rivoliana* | 1 | -0.58 | -4.11 | 2.36 | 34.31 | 3.39 | 71.85 | 0.88 | -33.13 | 28.91 | 0.544 |
| *Mussismilia hispida* | *Parablennius pilicornis* | 1 | -0.13 | -3.45 | 2.89 | -12.53 | -63.95 | 35.97 | 17.84 | -25.37 | 61.67 | 0.745 |
| *Mussismilia hispida* | *Pareques acuminatus* | 1 | -0.25 | -3.73 | 2.76 | -7.55 | -57.83 | 37.71 | 19.99 | -22.31 | 62.24 | 0.735 |
| *Mussismilia hispida* | *Rhomboplites aurorubens* | 1 | -4.17 | -6.86 | -2.18 | -2.55 | -8.51 | 0.65 | 1.1 | -0.62 | 3.46 | 0.425 |
| *Mussismilia hispida* | *Sphoeroides testudineus* | 1 | -0.08 | -3.31 | 3.03 | -7.1 | -53.41 | 30.93 | 27.02 | -16.64 | 66.73 | 0.816 |
| *Mussismilia hispida* | *Myripristis jacobus* | 1 | -0.31 | -3.36 | 2.64 | 37.28 | 6.05 | 75.85 | 4.4 | -17.15 | 32.71 | 0.65 |
| *Mussismilia hispida* | *Acanthurus bahianus* | 2 | -0.83 | -2.05 | 0.82 | -0.91 | -3.6 | 2.82 | 1.15 | 0.01 | 2.88 | 0.008 |
| *Mussismilia hispida* | *Acanthurus chirurgus* | 2 | -3.09 | -5.5 | -1.49 | -4.36 | -10.79 | -0.39 | -1.13 | -1.97 | -0.36 | 0.596 |
| *Mussismilia hispida* | *Sparisoma frondosum* | 2 | -0.8 | -2.8 | 1.89 | -14.26 | -60.74 | -0.3 | -3.15 | -16.01 | 1.38 | 0.417 |
| *Mussismilia hispida* | *Sparisoma axillare* | 2 | -0.5 | -3.55 | 3.43 | -12.51 | -54.26 | 2.86 | -5.48 | -19.41 | 0.54 | 0.423 |
| *Mussismilia hispida* | *Sparisoma radians* | 2 | -0.04 | -1.61 | 2.33 | 2.86 | -1.1 | 9.63 | -1.09 | -3 | 0.06 | 0.315 |
| *Mussismilia hispida* | *Stegastes variabilis* | 2 | 1.48 | -0.98 | 4.23 | -20.3 | -57.49 | 3.29 | -8.64 | -23.23 | 0.48 | 0.496 |
| *Mussismilia hispida* | *Abudefduf saxatilis* | 2 | 2.66 | 1.05 | 4.99 | 3.65 | -0.1 | 9.98 | 1.03 | 0.17 | 2 | 0.027 |
| *Mussismilia hispida* | *Acanthurus coeruleus* | 2 | -1.32 | -2.45 | 0.07 | -0.28 | -2.45 | 3.23 | -0.97 | -2.22 | -0.04 | 0.1 |
| *Mussismilia hispida* | *Chromis multilineata* | 2 | 0.94 | -0.89 | 3.76 | 1.71 | -2.47 | 8.19 | 0.26 | -1.44 | 1.87 | 0.035 |
| *Mussismilia hispida* | *Halichoeres brasiliensis* | 2 | -1.91 | -4.54 | 1.71 | -11.14 | -53.83 | 2.05 | 4.06 | -2.94 | 27.33 | 0.394 |
| *Mussismilia hispida* | *Halichoeres poeyi* | 2 | 0.43 | -0.53 | 1.74 | 3.56 | 1.02 | 7.23 | 0.37 | -0.36 | 1.15 | 0.006 |
| *Mussismilia hispida* | *Holacanthus tricolor* | 2 | -0.27 | -3.37 | 2.68 | -4.26 | -45.19 | 32.42 | -28.76 | -68.54 | 11.71 | 0.773 |
| *Mussismilia hispida* | *Microspathodon chrysurus* | 2 | -0.76 | -4.46 | 2.38 | 7.51 | -33.03 | 47.36 | -26.37 | -64.15 | -0.23 | 0.637 |
| *Mussismilia hispida* | *Anisotremus virginicus* | 2 | 0.2 | -2.54 | 3.8 | 3.1 | -1.11 | 10.63 | 0.94 | -5.2 | 7.7 | 0.392 |
| *Mussismilia hispida* | *Haemulon aurolineatum* | 2 | -1.18 | -3.45 | 1.32 | -20.33 | -53.3 | -1.18 | 16.67 | 1.14 | 46.14 | 0.183 |
| *Mussismilia hispida* | *Stegastes fuscus* | 2 | -1.1 | -2.05 | -0.25 | -1.6 | -3.84 | -0.01 | 1.03 | 0.16 | 2.11 | 0.185 |
| *Mussismilia hispida* | *Stegastes rocasensis* | 2 | -2.53 | -4.74 | -1.03 | -3.86 | -9.88 | -0.29 | -0.17 | -0.93 | 0.59 | 0.126 |
| *Mussismilia hispida* | *Thalassoma noronhanum* | 2 | -2.75 | -4.86 | -1.3 | -3.9 | -9.51 | -0.34 | -0.16 | -0.89 | 0.57 | 0.204 |
| *Mussismilia hispida* | *Halichoeres radiatus* | 2 | 0.24 | -2.29 | 2.97 | -36.78 | -77.59 | -2.35 | -1.69 | -13.76 | 7.61 | 0.152 |
| *Mussismilia hispida* | *Cantherhines pullus* | 2 | -0.25 | -3.17 | 2.66 | 20.39 | 0.5 | 49.31 | 25.56 | -0.16 | 62.69 | 0.177 |
| *Mussismilia hispida* | *Cryptotomus roseus* | 2 | -1 | -2.73 | 1.78 | 10.79 | -0.74 | 42.25 | 11.76 | -0.16 | 47.22 | 0.019 |
| *Mussismilia hispida* | *Haemulon plumierii* | 2 | -3.29 | -5.97 | 0.51 | 0.23 | -7.93 | 22.96 | 2.71 | -0.76 | 16.02 | 0.334 |
| *Mussismilia hispida* | *Canthigaster figueiredoi* | 2 | 0.01 | -2.72 | 2.75 | 23.81 | 5.2 | 51.97 | 28.28 | 3.98 | 58.79 | 0.396 |
| *Mussismilia hispida* | *Chaetodon striatus* | 2 | -0.43 | -3.65 | 2.66 | 7.1 | -38.5 | 48.22 | 23.97 | 0.35 | 59.2 | 0.36 |
| *Mussismilia hispida* | *Mycteroperca bonaci* | 2 | -0.28 | -3.63 | 2.67 | 7.6 | -43.52 | 54.02 | 23.26 | -0.31 | 62.02 | 0.673 |
| *Mussismilia hispida* | *Pomacanthus paru* | 2 | -0.19 | -3.17 | 2.79 | -23.26 | -58.47 | -0.49 | 26.86 | 1.32 | 64.85 | 0.294 |
| *Mussismilia hispida* | *Scarus zelindae* | 2 | 1.33 | -1.59 | 4.98 | 12.75 | 0.87 | 25.83 | 5.91 | -0.44 | 32.98 | 0.038 |
| *Mussismilia hispida* | *Sphoeroides spengleri* | 2 | -0.39 | -1.32 | 0.74 | 0.86 | -0.28 | 2.98 | 1.55 | 0.29 | 3.84 | 0.102 |
| *Mussismilia hispida* | *Ocyurus chrysurus* | 2 | 0.12 | -2.98 | 3.11 | 11.68 | -44.5 | 60.49 | 22.44 | -1.19 | 63.6 | 0.578 |
| *Mussismilia hispida* | *Labrisomus nuchipinnis* | 2 | -0.14 | -3.33 | 2.78 | 4.47 | -46.34 | 49.41 | 27.2 | -2.36 | 64.01 | 0.717 |
| *Mussismilia hispida* | *Bodianus pulchellus* | 2 | -0.03 | -2.95 | 2.9 | -21.95 | -57.99 | 1.59 | 24.38 | -8.19 | 64.28 | 0.639 |
| *Mussismilia hispida* | *Chilomycterus spinosus spinosus* | 2 | -0.2 | -3.8 | 2.94 | -16.38 | -49.89 | 6.34 | -6.97 | -62.71 | 48.28 | 0.524 |
| *Mussismilia hispida* | *Holocentrus adscensionis* | 2 | -0.14 | -3.57 | 3.44 | 3.97 | -1.81 | 14.04 | -15.62 | -64.19 | 6.21 | 0.329 |
| *Mussismilia hispida* | *Pseudupeneus maculatus* | 2 | -1.98 | -3.19 | -0.81 | 1.03 | -0.07 | 2.86 | 0.89 | -0.56 | 2.02 | 0.427 |
| *Mussismilia hispida* | *Serranus baldwini* | 2 | -1.11 | -2.05 | -0.17 | 1.57 | 0.19 | 3.81 | 0.31 | -0.6 | 1.35 | 0.515 |
| *Mussismilia hispida* | *Stephanolepis hispidus* | 2 | -2.36 | -3.48 | -1.37 | 1.11 | 0.3 | 2.06 | 0.13 | -0.86 | 1.31 | 0.42 |
| *Mussismilia hispida* | *Bodianus rufus* | 2 | -1.62 | -3.54 | 1.06 | 4.38 | -2.97 | 26.46 | 7.3 | -0.22 | 42.32 | 0.43 |
| *Mussismilia hispida* | *Diplodus argenteus* | 2 | 0 | -2.86 | 2.86 | 18.67 | 2.91 | 42 | 35.97 | 9.35 | 69.43 | 0.418 |
| *Mussismilia hispida* | *Halichoeres dimidiatus* | 2 | -2.97 | -6.78 | 1.22 | 16.76 | 1.4 | 65.06 | 0.87 | -5.39 | 10.17 | 0.187 |
| *Mussismilia hispida* | *Sparisoma tuiupiranga* | 2 | -1.16 | -5.1 | 2.16 | 32.34 | 2.42 | 74.89 | 1.13 | -17.58 | 18.02 | 0.164 |
| *Mussismilia hispida* | *Eucinostomus melanopterus* | 2 | -4.36 | -7.28 | -2.13 | -1.61 | -7.11 | 1.02 | 2.36 | -0.15 | 5.87 | 0.26 |
| *Mussismilia hispida* | *Sphoeroides greeleyi* | 2 | -0.22 | -3.53 | 2.75 | -0.99 | -47.7 | 43.02 | 26.44 | -6.38 | 65.71 | 0.721 |
| *Mussismilia hispida* | *Sphoeroides testudineus* | 2 | -0.27 | -3.67 | 2.78 | 2.02 | -45.42 | 45.22 | 27.1 | -1.14 | 63.23 | 0.725 |
| *Mussismilia hispida* | *Coryphopterus glaucofraenum* | 2 | -0.29 | -3.36 | 2.7 | -0.3 | -49.75 | 43.7 | 27.12 | -3.84 | 63 | 0.628 |
| *Mussismilia hispida* | *Synodus synodus* | 2 | -0.27 | -3.8 | 2.86 | -5.78 | -53.61 | 36.89 | 21.9 | -15.08 | 60.4 | 0.731 |
| *Mussismilia hispida* | *Pareques acuminatus* | 2 | -0.1 | -3.32 | 2.9 | -12.84 | -54.65 | 26.89 | 17.49 | -28.26 | 59 | 0.791 |
| *Mussismilia hispida* | *Sparisoma amplum* | 2 | -0.34 | -3.5 | 2.52 | 38.33 | 8.53 | 75.9 | 2.31 | -22.05 | 26.47 | 0.648 |
| *Porites astreoides* | *Acanthurus chirurgus* | 1 | 0.91 | 0.22 | 1.69 | -0.62 | -1.69 | 0.16 | -0.63 | -1.59 | 0.14 | 0 |
| *Porites astreoides* | *Chaetodon ocellatus* | 1 | -2.13 | -3.26 | -1.15 | -0.57 | -2.22 | 0.47 | -0.83 | -1.7 | -0.02 | 0.625 |
| *Porites astreoides* | *Haemulon plumierii* | 1 | 0.14 | -1.22 | 2.5 | -1.6 | -4.7 | 0.1 | -2.44 | -8.18 | -0.04 | 0.033 |
| *Porites astreoides* | *Halichoeres dimidiatus* | 1 | -0.84 | -2.99 | 1.92 | -4.27 | -21.52 | 8.18 | -3.73 | -14.8 | -0.01 | 0.087 |
| *Porites astreoides* | *Ocyurus chrysurus* | 1 | 0.44 | -1.21 | 2.62 | 7.31 | 1.35 | 15.92 | -0.21 | -1.02 | 0.66 | 0.002 |
| *Porites astreoides* | *Pomacanthus arcuatus* | 1 | -3.14 | -5.32 | -1.6 | -4.3 | -11.61 | -0.03 | 0.53 | -0.53 | 1.83 | 0.335 |
| *Porites astreoides* | *Pseudupeneus maculatus* | 1 | 1.16 | -0.91 | 3.57 | -17.56 | -52.78 | -3.56 | -2.42 | -7.23 | 1.96 | 0.008 |
| *Porites astreoides* | *Sparisoma axillare* | 1 | 2.4 | 1.03 | 4.62 | 3.12 | -0.35 | 11.11 | -1.11 | -2.35 | -0.11 | 0.001 |
| *Porites astreoides* | *Sparisoma frondosum* | 1 | 2.43 | 0.96 | 4.82 | 5.8 | 0.7 | 14.6 | 0.03 | -0.68 | 0.72 | 0.039 |
| *Porites astreoides* | *Thalassoma noronhanum* | 1 | -0.8 | -1.64 | 0.58 | 0.69 | -1.23 | 8.13 | -0.71 | -1.55 | 0.02 | 0.001 |
| *Porites astreoides* | *Abudefduf saxatilis* | 1 | 4.99 | 2.62 | 8.31 | 2.86 | -0.72 | 9.51 | -0.32 | -2.82 | 1.44 | 0 |
| *Porites astreoides* | *Acanthurus bahianus* | 1 | 1.92 | 0.82 | 4.03 | 2.62 | -0.24 | 10.16 | 0.13 | -0.6 | 0.85 | 0.008 |
| *Porites astreoides* | *Acanthurus coeruleus* | 1 | 1.37 | 0.04 | 3.64 | 3.9 | 0.13 | 11.92 | -1.3 | -2.84 | -0.29 | 0.033 |
| *Porites astreoides* | *Anisotremus virginicus* | 1 | 2.65 | 1.07 | 5.33 | 1.74 | -0.99 | 8.43 | -0.28 | -2.91 | 1.32 | 0.005 |
| *Porites astreoides* | *Bodianus rufus* | 1 | -0.18 | -1.39 | 0.85 | -4.56 | -9.22 | -1.21 | 0.64 | -0.19 | 1.54 | 0.012 |
| *Porites astreoides* | *Carangoides bartholomaei* | 1 | -0.16 | -1.17 | 1.42 | 0.95 | -1.47 | 7.91 | -0.38 | -1.38 | 0.4 | 0.017 |
| *Porites astreoides* | *Caranx ruber* | 1 | -1.72 | -4.94 | 1.83 | -6.79 | -46.36 | 13.48 | -3.5 | -18.91 | 0.49 | 0.398 |
| *Porites astreoides* | *Haemulon aurolineatum* | 1 | 0.87 | 0.18 | 1.67 | 0.52 | -0.46 | 1.89 | 0.2 | -0.45 | 0.9 | 0 |
| *Porites astreoides* | *Haemulon parra* | 1 | 0 | -1.98 | 2.85 | -13.13 | -55.78 | -0.35 | -0.9 | -4.79 | 2.97 | 0.467 |
| *Porites astreoides* | *Halichoeres bivittatus* | 1 | -0.29 | -3.35 | 2.6 | 13.69 | -20.8 | 47.15 | -33.45 | -70.4 | -6.39 | 0.273 |
| *Porites astreoides* | *Halichoeres brasiliensis* | 1 | 0.6 | -0.05 | 1.3 | 0.28 | -0.52 | 1.22 | 0.68 | -0.02 | 1.4 | 0.001 |
| *Porites astreoides* | *Halichoeres poeyi* | 1 | 0.53 | -1.27 | 2.62 | -14.71 | -47.7 | -3.34 | 0.76 | -2.18 | 3.63 | 0.007 |
| *Porites astreoides* | *Holacanthus ciliaris* | 1 | -0.1 | -1.27 | 1.77 | 1.87 | -0.34 | 7.89 | -1.02 | -3.13 | 0.15 | 0.129 |
| *Porites astreoides* | *Holocentrus adscensionis* | 1 | 1.4 | 0.58 | 2.41 | -0.62 | -2.07 | 0.31 | 0.25 | -0.58 | 1.08 | 0 |
| *Porites astreoides* | *Lutjanus jocu* | 1 | -0.3 | -1.35 | 1.28 | 2.41 | 0.08 | 7.52 | -0.76 | -1.84 | 0.07 | 0.062 |
| *Porites astreoides* | *Pomacanthus paru* | 1 | 0.36 | -0.35 | 1.33 | 1.01 | -0.25 | 3.43 | 0 | -0.69 | 0.67 | 0.005 |
| *Porites astreoides* | *Scarus trispinosus* | 1 | -1.1 | -2.06 | -0.05 | 1.82 | 0.16 | 4.3 | -0.91 | -1.96 | -0.02 | 0.007 |
| *Porites astreoides* | *Scarus zelindae* | 1 | -0.15 | -0.87 | 0.66 | 0.81 | -0.31 | 2.66 | -0.18 | -0.87 | 0.5 | 0.003 |
| *Porites astreoides* | *Sparisoma amplum* | 1 | 1.5 | -0.57 | 4.18 | 11.5 | 2.98 | 22.3 | -0.58 | -2.08 | 0.62 | 0.114 |
| *Porites astreoides* | *Sparisoma radians* | 1 | 1.12 | -1.41 | 4.21 | -31.8 | -74.43 | 1.54 | 0.61 | -5.68 | 7.07 | 0.696 |
| *Porites astreoides* | *Sphyraena barracuda* | 1 | -1.39 | -4.85 | 2.14 | 10.49 | -7.53 | 39.64 | -12.29 | -55.31 | -0.24 | 0.193 |
| *Porites astreoides* | *Stegastes variabilis* | 1 | -0.3 | -2.46 | 2.57 | -4.33 | -40.29 | 11.48 | 1.03 | -0.63 | 3.58 | 0.323 |
| *Porites astreoides* | *Chaetodon striatus* | 1 | 2.64 | -0.33 | 5.91 | -0.83 | -20.96 | 7.82 | 1.18 | -0.4 | 3.05 | 0 |
| *Porites astreoides* | *Lutjanus apodus* | 1 | -0.52 | -3.72 | 2.33 | 14.04 | -33.14 | 62.01 | -25.22 | -63.95 | -2.34 | 0.721 |
| *Porites astreoides* | *Stegastes fuscus* | 1 | 1.05 | 0.4 | 1.71 | 0.36 | -0.47 | 1.46 | 0.67 | 0.02 | 1.39 | 0.019 |
| *Porites astreoides* | *Acanthostracion polygonius* | 1 | -0.15 | -3.06 | 2.81 | -17.69 | -50.66 | 5.95 | -31.34 | -71.22 | 3.92 | 0.392 |
| *Porites astreoides* | *Amblycirrhitus pinos* | 1 | -0.9 | -4.64 | 2.31 | 11.53 | -17.61 | 46.8 | -25.26 | -67.32 | 0.89 | 0.442 |
| *Porites astreoides* | *Anisotremus surinamensis* | 1 | 0.13 | -2.76 | 3.16 | -34.08 | -75.48 | -1.77 | 4 | -6.47 | 14.21 | 0.338 |
| *Porites astreoides* | *Aulostomus maculatus* | 1 | -0.28 | -3.48 | 2.71 | 4.47 | -43.26 | 44.05 | -30.95 | -67.53 | 0.48 | 0.713 |
| *Porites astreoides* | *Cantherhines pullus* | 1 | -1.81 | -3.27 | -0.75 | -3.49 | -8.96 | -0.25 | -0.44 | -1 | 0.53 | 0.109 |
| *Porites astreoides* | *Canthidermis maculata* | 1 | -1.91 | -3.02 | -0.92 | 1.2 | 0.12 | 2.75 | 0.04 | -0.92 | 1.17 | 0.204 |
| *Porites astreoides* | *Cephalopholis fulva* | 1 | -1.34 | -2.15 | -0.6 | -0.53 | -1.97 | 0.47 | -0.25 | -0.99 | 0.5 | 0.202 |
| *Porites astreoides* | *Chromis multilineata* | 1 | 0.49 | -0.59 | 2.15 | -0.96 | -4.27 | 4.2 | 1.12 | 0.04 | 2.56 | 0.004 |
| *Porites astreoides* | *Clepticus brasiliensis* | 1 | -0.85 | -3.8 | 2.22 | -5.97 | -40.15 | 9.43 | 0.15 | -3.6 | 5.37 | 0.411 |
| *Porites astreoides* | *Epinephelus adscensionis* | 1 | -0.22 | -2.08 | 2.9 | 4.6 | -1.82 | 19.06 | 0.56 | -0.69 | 2.14 | 0.469 |
| *Porites astreoides* | *Haemulon squamipinna* | 1 | 0.03 | -2.43 | 3.21 | 8.37 | -1.19 | 23.76 | -2.45 | -10.6 | 0.5 | 0.18 |
| *Porites astreoides* | *Halichoeres penrosei* | 1 | -0.5 | -3.26 | 2.49 | -15.84 | -41.08 | 3.71 | -36.92 | -77.95 | -8.11 | 0.058 |
| *Porites astreoides* | *Microspathodon chrysurus* | 1 | -1.5 | -2.81 | -0.31 | -3.12 | -7.97 | 0.09 | 0.21 | -0.91 | 1.29 | 0.617 |
| *Porites astreoides* | *Ophioblennius macclurei* | 1 | -0.13 | -3.11 | 2.83 | 1.81 | -45.26 | 42.08 | -31.92 | -70.65 | -0.52 | 0.794 |
| *Porites astreoides* | *Scomberomorus regalis* | 1 | -0.13 | -3.15 | 2.74 | 2.43 | -45.83 | 40.27 | -30.95 | -69.35 | 3.04 | 0.788 |
| *Porites astreoides* | *Serranus baldwini* | 1 | -1.53 | -3.12 | 0.58 | -6.48 | -31.55 | -0.2 | -0.14 | -2.47 | 1.17 | 0.013 |
| *Porites astreoides* | *Halichoeres radiatus* | 1 | -0.83 | -1.54 | -0.16 | -0.29 | -1.42 | 0.53 | -0.27 | -0.98 | 0.42 | 0.001 |
| *Porites astreoides* | *Holacanthus tricolor* | 1 | -1.01 | -3.96 | 2.1 | -7.24 | -38.69 | 9.18 | -9.43 | -49.21 | 0.74 | 0.29 |
| *Porites astreoides* | *Lutjanus analis* | 1 | -0.27 | -3.37 | 2.63 | 12.2 | -38.09 | 60.6 | -26.86 | -63.15 | -3.65 | 0.722 |
| *Porites astreoides* | *Mycteroperca bonaci* | 1 | -0.48 | -2.1 | 2.24 | 2.33 | -0.07 | 9.76 | -0.07 | -2.19 | 1.71 | 0.463 |
| *Porites astreoides* | *Pseudocaranx dentex* | 1 | 0.83 | -1.29 | 3.46 | 10 | 1.83 | 21.4 | 9.76 | 2.22 | 22.96 | 0.555 |
| *Porites astreoides* | *Bothus ocellatus* | 1 | -0.79 | -3.85 | 1.93 | 41.78 | 11.77 | 79.53 | 1.96 | -5.81 | 11.57 | 0.349 |
| *Porites astreoides* | *Coryphopterus glaucofraenum* | 1 | 0.35 | -1.45 | 2.69 | 6.94 | 0.27 | 15.52 | -0.66 | -2.36 | 0.77 | 0.431 |
| *Porites astreoides* | *Malacanthus plumieri* | 1 | -1.84 | -2.79 | -1.01 | 0.15 | -0.99 | 1 | 0.47 | -0.45 | 1.52 | 0.704 |
| *Porites astreoides* | *Stegastes rocasensis* | 1 | -1.63 | -2.4 | -0.93 | -0.09 | -1.21 | 0.74 | -0.16 | -0.87 | 0.61 | 0.466 |
| *Porites astreoides* | *Apogon americanus* | 1 | -0.09 | -3.18 | 2.88 | -18.9 | -53.77 | 11.63 | -6.86 | -56.76 | 52.04 | 0.68 |
| *Porites astreoides* | *Aluterus scriptus* | 1 | -0.72 | -2.56 | 1.94 | 2.53 | -2.33 | 11.75 | 0.26 | -1.24 | 1.74 | 0.211 |
| *Porites astreoides* | *Melichthys niger* | 1 | -3.69 | -5.85 | -2.1 | -3.87 | -11.34 | 0.12 | -0.29 | -1.28 | 0.8 | 0.381 |
| *Porites astreoides* | *Rypticus saponaceus* | 1 | -0.16 | -3.2 | 2.78 | -0.84 | -51.33 | 44.01 | 30.49 | 4.33 | 68.14 | 0.793 |
| *Porites astreoides* | *Mulloidichthys martinicus* | 1 | 0.83 | -1.38 | 3.39 | 20.91 | 6.96 | 39.92 | -2.06 | -6.73 | 0.55 | 0.498 |
| *Porites astreoides* | *Caranx latus* | 1 | -0.49 | -3.5 | 2.67 | 3.9 | -34.01 | 35.27 | 24.25 | 1.33 | 64.48 | 0.408 |
| *Porites astreoides* | *Diodon hystrix* | 1 | -0.06 | -3.11 | 2.84 | -15.74 | -53.29 | 9.98 | -28.61 | -68.5 | 7.74 | 0.405 |
| *Porites astreoides* | *Cryptotomus roseus* | 1 | -1.07 | -2.09 | -0.24 | -2.31 | -5.84 | -0.03 | 0.33 | -0.43 | 1.11 | 0.001 |
| *Porites astreoides* | *Sparisoma tuiupiranga* | 1 | -0.78 | -1.5 | -0.02 | 0.92 | -0.16 | 2.43 | -0.01 | -0.72 | 0.72 | 0.119 |
| *Porites astreoides* | *Odontoscion dentex* | 1 | -2.9 | -5.72 | 0.51 | -1.73 | -13.79 | 24.32 | 7.83 | 0.73 | 38.8 | 0.502 |
| *Porites astreoides* | *Aluterus monoceros* | 1 | -0.52 | -4.24 | 2.62 | 1.68 | -50.03 | 48.46 | 22.01 | -3.22 | 62.39 | 0.645 |
| *Porites astreoides* | *Haemulon steindachneri* | 1 | -0.58 | -3.76 | 2.39 | 20.79 | -4.63 | 51.64 | 34.23 | 10.29 | 66.17 | 0.483 |
| *Porites astreoides* | *Lactophrys trigonus* | 1 | -0.44 | -3.97 | 2.67 | 0.78 | -52.41 | 45.92 | 19.1 | -8.83 | 60.21 | 0.643 |
| *Porites astreoides* | *Mycteroperca acutirostris* | 1 | -2.39 | -4.85 | -0.53 | -8.1 | -17.44 | -1.68 | 3.04 | 1.13 | 5.77 | 0.45 |
| *Porites astreoides* | *Synodus intermedius* | 1 | 0.09 | -2.89 | 2.98 | -17.87 | -61.03 | 16.46 | 21.95 | -12.22 | 64.09 | 0.742 |
| *Porites astreoides* | *Balistes vetula* | 1 | -0.88 | -4.23 | 2.44 | -22.4 | -62.78 | -0.01 | -8.41 | -35.59 | 17.47 | 0.298 |
| *Porites astreoides* | *Canthigaster figueiredoi* | 1 | -0.59 | -2.55 | 1.64 | -6.44 | -14.97 | -0.45 | 1.9 | 0.15 | 5.31 | 0.001 |
| *Porites astreoides* | *Malacoctenus delalandii* | 1 | -0.23 | -4.06 | 3.12 | -8.53 | -55.25 | 31.78 | 15.82 | -24.06 | 59.26 | 0.613 |
| *Porites astreoides* | *Anisotremus moricandi* | 1 | -1.54 | -5.21 | 2.04 | -0.95 | -32.37 | 21.01 | -4.12 | -45.51 | 13.64 | 0.465 |
| *Porites astreoides* | *Chilomycterus spinosus spinosus* | 1 | -0.01 | -2.81 | 3.02 | -22.94 | -62.22 | 2.1 | -11.11 | -29.62 | 0.47 | 0.13 |
| *Porites astreoides* | *Diplodus argenteus* | 1 | -2.28 | -4.74 | -0.57 | -8.79 | -17.71 | -2.8 | 1.77 | 0.77 | 3.09 | 0.288 |
| *Porites astreoides* | *Sphoeroides spengleri* | 1 | -1.72 | -4.29 | 0.49 | -12.09 | -25.26 | -3.19 | 3.15 | 0.76 | 7.32 | 0.15 |
| *Porites astreoides* | *Dactylopterus volitans* | 1 | -0.38 | -4.15 | 3.01 | -12.6 | -50.79 | 18.03 | 0.47 | -47.94 | 54.21 | 0.53 |
| *Porites astreoides* | *Kyphosus sectatrix* | 1 | 0.18 | -2.73 | 3.2 | -24.19 | -57.74 | -0.04 | -2.81 | -52.56 | 52.07 | 0.566 |
| *Porites astreoides* | *Aulostomus strigosus* | 1 | -0.25 | -3.7 | 2.79 | -16.6 | -55.06 | 10.94 | -9.51 | -63.77 | 46.44 | 0.584 |
| *Porites astreoides* | *Caranx crysos* | 1 | 0.1 | -2.79 | 3.11 | -19.36 | -52.93 | 3.96 | -23 | -69.93 | 42.46 | 0.533 |
| *Porites astreoides* | *Fistularia tabacaria* | 1 | -0.01 | -3.07 | 3.05 | -32.91 | -71.38 | -1.64 | 1.09 | -24.59 | 29.72 | 0.602 |
| *Porites astreoides* | *Chaetodon sedentarius* | 1 | -2.82 | -4.99 | -1.41 | -4.71 | -12.4 | -0.21 | 0.05 | -0.78 | 0.91 | 0.603 |
| *Porites astreoides* | *Priacanthus arenatus* | 1 | -1.95 | -5.62 | 1.61 | 0.51 | -24.7 | 20.36 | -1.07 | -29.81 | 10.54 | 0.449 |
| *Porites astreoides* | *Bodianus pulchellus* | 1 | -2.03 | -3.4 | -1 | -3 | -7.57 | -0.08 | 0.32 | -0.45 | 1.18 | 0.092 |
| *Porites astreoides* | *Elacatinus figaro* | 1 | -0.24 | -3.73 | 2.83 | -15.48 | -54.16 | 12.57 | -12.55 | -58.15 | 42.18 | 0.533 |
| *Porites astreoides* | *Sphoeroides greeleyi* | 1 | -0.22 | -3.79 | 2.88 | -14.05 | -50.89 | 14.06 | -10.63 | -59.73 | 42.39 | 0.525 |
| *Porites astreoides* | *Stephanolepis hispidus* | 1 | -1.77 | -3.21 | -0.78 | -3.48 | -8.61 | -0.26 | 0.35 | -0.36 | 1.15 | 0.012 |
| *Porites astreoides* | *Epinephelus morio* | 1 | -0.88 | -4.18 | 2.53 | -18.46 | -59.53 | 5.86 | 10.66 | 0.22 | 35.24 | 0.235 |
| *Porites astreoides* | *Heteropriacanthus cruentatus* | 1 | -0.32 | -3.57 | 2.7 | -3.84 | -45.9 | 33.58 | -29.66 | -68.23 | 0.76 | 0.47 |
| *Porites astreoides* | *Labrisomus nuchipinnis* | 1 | -0.13 | -3.51 | 3.19 | -28.09 | -66.05 | 0.72 | 7.9 | -16.27 | 35.07 | 0.44 |
| *Porites astreoides* | *Myrichthys ocellatus* | 1 | -0.29 | -3.54 | 2.67 | -3.94 | -45.23 | 33.68 | -30.02 | -68.9 | 0.75 | 0.487 |
| *Porites astreoides* | *Acanthostracion quadricornis* | 1 | -0.61 | -4.25 | 2.55 | 1.85 | -41.71 | 37.15 | -22.82 | -64.21 | 6.45 | 0.391 |
| *Porites astreoides* | *Epinephelus marginatus* | 1 | -3.14 | -5.74 | -1.12 | -6.59 | -15.82 | -0.31 | 4.24 | 1.3 | 10.61 | 0.181 |
| *Porites astreoides* | *Mugil curema* | 1 | 0.35 | -2.26 | 3.01 | 39.44 | 10.73 | 76.02 | -5.35 | -21.08 | 1.45 | 0.595 |
| *Porites astreoides* | *Orthopristis ruber* | 1 | -0.04 | -2.85 | 2.88 | -5.64 | -45.83 | 25.34 | 31.64 | 4.09 | 69.46 | 0.135 |
| *Porites astreoides* | *Kyphosus vaigiensis* | 1 | -2.32 | -6.46 | 2.01 | 5.44 | -28.55 | 48.75 | 10.2 | -0.21 | 44.97 | 0.126 |
| *Porites astreoides* | *Mycteroperca interstitialis* | 1 | -0.69 | -4.4 | 2.34 | 16.39 | -19.71 | 56.44 | 15.92 | -0.13 | 48.79 | 0.563 |
| *Porites astreoides* | *Eucinostomus melanopterus* | 1 | -0.81 | -4.25 | 1.88 | 24.78 | -11.09 | 62.64 | 12.16 | 1.1 | 35.74 | 0.59 |
| *Porites astreoides* | *Lutjanus cyanopterus* | 1 | -0.96 | -5.09 | 2.2 | 21.67 | -30.42 | 67.64 | 14.23 | 0.92 | 48.87 | 0.327 |
| *Porites astreoides* | *Calamus penna* | 1 | -0.28 | -3.91 | 2.77 | -14.63 | -51.25 | 11.98 | -13.33 | -60.58 | 45.57 | 0.612 |
| *Porites astreoides* | *Seriola rivoliana* | 1 | -0.14 | -3.53 | 2.87 | -14.16 | -50.83 | 12.34 | -13.8 | -64.58 | 37.9 | 0.608 |
| *Porites astreoides* | *Parablennius pilicornis* | 1 | -0.36 | -3.77 | 2.69 | -0.94 | -51.83 | 43.7 | 20.9 | -10.35 | 63.34 | 0.713 |
| *Porites astreoides* | *Pareques acuminatus* | 1 | -0.22 | -3.49 | 2.76 | -1.73 | -50.79 | 43.41 | 21.15 | -12.07 | 59.53 | 0.698 |
| *Porites astreoides* | *Rhomboplites aurorubens* | 1 | -1.71 | -5.69 | 1.89 | 9.99 | -6.72 | 32.42 | 2.42 | -0.41 | 7.06 | 0.557 |
| *Porites astreoides* | *Sphoeroides testudineus* | 1 | -0.11 | -3.16 | 2.87 | -3.18 | -50.63 | 36 | 23.68 | -19 | 66.41 | 0.802 |
| *Porites astreoides* | *Myripristis jacobus* | 1 | 0.03 | -2.51 | 2.67 | 43.8 | 12.9 | 80.25 | 5.95 | -1.5 | 16.13 | 0.6 |
| *Porites astreoides* | *Acanthurus bahianus* | 2 | -0.73 | -1.85 | 0.78 | -1.36 | -4.88 | 0.53 | 1.43 | 0.06 | 3.9 | 0.009 |
| *Porites astreoides* | *Acanthurus chirurgus* | 2 | -0.97 | -2.73 | 0.96 | 10.69 | 2.35 | 22.73 | -2.54 | -4.5 | -1.08 | 0.156 |
| *Porites astreoides* | *Sparisoma frondosum* | 2 | -0.33 | -1.67 | 1.67 | 0.5 | -2.98 | 7.24 | -1.38 | -4.32 | 0.01 | 0.478 |
| *Porites astreoides* | *Sparisoma axillare* | 2 | -0.46 | -3.65 | 2.8 | -23.4 | -59.57 | 1.07 | -15.42 | -38.69 | -0.55 | 0.383 |
| *Porites astreoides* | *Sparisoma radians* | 2 | -1.33 | -2.54 | -0.25 | -2.27 | -6.46 | 0.11 | -0.66 | -1.64 | 0.15 | 0.418 |
| *Porites astreoides* | *Stegastes variabilis* | 2 | 0.06 | -1.7 | 2.8 | 3.37 | -0.06 | 11.45 | 0.81 | -1.32 | 1.76 | 0.593 |
| *Porites astreoides* | *Abudefduf saxatilis* | 2 | 1.74 | 0.58 | 3.45 | 1.42 | -0.24 | 5.65 | 1.11 | 0.2 | 2.03 | 0.03 |
| *Porites astreoides* | *Acanthurus coeruleus* | 2 | -1.43 | -2.42 | -0.53 | 0.89 | -0.14 | 2.34 | -0.77 | -1.71 | 0.05 | 0.069 |
| *Porites astreoides* | *Chromis multilineata* | 2 | 0.98 | -0.92 | 3.87 | 2.4 | -3.32 | 11.99 | 0.21 | -1.27 | 1.78 | 0.031 |
| *Porites astreoides* | *Halichoeres brasiliensis* | 2 | -2.57 | -5.2 | 1 | -8.05 | -41.56 | 0.39 | 0.13 | -7.97 | 4.92 | 0.439 |
| *Porites astreoides* | *Halichoeres poeyi* | 2 | -0.79 | -1.73 | 0.02 | -2.91 | -6.31 | -0.48 | 0.33 | -0.36 | 1.05 | 0.007 |
| *Porites astreoides* | *Holacanthus tricolor* | 2 | -0.21 | -3.22 | 2.66 | 1.93 | -46.26 | 43.36 | -29.26 | -68.35 | 5.59 | 0.773 |
| *Porites astreoides* | *Microspathodon chrysurus* | 2 | -0.69 | -4.12 | 2.28 | 16.52 | -24.84 | 60.74 | -23.12 | -62.12 | -0.34 | 0.63 |
| *Porites astreoides* | *Anisotremus virginicus* | 2 | 0.19 | -2.03 | 2.96 | 7.43 | -0.58 | 17.3 | 0.67 | -3.02 | 4.47 | 0.409 |
| *Porites astreoides* | *Haemulon aurolineatum* | 2 | 0.34 | -1.76 | 3.04 | 7.23 | -1.4 | 19.04 | 8.95 | 1.01 | 28.75 | 0.21 |
| *Porites astreoides* | *Stegastes fuscus* | 2 | -0.84 | -1.72 | 0.03 | -0.6 | -2.72 | 0.6 | 1.33 | 0.16 | 2.51 | 0.175 |
| *Porites astreoides* | *Stegastes rocasensis* | 2 | -1.11 | -2.21 | 0.55 | 2.09 | -0.99 | 10.75 | -0.37 | -1.54 | 0.57 | 0.086 |
| *Porites astreoides* | *Thalassoma noronhanum* | 2 | -1.63 | -2.38 | -0.91 | -0.06 | -1.18 | 0.76 | -0.14 | -0.88 | 0.64 | 0.21 |
| *Porites astreoides* | *Halichoeres radiatus* | 2 | -0.04 | -1.53 | 2.26 | -0.64 | -4.28 | 4.76 | -0.06 | -1.31 | 1.19 | 0.236 |
| *Porites astreoides* | *Cantherhines pullus* | 2 | -1.07 | -4.67 | 2.7 | -22.2 | -66.11 | 3.25 | 1.43 | -21.87 | 25.88 | 0.248 |
| *Porites astreoides* | *Cryptotomus roseus* | 2 | -2.8 | -4.98 | -1.28 | -5.04 | -13.05 | -0.34 | 0.7 | -0.28 | 1.86 | 0.029 |
| *Porites astreoides* | *Haemulon plumierii* | 2 | -3.95 | -6.74 | -1.34 | -2.94 | -12.02 | 1.37 | 1.85 | -0.7 | 4.06 | 0.376 |
| *Porites astreoides* | *Canthigaster figueiredoi* | 2 | 0.04 | -3.03 | 3.1 | -32.68 | -73.18 | -0.26 | 5.29 | -18.45 | 32.06 | 0.378 |
| *Porites astreoides* | *Chaetodon striatus* | 2 | -0.65 | -4.26 | 2.41 | 6.76 | -41.05 | 53.68 | 22.76 | 0.09 | 59.94 | 0.319 |
| *Porites astreoides* | *Mycteroperca bonaci* | 2 | -0.47 | -4.09 | 2.63 | 3.42 | -48.55 | 47.57 | 22.1 | -0.7 | 62.68 | 0.641 |
| *Porites astreoides* | *Pomacanthus paru* | 2 | -0.16 | -3.2 | 2.78 | -21.74 | -60.46 | 2.54 | 23.13 | -4.97 | 61.06 | 0.295 |
| *Porites astreoides* | *Scarus zelindae* | 2 | -0.43 | -2.81 | 2.54 | -19.11 | -65.55 | 10.9 | 9.35 | -0.33 | 31.35 | 0.079 |
| *Porites astreoides* | *Sphoeroides spengleri* | 2 | -2.22 | -4.61 | -0.6 | -8.01 | -16.91 | -1.93 | 1.67 | 0.41 | 3.62 | 0.109 |
| *Porites astreoides* | *Ocyurus chrysurus* | 2 | -0.25 | -3.83 | 2.8 | -8.81 | -55.26 | 33.85 | 17.08 | -26.42 | 63.12 | 0.62 |
| *Porites astreoides* | *Labrisomus nuchipinnis* | 2 | -0.36 | -3.72 | 2.63 | 7.95 | -43.27 | 52.97 | 23.85 | -0.51 | 60.46 | 0.685 |
| *Porites astreoides* | *Bodianus pulchellus* | 2 | -0.03 | -3.26 | 2.94 | -19.61 | -60.41 | 8.84 | 26.36 | -2.38 | 63.77 | 0.637 |
| *Porites astreoides* | *Chilomycterus spinosus spinosus* | 2 | -0.3 | -3.91 | 2.96 | -13.03 | -47.89 | 13.79 | -6.49 | -57.82 | 52.57 | 0.528 |
| *Porites astreoides* | *Holocentrus adscensionis* | 2 | -0.14 | -3.21 | 2.82 | -18.97 | -55.19 | 5.99 | -22.76 | -61.93 | 24.38 | 0.294 |
| *Porites astreoides* | *Pseudupeneus maculatus* | 2 | -2.68 | -5.04 | -0.44 | -6.88 | -24.04 | -0.09 | 0.84 | -0.77 | 2.29 | 0.428 |
| *Porites astreoides* | *Serranus baldwini* | 2 | -1.32 | -3.18 | 1.55 | -10.21 | -52.9 | -0.2 | -2.23 | -17.06 | 1.06 | 0.444 |
| *Porites astreoides* | *Stephanolepis hispidus* | 2 | -3.05 | -5.27 | -1.63 | -4.39 | -12.32 | -0.09 | 0.07 | -0.8 | 0.99 | 0.411 |
| *Porites astreoides* | *Bodianus rufus* | 2 | -2.21 | -4.12 | 0.6 | -0.29 | -10.23 | 27 | 5.6 | -0.3 | 34.65 | 0.486 |
| *Porites astreoides* | *Diplodus argenteus* | 2 | -0.04 | -2.97 | 2.94 | -31.77 | -68.61 | -2.35 | 9.5 | -17.85 | 36.98 | 0.471 |
| *Porites astreoides* | *Halichoeres dimidiatus* | 2 | -0.97 | -4.41 | 2.29 | -2.1 | -33.33 | 32.71 | -30.89 | -73.73 | 0.19 | 0.119 |
| *Porites astreoides* | *Sparisoma tuiupiranga* | 2 | -0.3 | -3.48 | 2.72 | -1.39 | -42.08 | 36.08 | -31.85 | -69.12 | -0.42 | 0.186 |
| *Porites astreoides* | *Eucinostomus melanopterus* | 2 | -0.58 | -4.01 | 2.17 | 31.82 | 1.04 | 67.23 | 8.01 | 1.34 | 16.94 | 0.527 |
| *Porites astreoides* | *Sphoeroides greeleyi* | 2 | -0.35 | -3.65 | 2.56 | 10.01 | -39.87 | 53.47 | 24.42 | 0.15 | 60.81 | 0.678 |
| *Porites astreoides* | *Sphoeroides testudineus* | 2 | -0.28 | -3.33 | 2.67 | 8.2 | -42.87 | 53.78 | 23.87 | -0.46 | 57.86 | 0.705 |
| *Porites astreoides* | *Coryphopterus glaucofraenum* | 2 | -0.25 | -3.33 | 2.6 | 6.29 | -43.94 | 51.86 | 27.73 | 1.21 | 65.2 | 0.628 |
| *Porites astreoides* | *Synodus synodus* | 2 | -0.27 | -3.75 | 2.78 | -1.99 | -55.27 | 44.2 | 18.93 | -16.64 | 61.19 | 0.707 |
| *Porites astreoides* | *Pareques acuminatus* | 2 | -0.21 | -3.39 | 2.84 | 0.05 | -45.34 | 39.61 | 27.06 | -8.79 | 67.63 | 0.789 |
| *Porites astreoides* | *Sparisoma amplum* | 2 | 0.01 | -2.44 | 2.52 | 43.4 | 10.88 | 81.38 | 6.62 | -1.46 | 19.78 | 0.582 |
| *Siderastrea spp* | *Acanthurus chirurgus* | 1 | 1.21 | 0.39 | 2.24 | 1.63 | 0.15 | 3.88 | -0.41 | -1.18 | 0.29 | 0 |
| *Siderastrea spp* | *Chaetodon ocellatus* | 1 | -2.43 | -3.83 | -1.26 | -1.49 | -4.2 | 0.35 | -0.87 | -1.79 | -0.05 | 0.63 |
| *Siderastrea spp* | *Haemulon plumierii* | 1 | -0.36 | -1.26 | 1.08 | -0.52 | -1.98 | 0.77 | -1.06 | -3.77 | 0.07 | 0.045 |
| *Siderastrea spp* | *Halichoeres dimidiatus* | 1 | -0.8 | -2.24 | 1.46 | -1.85 | -6.08 | 1.61 | -3.26 | -10.67 | -0.17 | 0.09 |
| *Siderastrea spp* | *Ocyurus chrysurus* | 1 | -0.48 | -1.47 | 1.03 | 2.03 | 0.08 | 5.72 | -0.32 | -1.11 | 0.47 | 0.004 |
| *Siderastrea spp* | *Pomacanthus arcuatus* | 1 | -2.31 | -3.68 | -1.1 | -1.44 | -3.56 | 0.88 | 1.31 | -0.58 | 2.29 | 0.334 |
| *Siderastrea spp* | *Pseudupeneus maculatus* | 1 | 2.15 | 0.73 | 4.1 | -8.28 | -14.67 | -3.86 | -3.93 | -7.58 | -1.23 | 0.03 |
| *Siderastrea spp* | *Sparisoma axillare* | 1 | 1.69 | 0.91 | 2.64 | -0.24 | -1.12 | 0.78 | -1.09 | -2.27 | -0.08 | 0.001 |
| *Siderastrea spp* | *Sparisoma frondosum* | 1 | 1.23 | 0.55 | 1.99 | -0.82 | -1.73 | -0.02 | -0.16 | -1.04 | 0.61 | 0.03 |
| *Siderastrea spp* | *Thalassoma noronhanum* | 1 | -0.96 | -1.66 | -0.26 | 1.22 | 0.25 | 2.41 | -0.59 | -1.31 | 0.09 | 0 |
| *Siderastrea spp* | *Abudefduf saxatilis* | 1 | 4.89 | 2.59 | 8.2 | 1.79 | -0.95 | 6.33 | -0.25 | -2.64 | 1.47 | 0 |
| *Siderastrea spp* | *Acanthurus bahianus* | 1 | 1.42 | 0.67 | 2.28 | 0.16 | -0.73 | 1.2 | 0.14 | -0.61 | 0.83 | 0.015 |
| *Siderastrea spp* | *Acanthurus coeruleus* | 1 | 1.69 | 0.02 | 4.82 | 3.34 | 0.12 | 9.87 | -1.32 | -2.9 | -0.29 | 0.033 |
| *Siderastrea spp* | *Anisotremus virginicus* | 1 | 3.3 | 1.52 | 5.72 | -3.62 | -6.55 | -1.48 | -3.28 | -6.61 | -0.59 | 0.006 |
| *Siderastrea spp* | *Bodianus rufus* | 1 | 0.05 | -1.25 | 1.43 | -5.26 | -10.56 | -1.9 | -0.44 | -4.51 | 1.41 | 0.011 |
| *Siderastrea spp* | *Carangoides bartholomaei* | 1 | 0 | -1.12 | 1.75 | 1.81 | -0.35 | 6.78 | -0.44 | -1.44 | 0.41 | 0.018 |
| *Siderastrea spp* | *Caranx ruber* | 1 | -0.26 | -3.35 | 2.57 | 36.14 | 8.5 | 70.96 | -15.47 | -40.73 | -2.92 | 0.361 |
| *Siderastrea spp* | *Haemulon aurolineatum* | 1 | 0.77 | 0.01 | 1.57 | -1.52 | -2.82 | -0.45 | 0.02 | -0.84 | 0.83 | 0 |
| *Siderastrea spp* | *Haemulon parra* | 1 | -0.42 | -3.46 | 2.48 | -29.27 | -69.15 | -4.07 | -6.42 | -18.9 | 0.47 | 0.393 |
| *Siderastrea spp* | *Halichoeres bivittatus* | 1 | -0.42 | -3.7 | 2.54 | 2.87 | -28.98 | 33.79 | -37.35 | -80.04 | -6.3 | 0.289 |
| *Siderastrea spp* | *Halichoeres brasiliensis* | 1 | 0.46 | -0.22 | 1.19 | -1.49 | -2.78 | -0.37 | 0.61 | -0.09 | 1.34 | 0 |
| *Siderastrea spp* | *Halichoeres poeyi* | 1 | 2.42 | 0.31 | 5.6 | -7.86 | -17.64 | -2.64 | -1.11 | -6.03 | 2.21 | 0.005 |
| *Siderastrea spp* | *Holacanthus ciliaris* | 1 | -0.32 | -1.34 | 0.88 | -1.95 | -4.49 | -0.12 | -2.74 | -5.53 | -0.41 | 0.077 |
| *Siderastrea spp* | *Holocentrus adscensionis* | 1 | 1.52 | 0.65 | 2.63 | 1.25 | -0.04 | 3.33 | 0.28 | -0.47 | 1.09 | 0 |
| *Siderastrea spp* | *Lutjanus jocu* | 1 | 0.79 | -0.91 | 3.1 | 6.29 | 2.01 | 11.88 | -1.91 | -5.49 | -0.38 | 0.041 |
| *Siderastrea spp* | *Pomacanthus paru* | 1 | 0.42 | -0.37 | 1.84 | 0.64 | -0.53 | 3.51 | 0.03 | -0.7 | 0.77 | 0.006 |
| *Siderastrea spp* | *Scarus trispinosus* | 1 | -0.85 | -2.02 | 1.17 | 1.45 | -0.54 | 5.43 | -1.19 | -3.36 | -0.12 | 0.004 |
| *Siderastrea spp* | *Scarus zelindae* | 1 | -0.3 | -0.96 | 0.39 | -0.69 | -1.89 | 0.27 | -0.41 | -1.38 | 0.37 | 0.001 |
| *Siderastrea spp* | *Sparisoma amplum* | 1 | -0.56 | -1.71 | 1.29 | 0.06 | -1.71 | 3.07 | 0.22 | -0.85 | 1.34 | 0.205 |
| *Siderastrea spp* | *Sparisoma radians* | 1 | 0.04 | -2.87 | 2.97 | -36.9 | -76.42 | -7.69 | -6.66 | -21.49 | 4.68 | 0.665 |
| *Siderastrea spp* | *Sphyraena barracuda* | 1 | -2.7 | -5.57 | 0.87 | 0.71 | -8.22 | 21.62 | -10.61 | -48.98 | -0.2 | 0.135 |
| *Siderastrea spp* | *Stegastes variabilis* | 1 | -0.43 | -2.98 | 2.5 | -27.43 | -73.64 | -0.6 | -2.42 | -16.91 | 5.5 | 0.14 |
| *Siderastrea spp* | *Chaetodon striatus* | 1 | 3.47 | 1.1 | 6.91 | -6.85 | -15 | -1.9 | -3.27 | -8.63 | 0.97 | 0 |
| *Siderastrea spp* | *Lutjanus apodus* | 1 | -0.41 | -4.04 | 2.78 | 9.67 | -33.98 | 58.12 | -26.39 | -63.06 | -2.15 | 0.761 |
| *Siderastrea spp* | *Stegastes fuscus* | 1 | 1.02 | 0.32 | 1.76 | -1.24 | -2.33 | -0.28 | 0.6 | -0.1 | 1.32 | 0.021 |
| *Siderastrea spp* | *Acanthostracion polygonius* | 1 | 0.33 | -2.27 | 3.09 | 14 | -3.98 | 38.07 | -40.47 | -76.27 | -11.79 | 0.35 |
| *Siderastrea spp* | *Amblycirrhitus pinos* | 1 | -0.96 | -4.82 | 2.52 | 2.78 | -24.45 | 30.74 | -28.36 | -66 | 0.36 | 0.456 |
| *Siderastrea spp* | *Anisotremus surinamensis* | 1 | 0.06 | -2.9 | 3.07 | -38.86 | -77.22 | -6.98 | 6.41 | -11.81 | 24.71 | 0.304 |
| *Siderastrea spp* | *Aulostomus maculatus* | 1 | -0.27 | -3.37 | 2.74 | -2.03 | -42.07 | 29.61 | -31.86 | -69.5 | 1.12 | 0.723 |
| *Siderastrea spp* | *Cantherhines pullus* | 1 | -1.33 | -2.11 | -0.61 | -0.74 | -2.19 | 0.35 | -0.24 | -0.99 | 0.46 | 0.1 |
| *Siderastrea spp* | *Canthidermis maculata* | 1 | -1.76 | -2.82 | -0.75 | 0.42 | -1.15 | 1.97 | -0.17 | -1.1 | 0.82 | 0.216 |
| *Siderastrea spp* | *Cephalopholis fulva* | 1 | -1.89 | -3.21 | -0.86 | -2.29 | -5.19 | -0.26 | -0.31 | -1.08 | 0.44 | 0.187 |
| *Siderastrea spp* | *Chromis multilineata* | 1 | 0.1 | -0.85 | 1.19 | -1.97 | -4.03 | -0.36 | 0.8 | -0.26 | 2.07 | 0.006 |
| *Siderastrea spp* | *Clepticus brasiliensis* | 1 | -0.53 | -3.37 | 2.52 | -26.4 | -67.75 | -0.57 | 1.7 | -14.97 | 19.35 | 0.349 |
| *Siderastrea spp* | *Epinephelus adscensionis* | 1 | -1.22 | -2.22 | -0.23 | -0.29 | -1.87 | 1.18 | 0.34 | -0.68 | 1.43 | 0.679 |
| *Siderastrea spp* | *Haemulon squamipinna* | 1 | -1.67 | -2.85 | -0.3 | -0.03 | -1.67 | 1.62 | -0.87 | -4.73 | 0.53 | 0.405 |
| *Siderastrea spp* | *Halichoeres penrosei* | 1 | -0.78 | -3.8 | 2.04 | -15.62 | -34.9 | -2.39 | -34.95 | -70.98 | -7.47 | 0.046 |
| *Siderastrea spp* | *Microspathodon chrysurus* | 1 | -2.19 | -4.61 | 0.21 | -7.42 | -30.41 | -0.78 | 0.24 | -3.12 | 4.48 | 0.558 |
| *Siderastrea spp* | *Ophioblennius macclurei* | 1 | -0.16 | -3.17 | 2.75 | -4.21 | -44.64 | 30.47 | -30.58 | -70.25 | 12.81 | 0.804 |
| *Siderastrea spp* | *Scomberomorus regalis* | 1 | -0.19 | -3.25 | 2.76 | -8.33 | -50.54 | 27.4 | -27 | -66.66 | 18.7 | 0.786 |
| *Siderastrea spp* | *Serranus baldwini* | 1 | -1.1 | -2.06 | -0.16 | -0.85 | -2.51 | 0.48 | 0.08 | -0.75 | 0.98 | 0.02 |
| *Siderastrea spp* | *Halichoeres radiatus* | 1 | -0.81 | -1.58 | -0.05 | 1.65 | 0.53 | 3.02 | -0.14 | -0.9 | 0.66 | 0.002 |
| *Siderastrea spp* | *Holacanthus tricolor* | 1 | -0.38 | -2.64 | 3.13 | 0.6 | -5.39 | 8.98 | -3.75 | -21.93 | 0.68 | 0.315 |
| *Siderastrea spp* | *Lutjanus analis* | 1 | -0.3 | -3.57 | 2.77 | 9.5 | -35.41 | 52.96 | -27.97 | -65.37 | -1.94 | 0.754 |
| *Siderastrea spp* | *Mycteroperca bonaci* | 1 | 0.25 | -1.82 | 3.33 | 1.33 | -4.69 | 8.96 | -1.02 | -5.38 | 1.1 | 0.427 |
| *Siderastrea spp* | *Pseudocaranx dentex* | 1 | 0.72 | -1.47 | 3.63 | 3.89 | -0.24 | 10.02 | 10.96 | 2.09 | 27.23 | 0.525 |
| *Siderastrea spp* | *Bothus ocellatus* | 1 | -0.25 | -3.5 | 2.72 | 28.45 | -25.06 | 74.97 | 15.81 | 0.07 | 51.4 | 0.574 |
| *Siderastrea spp* | *Coryphopterus glaucofraenum* | 1 | 0.49 | -1.76 | 3.68 | 2.65 | -1.32 | 9.98 | -0.15 | -1.84 | 1.25 | 0.519 |
| *Siderastrea spp* | *Malacanthus plumieri* | 1 | -2.63 | -4.25 | -1.4 | 2.29 | 1 | 4.03 | 1.65 | 0.13 | 3.64 | 0.774 |
| *Siderastrea spp* | *Stegastes rocasensis* | 1 | -1.96 | -3.04 | -1.06 | 2.16 | 0.92 | 3.59 | 0.14 | -0.9 | 1.28 | 0.448 |
| *Siderastrea spp* | *Apogon americanus* | 1 | -0.36 | -3.56 | 2.49 | 33.95 | 4.94 | 70.21 | -0.23 | -35.51 | 33.48 | 0.644 |
| *Siderastrea spp* | *Aluterus scriptus* | 1 | -0.27 | -3.82 | 2.84 | 38.56 | 5.11 | 76.41 | 5.4 | -3.41 | 18.94 | 0.143 |
| *Siderastrea spp* | *Melichthys niger* | 1 | -3.94 | -6.26 | -2.29 | 2.13 | 0.79 | 3.91 | 0.36 | -1.16 | 2.12 | 0.408 |
| *Siderastrea spp* | *Rypticus saponaceus* | 1 | -0.04 | -3.01 | 2.94 | 6.31 | -16.37 | 34.12 | 36.83 | 8.16 | 73.35 | 0.814 |
| *Siderastrea spp* | *Mulloidichthys martinicus* | 1 | -1.33 | -3.89 | 1.88 | 8.54 | 1.18 | 30.55 | 8.99 | -0.41 | 43.37 | 0.501 |
| *Siderastrea spp* | *Caranx latus* | 1 | -0.45 | -3.52 | 2.44 | -4.9 | -33.03 | 26.19 | 32.77 | 2.5 | 67.55 | 0.469 |
| *Siderastrea spp* | *Diodon hystrix* | 1 | 0.31 | -2.39 | 3.08 | -9.52 | -36.37 | 12.03 | -33.67 | -69.68 | 0.34 | 0.423 |
| *Siderastrea spp* | *Cryptotomus roseus* | 1 | -0.78 | -1.52 | -0.09 | -0.71 | -2.02 | 0.25 | 0.25 | -0.46 | 1.06 | 0.002 |
| *Siderastrea spp* | *Sparisoma tuiupiranga* | 1 | -0.83 | -1.57 | -0.16 | 0.01 | -1.11 | 1.01 | -0.1 | -0.79 | 0.63 | 0.109 |
| *Siderastrea spp* | *Odontoscion dentex* | 1 | -3.36 | -6.39 | -0.87 | -3.94 | -11.37 | 1.01 | 4.77 | 0.53 | 26.09 | 0.565 |
| *Siderastrea spp* | *Aluterus monoceros* | 1 | -0.12 | -3.13 | 2.81 | 26.85 | -29.2 | 71.82 | 13.49 | -2.19 | 43.46 | 0.666 |
| *Siderastrea spp* | *Haemulon steindachneri* | 1 | -1.38 | -4.66 | 1.56 | 4.65 | -13.14 | 27.02 | 33.72 | 8.45 | 69.81 | 0.498 |
| *Siderastrea spp* | *Lactophrys trigonus* | 1 | -0.04 | -3.03 | 2.83 | 25.33 | -33.49 | 74.23 | 13.93 | -2.07 | 45.63 | 0.651 |
| *Siderastrea spp* | *Mycteroperca acutirostris* | 1 | -1.21 | -2.92 | 0.56 | -6.05 | -23.8 | -0.7 | 5.78 | 1 | 25.84 | 0.332 |
| *Siderastrea spp* | *Synodus intermedius* | 1 | -0.02 | -2.87 | 2.8 | -20 | -56.54 | 5.39 | 27.91 | 2.47 | 64.94 | 0.738 |
| *Siderastrea spp* | *Balistes vetula* | 1 | -0.35 | -3.53 | 3.05 | -25.07 | -65.85 | 0.5 | 2.33 | -26.09 | 35.55 | 0.284 |
| *Siderastrea spp* | *Canthigaster figueiredoi* | 1 | 0.49 | -0.99 | 3.16 | -1.35 | -4.18 | 1.37 | 1.76 | -0.01 | 5.37 | 0.002 |
| *Siderastrea spp* | *Malacoctenus delalandii* | 1 | -0.04 | -3.29 | 3.05 | -4.55 | -54.15 | 42.58 | 25.69 | -12.76 | 68.32 | 0.638 |
| *Siderastrea spp* | *Anisotremus moricandi* | 1 | -1.09 | -4.81 | 2.49 | -12.09 | -46.5 | 6.78 | -6.31 | -58.17 | 47.67 | 0.337 |
| *Siderastrea spp* | *Chilomycterus spinosus spinosus* | 1 | 1.22 | -1.7 | 4.47 | 1.65 | -11.39 | 12.72 | -3.8 | -16.89 | 1.28 | 0.176 |
| *Siderastrea spp* | *Diplodus argenteus* | 1 | -0.79 | -1.7 | 0 | -2.23 | -4.32 | -0.67 | 1.41 | 0.55 | 2.43 | 0.285 |
| *Siderastrea spp* | *Sphoeroides spengleri* | 1 | -0.28 | -1.9 | 1.86 | -4.13 | -9.12 | -1.09 | 2.69 | 0.42 | 7.69 | 0.208 |
| *Siderastrea spp* | *Dactylopterus volitans* | 1 | -0.28 | -3.79 | 2.85 | -17.72 | -52.86 | 6.08 | 2.42 | -51.28 | 59.4 | 0.517 |
| *Siderastrea spp* | *Kyphosus sectatrix* | 1 | -0.02 | -3.04 | 2.94 | -21.93 | -53.55 | -1.3 | -5.94 | -58.09 | 52.86 | 0.546 |
| *Siderastrea spp* | *Aulostomus strigosus* | 1 | -0.24 | -3.54 | 2.9 | -19.45 | -57.57 | 4.09 | -6.03 | -63.3 | 55.34 | 0.588 |
| *Siderastrea spp* | *Caranx crysos* | 1 | 1.19 | -1.4 | 4.11 | 3.64 | -8.46 | 16.21 | -11.84 | -63.66 | 33.41 | 0.591 |
| *Siderastrea spp* | *Fistularia tabacaria* | 1 | -0.02 | -2.89 | 2.79 | -33.66 | -68.5 | -5.75 | -4.97 | -35.76 | 31.86 | 0.611 |
| *Siderastrea spp* | *Chaetodon sedentarius* | 1 | -1.88 | -2.77 | -1.08 | -0.5 | -1.97 | 0.63 | -0.01 | -0.82 | 0.87 | 0.599 |
| *Siderastrea spp* | *Priacanthus arenatus* | 1 | -2.65 | -6.04 | 1.7 | -6.9 | -32.05 | 0.96 | -4.01 | -47.38 | 27.48 | 0.325 |
| *Siderastrea spp* | *Bodianus pulchellus* | 1 | -1.71 | -2.64 | -0.89 | -1.29 | -3.29 | 0.11 | 0.24 | -0.49 | 1.08 | 0.102 |
| *Siderastrea spp* | *Elacatinus figaro* | 1 | -0.27 | -3.27 | 2.56 | 37.73 | 8.58 | 76.05 | -0.1 | -32.19 | 22.86 | 0.608 |
| *Siderastrea spp* | *Sphoeroides greeleyi* | 1 | -0.22 | -3.04 | 2.58 | 37.55 | 9.17 | 77.65 | -0.62 | -27.08 | 21.26 | 0.618 |
| *Siderastrea spp* | *Stephanolepis hispidus* | 1 | -1.25 | -2.03 | -0.55 | -0.98 | -2.46 | 0.14 | 0.26 | -0.47 | 1.06 | 0.015 |
| *Siderastrea spp* | *Epinephelus morio* | 1 | -0.13 | -3.39 | 3.16 | -27.92 | -67.15 | -0.59 | 15.8 | -4.96 | 44.5 | 0.201 |
| *Siderastrea spp* | *Heteropriacanthus cruentatus* | 1 | -0.25 | -3.49 | 2.77 | -7.98 | -48.09 | 26.28 | -30.67 | -70.41 | 5.5 | 0.468 |
| *Siderastrea spp* | *Labrisomus nuchipinnis* | 1 | 0 | -2.96 | 2.93 | -32.29 | -69.01 | -2.34 | 0.37 | -29.84 | 35.69 | 0.425 |
| *Siderastrea spp* | *Myrichthys ocellatus* | 1 | -0.37 | -3.6 | 2.73 | -8.73 | -47.52 | 24.97 | -29.2 | -67.15 | 7.93 | 0.474 |
| *Siderastrea spp* | *Acanthostracion quadricornis* | 1 | -0.24 | -3.25 | 2.64 | 35.58 | -1.62 | 75.07 | -1.35 | -31.74 | 20.53 | 0.498 |
| *Siderastrea spp* | *Epinephelus marginatus* | 1 | -3.89 | -6.91 | -1.59 | -6.74 | -13.35 | -1.9 | 3.25 | 1.01 | 6.17 | 0.23 |
| *Siderastrea spp* | *Mugil curema* | 1 | -0.3 | -3.68 | 2.69 | 10.19 | -39.35 | 54.88 | -27.74 | -70.73 | 0.25 | 0.411 |
| *Siderastrea spp* | *Orthopristis ruber* | 1 | -0.27 | -3.43 | 2.78 | -17.03 | -52.66 | 6.5 | 30.62 | 0.57 | 68.99 | 0.157 |
| *Siderastrea spp* | *Kyphosus vaigiensis* | 1 | -3.02 | -6.92 | 1.44 | -0.76 | -37.94 | 35.78 | 9.18 | -0.71 | 43.2 | 0.162 |
| *Siderastrea spp* | *Mycteroperca interstitialis* | 1 | -1.11 | -5.03 | 2.38 | 4.55 | -35.45 | 44.87 | 22.56 | -0.76 | 60.62 | 0.508 |
| *Siderastrea spp* | *Eucinostomus melanopterus* | 1 | -0.66 | -4.66 | 2.55 | 10.93 | -38.61 | 54.95 | 23.83 | 0.88 | 59.88 | 0.567 |
| *Siderastrea spp* | *Lutjanus cyanopterus* | 1 | -1.44 | -6.21 | 2.38 | 3.37 | -43.48 | 50.11 | 18.33 | -0.34 | 54.09 | 0.442 |
| *Siderastrea spp* | *Calamus penna* | 1 | -0.23 | -3.54 | 2.92 | -17.82 | -50.87 | 2.76 | -5.68 | -59.94 | 59.74 | 0.613 |
| *Siderastrea spp* | *Seriola rivoliana* | 1 | -0.25 | -3.69 | 2.71 | -17.72 | -52.77 | 2.89 | -16.81 | -67.9 | 41.77 | 0.576 |
| *Siderastrea spp* | *Parablennius pilicornis* | 1 | -0.27 | -3.62 | 2.88 | -11.78 | -57.22 | 29.39 | 20.88 | -21.48 | 61.76 | 0.72 |
| *Siderastrea spp* | *Pareques acuminatus* | 1 | -0.32 | -3.78 | 2.76 | -15.88 | -61.17 | 24.66 | 13.24 | -28.34 | 55.52 | 0.714 |
| *Siderastrea spp* | *Rhomboplites aurorubens* | 1 | -4.12 | -6.82 | -2.11 | -1.98 | -7.04 | 0.85 | 0.99 | -0.69 | 3.23 | 0.406 |
| *Siderastrea spp* | *Sphoeroides testudineus* | 1 | -0.13 | -3.08 | 2.79 | -11.21 | -56.11 | 22.92 | 23.63 | -24.54 | 66.57 | 0.801 |
| *Siderastrea spp* | *Myripristis jacobus* | 1 | -0.19 | -3.37 | 3 | -17.77 | -61.44 | 22.07 | 12.49 | -35.32 | 62.06 | 0.784 |
| *Siderastrea spp* | *Acanthurus bahianus* | 2 | -0.46 | -1.71 | 1.84 | -1.36 | -6.41 | 0.56 | 2.04 | 0.11 | 7.8 | 0.01 |
| *Siderastrea spp* | *Acanthurus chirurgus* | 2 | -1.86 | -2.96 | -0.93 | 0.64 | -0.77 | 3.14 | -1.35 | -2.58 | -0.4 | 0.454 |
| *Siderastrea spp* | *Sparisoma frondosum* | 2 | -0.38 | -1.67 | 1.91 | 0.42 | -1.64 | 4.7 | -1.22 | -3.54 | 0 | 0.478 |
| *Siderastrea spp* | *Sparisoma axillare* | 2 | 0.32 | -2.61 | 3.77 | 7.48 | -1.26 | 20.44 | -3.07 | -7.89 | -0.48 | 0.45 |
| *Siderastrea spp* | *Sparisoma radians* | 2 | -2.2 | -4.44 | -0.58 | -5.62 | -11.43 | -1.15 | -1.25 | -2.85 | -0.02 | 0.406 |
| *Siderastrea spp* | *Stegastes variabilis* | 2 | 1.54 | -1.27 | 5.06 | -4.97 | -16.87 | 3.86 | -3.94 | -11.69 | 0.61 | 0.54 |
| *Siderastrea spp* | *Abudefduf saxatilis* | 2 | 1.92 | 0.67 | 3.89 | -0.42 | -2.21 | 1.69 | 0.94 | -0.04 | 1.92 | 0.02 |
| *Siderastrea spp* | *Acanthurus coeruleus* | 2 | -1.31 | -2.31 | -0.33 | 0.51 | -0.88 | 1.99 | -0.96 | -2.04 | -0.07 | 0.102 |
| *Siderastrea spp* | *Chromis multilineata* | 2 | 1.13 | -1.15 | 4.63 | -5.47 | -15.84 | 0.04 | 0.35 | -2.78 | 3.28 | 0.029 |
| *Siderastrea spp* | *Halichoeres brasiliensis* | 2 | -1.29 | -3.85 | 2.36 | -14.44 | -58.51 | 1.39 | 7.54 | -1 | 32.67 | 0.38 |
| *Siderastrea spp* | *Halichoeres poeyi* | 2 | -0.47 | -1.17 | 0.17 | -1.3 | -2.73 | -0.17 | 0.22 | -0.44 | 0.91 | 0.008 |
| *Siderastrea spp* | *Holacanthus tricolor* | 2 | -0.19 | -3.17 | 2.79 | -6.54 | -46.7 | 27.7 | -26.67 | -65.49 | 16.97 | 0.789 |
| *Siderastrea spp* | *Microspathodon chrysurus* | 2 | -0.71 | -4.51 | 2.44 | 9.68 | -32.89 | 50.47 | -23.28 | -55.13 | -0.22 | 0.678 |
| *Siderastrea spp* | *Anisotremus virginicus* | 2 | 0.81 | -1.46 | 3.42 | 23.94 | 3.35 | 55.49 | 17.84 | 0.27 | 44.9 | 0.428 |
| *Siderastrea spp* | *Haemulon aurolineatum* | 2 | -0.01 | -2.37 | 2.5 | 17.62 | 4.46 | 36.91 | 36.94 | 5.9 | 72.56 | 0.101 |
| *Siderastrea spp* | *Stegastes fuscus* | 2 | -0.76 | -1.59 | 0.15 | 0.03 | -1.78 | 2.19 | 2.93 | 0.16 | 10.21 | 0.171 |
| *Siderastrea spp* | *Stegastes rocasensis* | 2 | -1.27 | -2.76 | 1.43 | 5.01 | 0.98 | 19.89 | -0.33 | -2.88 | 1.22 | 0.114 |
| *Siderastrea spp* | *Thalassoma noronhanum* | 2 | -1.92 | -2.94 | -1.01 | 2.13 | 0.89 | 3.65 | 0.13 | -0.86 | 1.25 | 0.227 |
| *Siderastrea spp* | *Halichoeres radiatus* | 2 | 0.62 | -1.54 | 3.67 | 7.01 | 0.59 | 18.25 | 1.38 | -1.5 | 7.54 | 0.127 |
| *Siderastrea spp* | *Cantherhines pullus* | 2 | 0.16 | -3.42 | 3.51 | 5.9 | -25.49 | 37.68 | 4.36 | -23.48 | 33.66 | 0.228 |
| *Siderastrea spp* | *Cryptotomus roseus* | 2 | -1.76 | -2.87 | -0.74 | 0.04 | -2.14 | 0.95 | 1.15 | -0.3 | 2.09 | 0.031 |
| *Siderastrea spp* | *Haemulon plumierii* | 2 | -1.7 | -5.22 | 2.01 | 19.46 | -5.04 | 68.79 | 6.97 | -1.47 | 32.17 | 0.249 |
| *Siderastrea spp* | *Canthigaster figueiredoi* | 2 | 1.09 | -1.45 | 3.87 | 17.93 | -0.19 | 45.93 | 14.59 | 0.09 | 41.26 | 0.431 |
| *Siderastrea spp* | *Chaetodon striatus* | 2 | -0.44 | -3.55 | 2.44 | 4.91 | -39.39 | 47.16 | 30.55 | 1.25 | 69.91 | 0.36 |
| *Siderastrea spp* | *Mycteroperca bonaci* | 2 | -0.16 | -3.16 | 2.66 | 24.8 | -25.26 | 69.38 | 15.68 | -1.74 | 52.11 | 0.647 |
| *Siderastrea spp* | *Pomacanthus paru* | 2 | -0.03 | -2.81 | 2.79 | -20.62 | -51.4 | 1.74 | 29.84 | 3.7 | 64.34 | 0.293 |
| *Siderastrea spp* | *Scarus zelindae* | 2 | -0.93 | -2.6 | 2.85 | 0.1 | -3.48 | 7.5 | 1.44 | -0.58 | 7.84 | 0.155 |
| *Siderastrea spp* | *Sphoeroides spengleri* | 2 | -0.87 | -1.83 | 0.08 | -1.86 | -4.03 | -0.2 | 1.35 | 0.18 | 3.2 | 0.122 |
| *Siderastrea spp* | *Ocyurus chrysurus* | 2 | -0.04 | -3.29 | 3.18 | -6.26 | -57.27 | 44.56 | 19.23 | -18.08 | 60.6 | 0.632 |
| *Siderastrea spp* | *Labrisomus nuchipinnis* | 2 | -0.3 | -3.56 | 2.74 | -3.36 | -51.9 | 36.9 | 25.24 | -10.99 | 63.54 | 0.702 |
| *Siderastrea spp* | *Bodianus pulchellus* | 2 | 0.07 | -2.66 | 2.92 | 21.04 | -0.43 | 50.09 | 31.36 | 4.61 | 66.39 | 0.643 |
| *Siderastrea spp* | *Chilomycterus spinosus spinosus* | 2 | -0.26 | -3.3 | 2.58 | 37.19 | 7.2 | 75.45 | 0.99 | -28.75 | 24.19 | 0.616 |
| *Siderastrea spp* | *Holocentrus adscensionis* | 2 | 0.38 | -2.23 | 3.17 | 14.09 | -4.41 | 38.36 | -32.78 | -70.09 | -0.42 | 0.272 |
| *Siderastrea spp* | *Pseudupeneus maculatus* | 2 | -2.09 | -3.3 | -1.08 | -1.06 | -3.3 | 0.54 | 0.43 | -0.69 | 1.58 | 0.476 |
| *Siderastrea spp* | *Serranus baldwini* | 2 | -0.98 | -2.13 | 2.1 | -1.15 | -5.45 | 0.7 | -0.39 | -3.99 | 1.05 | 0.515 |
| *Siderastrea spp* | *Stephanolepis hispidus* | 2 | -2.17 | -3.14 | -1.31 | -0.35 | -1.77 | 0.67 | 0.05 | -0.8 | 1.02 | 0.424 |
| *Siderastrea spp* | *Bodianus rufus* | 2 | -2.86 | -5.77 | 0.56 | -5.78 | -31.9 | 0.47 | 2.69 | -10.61 | 30.93 | 0.514 |
| *Siderastrea spp* | *Diplodus argenteus* | 2 | 0.08 | -2.83 | 3.05 | -31.6 | -67.05 | -4.53 | 3.11 | -30.08 | 37.11 | 0.436 |
| *Siderastrea spp* | *Halichoeres dimidiatus* | 2 | -0.66 | -3.61 | 2.4 | 1.99 | -25.38 | 31.83 | -30.34 | -71.48 | 0.22 | 0.108 |
| *Siderastrea spp* | *Sparisoma tuiupiranga* | 2 | -0.11 | -2.82 | 2.86 | -0.08 | -34.28 | 34.71 | -37.08 | -75.29 | -5.01 | 0.187 |
| *Siderastrea spp* | *Eucinostomus melanopterus* | 2 | -4.5 | -7.53 | -2.28 | -1.66 | -6.54 | 1.39 | 1.93 | -0.3 | 5.16 | 0.222 |
| *Siderastrea spp* | *Sphoeroides greeleyi* | 2 | -0.31 | -3.66 | 2.77 | -2.79 | -50.78 | 36.09 | 25.01 | -15.16 | 63.43 | 0.715 |
| *Siderastrea spp* | *Sphoeroides testudineus* | 2 | -0.27 | -3.5 | 2.68 | 0.49 | -45.76 | 40.73 | 26.41 | -9.23 | 64.52 | 0.729 |
| *Siderastrea spp* | *Coryphopterus glaucofraenum* | 2 | -0.21 | -3.18 | 2.7 | -9.32 | -58.19 | 32.81 | 23.66 | -20.44 | 64.76 | 0.627 |
| *Siderastrea spp* | *Synodus synodus* | 2 | -0.35 | -4 | 2.85 | -10.36 | -56.6 | 33.91 | 18.63 | -26.19 | 62.32 | 0.723 |
| *Siderastrea spp* | *Pareques acuminatus* | 2 | -0.14 | -3.3 | 2.91 | -12.35 | -54.96 | 20.21 | 17.38 | -29.87 | 62.23 | 0.775 |
| *Siderastrea spp* | *Sparisoma amplum* | 2 | -0.16 | -3.37 | 2.89 | -20.38 | -62.48 | 21.44 | 11.58 | -33.36 | 63.25 | 0.768 |

**Supplementary information file S3: Functional trait space analysis and quality checking**

Analyses of fish functional space were based on the protocol described below. First, we measured the pairwise functional distance between fishes using the Gower distance (‘daisy’ function from Cluster package [1]). We used Principal Coordinate Analysis (PCoA) to represent these distances (in the case quasi-Euclidean distances) in a bi-dimensional ordination space (‘dudi.pco’ function from the ade4 package [2]). We tested the quality of the functional space using the framework designed by Maire, Grenouillet, Brosse, & Villéger [3], which involves evaluating the relationship (and the error) between initial and PCoA projected distances along the inclusion of two to a maximum number of axes (*n*=10 in our case). We found a fairly high correspondence and low levels of error between initial and projected distances when using two axes (Fig. S3.1). Thus, we ran further analyses using the two first PCoA axes, which summarized 48% of trait variance. Finally, we calculated the functional space area using a polygon convex-hull area based on the scores of the two PCoA axes (‘Polygon’ function from sp package [4]). The convex-hull area is delimited by a polygon that connects species at the edges of the PCoA ordination space [5]. The functional space, or volume filled by traits, corresponds to the functional richness of local reef fish assemblages [6]. All analyzes were run in R v.4.1.0 [7].

References

1. Maechler, M., Rousseeuw, P., Struyf, A., Hubert, M., & Hornik, K. *cluster: Cluster Analysis Basics and Extensions*. R package version 2.1.0. (2019)

2. Bougeard, S. & Dray, S. Supervised Multiblock Analysis in R with the ade4 Package. *J. Stat. Softw.* **86**, 1-17; [10.18637/jss.v086.i01](https://doi.org/10.18637/jss.v086.i01) (2018)

3. Maire, E., Grenouillet, G., Brosse, S. & Villéger, S. How many dimensions are needed to accurately assess functional diversity? A pragmatic approach for assessing the quality of functional spaces. *Glob. Ecol. Biogeogr.* **24**, 728-740; [10.1111/geb.12299](https://doi.org/10.1111/geb.12299) (2015)

4. Bivand, R.S., Pebesma, E., & Gomez-Rubio, V. *Applied spatial data analysis with R*. 405 p. (Springer, NY, 2013) <https://asdar-book.org/>

5. Cornwell, W.K., Schwilk, D.W. and Ackerly, D.D. A trait-based test for habitat filtering: convex hull volume. *Ecology* **87**, 1465-1471; [10.1890/0012-9658(2006)87[1465:ATTFHF]2.0.CO;2](https://doi.org/10.1890/0012-9658(2006)87%5b1465:ATTFHF%5d2.0.CO;2) (2006)

6. Villéger, S., Mason, N. W. H. & Mouillot, D. New multidimensional functional diversity indices for a multifaceted framework in functional ecology. *Ecology* **89**, 2290-2301; 10.1890/07-1206.1 (2008)

7. R Core Team. *R: A Language and Environment for Statistical Computing* (R Foundation for Statistical Computing, 2021)

Table S3.1: Traits of the 113 fish species analyzed here. Body size is the maximum body size recorded for the species (Quimbayo et al. 2021), and actual body size represents the estimates taken from video plots (Longo et al. 2019). Group size was considered as an ordered trait in functional space analysis, being solitary (Sol) = 1, pair (Pair) = 2, smallg (small group) = 3, medg (medium group) = 4, largeg (large group) = 5. Trait data were taken from Quimbayo et al. (2021).

| Scientific name | Fish life stage | Family | Actual body size | Maximum body size | Aspect ratio | Trophic level | Group size | Maximum of preferred temperature | Maximum of preferred depth |
| --- | --- | --- | --- | --- | --- | --- | --- | --- | --- |
| *Abudefduf saxatilis* | Adult | Pomacentridae | 14.3 | 23 | 1.54 | 3.8 | smallg | 27.85 | 40 |
| *Abudefduf saxatilis* | Juvenile | Pomacentridae | 7.3 | 23 | 1.54 | 3.8 | smallg | 27.85 | 40 |
| *Acanthostracion polygonius* | Adult | Ostraciidae | 22 | 50 | 0.38 | 2 | sol | 28.84 | 80 |
| *Acanthostracion polygonius* | Juvenile | Ostraciidae | 5 | 50 | 0.38 | 2 | sol | 28.84 | 80 |
| *Acanthostracion quadricornis* | Adult | Ostraciidae | 20 | 55 | 1.37 | 2.7 | sol | 28.48 | 80 |
| *Acanthurus bahianus* | Adult | Acanthuridae | 24.1 | 38.1 | 2.43 | 2 | medg | 29.24 | 40 |
| *Acanthurus bahianus* | Juvenile | Acanthuridae | 6.17 | 38.1 | 2.43 | 2 | medg | 29.24 | 40 |
| *Acanthurus chirurgus* | Adult | Acanthuridae | 21.66 | 39 | 1.87 | 2 | medg | 29.37 | 25 |
| *Acanthurus chirurgus* | Juvenile | Acanthuridae | 7.6 | 39 | 1.87 | 2 | medg | 29.37 | 25 |
| *Acanthurus coeruleus* | Adult | Acanthuridae | 23.92 | 39 | 3.49 | 2 | medg | 29.32 | 40 |
| *Acanthurus coeruleus* | Juvenile | Acanthuridae | 6.08 | 39 | 3.49 | 2 | medg | 29.32 | 40 |
| *Aluterus monoceros* | Adult | Monacanthidae | 40 | 76.2 | 1.54 | 3.8 | pair | 28.58 | 50 |
| *Aluterus scriptus* | Adult | Monacanthidae | 35.27 | 110 | 0.86 | 2.8 | sol | 28.61 | 120 |
| *Amblycirrhitus pinos* | Adult | Cirrhitidae | 10 | 9.5 | 1.25 | 3.2 | sol | 29.29 | 46 |
| *Anisotremus moricandi* | Adult | Haemulidae | 15 | 15.1 | 2.66 | 3.4 | sol | 29.4 | 12 |
| *Anisotremus surinamensis* | Adult | Haemulidae | 30.95 | 76 | 2.22 | 3.6 | smallg | 28.86 | 60 |
| *Anisotremus virginicus* | Adult | Haemulidae | 23.71 | 40.6 | 1.77 | 3.6 | smallg | 29.52 | 40 |
| *Anisotremus virginicus* | Juvenile | Haemulidae | 6.58 | 40.6 | 1.77 | 3.6 | smallg | 29.52 | 40 |
| *Apogon americanus* | Adult | Apogonidae | 10 | 10.1 | 1.09 | 3.4 | sol | 28.18 | 50 |
| *Aulostomus maculatus* | Adult | Aulostomidae | 100 | 100 | 1.24 | 4.3 | smallg | 29.84 | 25 |
| *Aulostomus strigosus* | Adult | Aulostomidae | 46.25 | 75 | 0.9 | 4.2 | smallg | 27.69 | 25 |
| *Balistes vetula* | Adult | Balistidae | 38.33 | 60 | 2.79 | 3.8 | sol | 28.08 | 275 |
| *Bodianus pulchellus* | Adult | Labridae | 22.65 | 28.5 | 1.42 | 3.6 | sol | 29.32 | 120 |
| *Bodianus pulchellus* | Juvenile | Labridae | 10 | 28.5 | 1.42 | 3.6 | sol | 29.32 | 120 |
| *Bodianus rufus* | Adult | Labridae | 24.18 | 40 | 1.62 | 3.7 | sol | 29.37 | 70 |
| *Bodianus rufus* | Juvenile | Labridae | 9.2 | 40 | 1.62 | 3.7 | sol | 29.37 | 70 |
| *Bothus ocellatus* | Adult | Bothidae | 15 | 18 | 1.04 | 3.8 | sol | 29.89 | 110 |
| *Calamus penna* | Adult | Sparidae | 26.67 | 46 | 3.65 | 4.4 | medg | 29.52 | 87 |
| *Cantherhines pullus* | Adult | Monacanthidae | 17.02 | 20 | 2.02 | 2.6 | sol | 29.32 | 50 |
| *Cantherhines pullus* | Juvenile | Monacanthidae | 8.58 | 20 | 2.02 | 2.6 | sol | 29.32 | 50 |
| *Canthidermis maculata* | Adult | Balistidae | 28.78 | 50 | 1.81 | 3.5 | largeg | 28.83 | 110 |
| *Canthigaster figueiredoi* | Adult | Tetraodontidae | 10.28 | 12 | 1.33 | 3.1 | pair | 28.67 | 54 |
| *Canthigaster figueiredoi* | Juvenile | Tetraodontidae | 5 | 12 | 1.33 | 3.1 | pair | 28.67 | 54 |
| *Carangoides bartholomaei* | Adult | Carangidae | 34.81 | 100 | 3.61 | 4.5 | largeg | 29.32 | 50 |
| *Caranx crysos* | Adult | Carangidae | 31.5 | 70 | 3.36 | 4.1 | largeg | 26.99 | 100 |
| *Caranx latus* | Adult | Carangidae | 20 | 101 | 3.53 | 4.2 | largeg | 28.83 | 20 |
| *Caranx ruber* | Adult | Carangidae | 38 | 73 | 4.23 | 4.3 | largeg | 29.06 | 35 |
| *Cephalopholis fulva* | Adult | Serranidae | 21.45 | 44 | 1.29 | 4.1 | sol | 28.83 | 70 |
| *Cephalopholis fulva* | NA | Serranidae | NA | 44 | 1.29 | 4.1 | sol | 28.83 | 70 |
| *Chaetodon ocellatus* | Adult | Chaetodontidae | 14.5 | 20 | 2.56 | 3.7 | pair | 29.3 | 30 |
| *Chaetodon sedentarius* | Adult | Chaetodontidae | 12.72 | 15 | 2.11 | 3.9 | pair | 29.08 | 92 |
| *Chaetodon striatus* | Adult | Chaetodontidae | 12.66 | 16 | 2.56 | 3.5 | pair | 29.32 | 35 |
| *Chaetodon striatus* | Juvenile | Chaetodontidae | 4.72 | 16 | 2.56 | 3.5 | pair | 29.32 | 35 |
| *Chaetodon striatus* | NA | Chaetodontidae | NA | 16 | 2.56 | 3.5 | pair | 29.32 | 35 |
| *Chilomycterus spinosus spinosus* | Adult | Diodontidae | 20 | 28 | 0.83 | 3.6 | sol | 25 | 190 |
| *Chilomycterus spinosus spinosus* | Juvenile | Diodontidae | 10 | 28 | 0.83 | 3.6 | sol | 25 | 190 |
| *Chromis multilineata* | Adult | Pomacentridae | 13.59 | 20 | 1.56 | 3 | largeg | 28.22 | 91 |
| *Chromis multilineata* | Juvenile | Pomacentridae | 9.28 | 20 | 1.56 | 3 | largeg | 28.22 | 91 |
| *Clepticus brasiliensis* | Adult | Labridae | 24.38 | 30 | 2.97 | 3.5 | medg | 28.18 | 54 |
| *Coryphopterus glaucofraenum* | Adult | Gobiidae | 7.72 | 8 | 0.67 | 2.7 | smallg | 29.38 | 45 |
| *Coryphopterus glaucofraenum* | Juvenile | Gobiidae | 2.5 | 8 | 0.67 | 2.7 | smallg | 29.38 | 45 |
| *Cryptotomus roseus* | Adult | Scaridae | 11.91 | 13 | 1.11 | 2 | smallg | 29.37 | 30 |
| *Cryptotomus roseus* | Juvenile | Scaridae | 4.88 | 13 | 1.11 | 2 | smallg | 29.37 | 30 |
| *Dactylopterus volitans* | Adult | Dactylopteridae | 30 | 50 | 1.47 | 3.7 | sol | 26.66 | 100 |
| *Diodon hystrix* | Adult | Diodontidae | 35 | 91 | 1.53 | 3.7 | sol | 28.45 | 50 |
| *Diplodus argenteus* | Adult | Sparidae | 17.14 | 37.8 | 3.57 | 3.1 | smallg | 24.78 | 28 |
| *Diplodus argenteus* | Juvenile | Sparidae | 10 | 37.8 | 3.57 | 3.1 | smallg | 24.78 | 28 |
| *Elacatinus figaro* | Adult | Gobiidae | 4 | 3.4 | NA | 3.4 | smallg | 28.18 | 20 |
| *Epinephelus adscensionis* | Adult | Serranidae | 19.74 | 61 | 1.2 | 3.5 | sol | 29.25 | 120 |
| *Epinephelus marginatus* | Adult | Serranidae | 28.35 | 150 | 1.51 | 4.4 | sol | 25.71 | 300 |
| *Epinephelus marginatus* | NA | Serranidae | NA | 150 | 1.51 | 4.4 | sol | 25.71 | 300 |
| *Epinephelus morio* | Adult | Serranidae | 32.73 | 125 | 1.19 | 3.5 | sol | 29.39 | 300 |
| *Eucinostomus melanopterus* | Adult | Gerreidae | 12 | 30 | 2.6 | 3.4 | smallg | 29.77 | 25 |
| *Eucinostomus melanopterus* | Juvenile | Gerreidae | 6.41 | 30 | 2.6 | 3.4 | smallg | 29.77 | 25 |
| *Fistularia tabacaria* | Adult | Fistulariidae | 81 | 200 | NA | 3.7 | sol | 28.69 | 200 |
| *Haemulon aurolineatum* | Adult | Haemulidae | 19.55 | 25 | 2.1 | 4.4 | largeg | 29.39 | 40 |
| *Haemulon aurolineatum* | Juvenile | Haemulidae | 6.08 | 25 | 2.1 | 4.4 | largeg | 29.39 | 40 |
| *Haemulon parra* | Adult | Haemulidae | 21.24 | 41.2 | 2.27 | 3.5 | medg | 29.5 | 40 |
| *Haemulon plumierii* | Adult | Haemulidae | 23.12 | 53 | 3 | 3.8 | medg | 29.39 | 40 |
| *Haemulon plumierii* | Juvenile | Haemulidae | 7.14 | 53 | 3 | 3.8 | medg | 29.39 | 40 |
| *Haemulon squamipinna* | Adult | Haemulidae | 15.59 | 11.5 | 3.41 | 3.4 | largeg | 28.45 | 40 |
| *Haemulon steindachneri* | Adult | Haemulidae | 21.53 | 30 | 1.95 | 3.7 | medg | 28.12 | 30 |
| *Halichoeres bivittatus* | Adult | Labridae | 20.69 | 35 | 1.1 | 3.8 | sol | 29.49 | 15 |
| *Halichoeres bivittatus* | Juvenile | Labridae | 10 | 35 | 1.1 | 3.8 | sol | 29.49 | 15 |
| *Halichoeres brasiliensis* | Adult | Labridae | 25.55 | 39.5 | 0.77 | 3.7 | sol | 28.13 | 35 |
| *Halichoeres brasiliensis* | Juvenile | Labridae | 9.58 | 39.5 | 0.77 | 3.7 | sol | 28.13 | 35 |
| *Halichoeres dimidiatus* | Adult | Labridae | 21.42 | 27 | 0.86 | 3.6 | sol | 28.23 | 60 |
| *Halichoeres dimidiatus* | Juvenile | Labridae | 6 | 27 | 0.86 | 3.6 | sol | 28.23 | 60 |
| *Halichoeres penrosei* | Adult | Labridae | 11.85 | 11.9 | NA | 3.4 | sol | 28.13 | 30 |
| *Halichoeres poeyi* | Adult | Labridae | 18.23 | 20 | 1.48 | 3.7 | sol | 29.4 | 15 |
| *Halichoeres poeyi* | Juvenile | Labridae | 8.68 | 20 | 1.48 | 3.7 | sol | 29.4 | 15 |
| *Halichoeres radiatus* | Adult | Labridae | 22.25 | 51 | 1.5 | 3.5 | sol | 29.71 | 55 |
| *Halichoeres radiatus* | Juvenile | Labridae | 8.75 | 51 | 1.5 | 3.5 | sol | 29.71 | 55 |
| *Heteropriacanthus cruentatus* | Adult | Priacanthidae | 30 | 50.7 | 1.84 | 3.6 | smallg | 28.45 | 300 |
| *Holacanthus ciliaris* | Adult | Pomacanthidae | 26.68 | 45 | 0.8 | 3 | sol | 29.43 | 70 |
| *Holacanthus tricolor* | Adult | Pomacanthidae | 14.78 | 35 | 1.1 | 3 | sol | 29.32 | 92 |
| *Holacanthus tricolor* | Juvenile | Pomacanthidae | 6 | 35 | 1.1 | 3 | sol | 29.32 | 92 |
| *Holocentrus adscensionis* | Adult | Holocentridae | 21.11 | 61 | 2.11 | 3.1 | sol | 28.75 | 180 |
| *Holocentrus adscensionis* | Juvenile | Holocentridae | 10 | 61 | 2.11 | 3.1 | sol | 28.75 | 180 |
| *Holocentrus adscensionis* | NA | Holocentridae | NA | 61 | 2.11 | 3.1 | sol | 28.75 | 180 |
| *Kyphosus sectatrix* | Adult | Kyphosidae | 34.17 | 76 | 2.71 | 2 | medg | 28.35 | 25 |
| *Kyphosus vaigiensis* | Adult | Kyphosidae | 38.64 | 70 | 3.25 | 2 | medg | 28.23 | 25 |
| *Labrisomus nuchipinnis* | Adult | Labrisomidae | 16.75 | 23 | 0.83 | 3.6 | sol | 28.83 | 10 |
| *Labrisomus nuchipinnis* | Juvenile | Labrisomidae | 9 | 23 | 0.83 | 3.6 | sol | 28.83 | 10 |
| *Lactophrys trigonus* | Adult | Ostraciidae | 40 | 55 | 1.27 | 3.3 | sol | 29.38 | 50 |
| *Lutjanus analis* | Adult | Lutjanidae | 35 | 94 | 1.69 | 3.9 | smallg | 29.39 | 95 |
| *Lutjanus apodus* | Adult | Lutjanidae | 15 | 79.1 | 1.78 | 4.3 | smallg | 29.39 | 89 |
| *Lutjanus cyanopterus* | Adult | Lutjanidae | 45 | 160 | 2.25 | 4.4 | smallg | 29.39 | 70 |
| *Lutjanus jocu* | Adult | Lutjanidae | 40.81 | 128 | 1.39 | 4.4 | smallg | 29.44 | 150 |
| *Malacanthus plumieri* | Adult | Malacanthidae | 39.34 | 70 | 1.25 | 3.7 | sol | 29.37 | 153 |
| *Malacoctenus delalandii* | Adult | Labrisomidae | 10 | 8.2 | 1 | 3.4 | sol | 29.32 | 3 |
| *Melichthys niger* | Adult | Balistidae | 29.48 | 50 | 2.28 | 2.4 | largeg | 29.29 | 75 |
| *Microspathodon chrysurus* | Adult | Pomacentridae | 15.33 | 21 | 1.09 | 2.1 | sol | 29.4 | 40 |
| *Microspathodon chrysurus* | Juvenile | Pomacentridae | 6 | 21 | 1.09 | 2.1 | sol | 29.4 | 40 |
| *Microspathodon chrysurus* | NA | Pomacentridae | NA | 21 | 1.09 | 2.1 | sol | 29.4 | 40 |
| *Mugil curema* | Adult | Mugilidae | 40 | 91 | 1.91 | 2 | largeg | 27.39 | 300 |
| *Mulloidichthys martinicus* | Adult | Mullidae | 22.6 | 44.8 | 2.26 | 3.2 | medg | 28.77 | 49 |
| *Mycteroperca acutirostris* | Adult | Serranidae | 33.41 | 80 | 1.13 | 3.4 | sol | 29.45 | 40 |
| *Mycteroperca bonaci* | Adult | Serranidae | 36.44 | 150 | 1.52 | 4.3 | sol | 29.37 | 100 |
| *Mycteroperca bonaci* | Juvenile | Serranidae | 4 | 150 | 1.52 | 4.3 | sol | 29.37 | 100 |
| *Mycteroperca interstitialis* | Adult | Serranidae | 28.25 | 84 | 1.28 | 4.5 | sol | 29.38 | 150 |
| *Myrichthys ocellatus* | Adult | Ophichthidae | 60 | 110 | 0.16 | 3.6 | sol | 29.38 | 7 |
| *Myrichthys ocellatus* | NA | Ophichthidae | NA | 110 | 0.16 | 3.6 | sol | 29.38 | 7 |
| *Myripristis jacobus* | Adult | Holocentridae | 15 | 25 | 2.77 | 3.4 | medg | 28.75 | 210 |
| *Ocyurus chrysurus* | Adult | Lutjanidae | 23.54 | 86.3 | 1.74 | 4 | smallg | 29.3 | 180 |
| *Ocyurus chrysurus* | Juvenile | Lutjanidae | 9.6 | 86.3 | 1.74 | 4 | smallg | 29.3 | 180 |
| *Odontoscion dentex* | Adult | Sciaenidae | 22.29 | 30 | 1.26 | 3.5 | smallg | 29.52 | 30 |
| *Ophioblennius macclurei* | Adult | Blenniidae | 12 | 12.2 | NA | 2.5 | sol | 29.73 | 8 |
| *Orthopristis ruber* | Adult | Haemulidae | 22.5 | 40 | 2.1 | 3.6 | medg | 29.2 | 70 |
| *Parablennius pilicornis* | Adult | Blenniidae | 7 | 12.7 | 1.4 | 3.2 | sol | 24.49 | 25 |
| *Pareques acuminatus* | Adult | Sciaenidae | 20 | 23 | 1.25 | 3.6 | smallg | 29.45 | 110 |
| *Pareques acuminatus* | Juvenile | Sciaenidae | 2.5 | 23 | 1.25 | 3.6 | smallg | 29.45 | 110 |
| *Pomacanthus arcuatus* | Adult | Pomacanthidae | 32.27 | 60 | 2.06 | 3.2 | pair | 29.38 | 30 |
| *Pomacanthus paru* | Adult | Pomacanthidae | 29.86 | 41.1 | 1.83 | 2.8 | pair | 29.38 | 100 |
| *Pomacanthus paru* | Juvenile | Pomacanthidae | 7.08 | 41.1 | 1.83 | 2.8 | pair | 29.38 | 100 |
| *Priacanthus arenatus* | Adult | Priacanthidae | 18.5 | 50 | 2.04 | 4 | medg | 27.61 | 200 |
| *Pseudocaranx dentex* | Adult | Carangidae | 21.75 | 80 | 4.38 | 3.9 | smallg | 26.99 | 238 |
| *Pseudupeneus maculatus* | Adult | Mullidae | 18.35 | 30 | 2.21 | 3.7 | smallg | 29.32 | 90 |
| *Pseudupeneus maculatus* | Juvenile | Mullidae | 9.43 | 30 | 2.21 | 3.7 | smallg | 29.32 | 90 |
| *Pseudupeneus maculatus* | NA | Mullidae | NA | 30 | 2.21 | 3.7 | smallg | 29.32 | 90 |
| *Rhomboplites aurorubens* | Adult | Lutjanidae | 18.64 | 60 | 2.77 | 4.4 | medg | 29.18 | 300 |
| *Rypticus saponaceus* | Adult | Serranidae | 21.67 | 35 | 1.23 | 4.1 | sol | 28.55 | 140 |
| *Scarus trispinosus* | Adult | Scaridae | 39.85 | 55.6 | NA | 2 | smallg | 28.18 | 45 |
| *Scarus zelindae* | Adult | Scaridae | 31.49 | 33.2 | NA | 2 | smallg | 28.18 | 60 |
| *Scarus zelindae* | Juvenile | Scaridae | 7.37 | 33.2 | NA | 2 | smallg | 28.18 | 60 |
| *Scomberomorus regalis* | Adult | Scombridae | 110 | 183 | 4.52 | 4.5 | smallg | 29.83 | 20 |
| *Seriola rivoliana* | Adult | Carangidae | 47 | 160 | 3.74 | 4.5 | smallg | 28.21 | 245 |
| *Serranus baldwini* | Adult | Serranidae | 9.8 | 12 | 1.08 | 4.1 | sol | 28.91 | 80 |
| *Serranus baldwini* | Juvenile | Serranidae | 4.76 | 12 | 1.08 | 4.1 | sol | 28.91 | 80 |
| *Sparisoma amplum* | Adult | Scaridae | 25.84 | 39 | NA | 2 | smallg | 28.18 | 30 |
| *Sparisoma amplum* | Juvenile | Scaridae | 10 | 39 | NA | 2 | smallg | 28.18 | 30 |
| *Sparisoma axillare* | Adult | Scaridae | 24.88 | 43.7 | 1.2 | 2 | smallg | 28.58 | 54 |
| *Sparisoma axillare* | Juvenile | Scaridae | 8.72 | 43.7 | 1.2 | 2 | smallg | 28.58 | 54 |
| *Sparisoma frondosum* | Adult | Scaridae | 22.31 | 34.5 | 1.35 | 2 | smallg | 28.11 | 54 |
| *Sparisoma frondosum* | Juvenile | Scaridae | 8.42 | 34.5 | 1.35 | 2 | smallg | 28.11 | 54 |
| *Sparisoma frondosum* | NA | Scaridae | NA | 34.5 | 1.35 | 2 | smallg | 28.11 | 54 |
| *Sparisoma radians* | Adult | Scaridae | 13.16 | 20 | 1.12 | 2 | smallg | 29.45 | 12 |
| *Sparisoma radians* | Juvenile | Scaridae | 8.71 | 20 | 1.12 | 2 | smallg | 29.45 | 12 |
| *Sparisoma tuiupiranga* | Adult | Scaridae | 16.18 | 15.4 | 1.18 | 2 | smallg | 27.84 | 15 |
| *Sparisoma tuiupiranga* | Juvenile | Scaridae | 4.58 | 15.4 | 1.18 | 2 | smallg | 27.84 | 15 |
| *Sphoeroides greeleyi* | Adult | Tetraodontidae | 13.86 | 18 | 1.75 | 3.5 | sol | 29.86 | NA |
| *Sphoeroides greeleyi* | Juvenile | Tetraodontidae | 10 | 18 | 1.75 | 3.5 | sol | 29.86 | NA |
| *Sphoeroides spengleri* | Adult | Tetraodontidae | 13.95 | 30 | 1.97 | 3.3 | sol | 28.84 | 45 |
| *Sphoeroides spengleri* | Juvenile | Tetraodontidae | 8.95 | 30 | 1.97 | 3.3 | sol | 28.84 | 45 |
| *Sphoeroides testudineus* | Adult | Tetraodontidae | 15 | 38.8 | 1.42 | 3.4 | sol | 29.52 | 10 |
| *Sphoeroides testudineus* | Juvenile | Tetraodontidae | 10 | 38.8 | 1.42 | 3.4 | sol | 29.52 | 10 |
| *Sphyraena barracuda* | Adult | Sphyraenidae | 95.71 | 200 | 2.45 | 4.5 | smallg | 29.39 | 100 |
| *Sphyraena barracuda* | Juvenile | Sphyraenidae | 6 | 200 | 2.45 | 4.5 | smallg | 29.39 | 100 |
| *Stegastes fuscus* | Adult | Pomacentridae | 11.11 | 12.6 | 1.48 | 3.3 | sol | 28.21 | 40 |
| *Stegastes fuscus* | Juvenile | Pomacentridae | 4.5 | 12.6 | 1.48 | 3.3 | sol | 28.21 | 40 |
| *Stegastes rocasensis* | Adult | Pomacentridae | 9.48 | 8.5 | 0.89 | 2.7 | sol | 28.8 | 10 |
| *Stegastes rocasensis* | Juvenile | Pomacentridae | 4.97 | 8.5 | 0.89 | 2.7 | sol | 28.8 | 10 |
| *Stegastes rocasensis* | NA | Pomacentridae | NA | 8.5 | 0.89 | 2.7 | sol | 28.8 | 10 |
| *Stegastes variabilis* | Adult | Pomacentridae | 11.37 | 12.5 | 1.98 | 2 | sol | 28.86 | 30 |
| *Stegastes variabilis* | Juvenile | Pomacentridae | 4.32 | 12.5 | 1.98 | 2 | sol | 28.86 | 30 |
| *Stephanolepis hispidus* | Adult | Monacanthidae | 17.33 | 27.5 | 1.27 | 2.6 | sol | 28.36 | 293 |
| *Stephanolepis hispidus* | Juvenile | Monacanthidae | 8.96 | 27.5 | 1.27 | 2.6 | sol | 28.36 | 293 |
| *Synodus intermedius* | Adult | Synodontidae | 16.25 | 46 | 1.56 | 4.2 | sol | 29.39 | 186 |
| *Synodus synodus* | Juvenile | Synodontidae | 10 | 43 | 1.83 | 4.2 | sol | 28.41 | 295 |
| *Thalassoma noronhanum* | Adult | Labridae | 7.12 | 13.3 | NA | 3.4 | medg | 28.18 | 60 |
| *Thalassoma noronhanum* | Juvenile | Labridae | 4.66 | 13.3 | NA | 3.4 | medg | 28.18 | 60 |


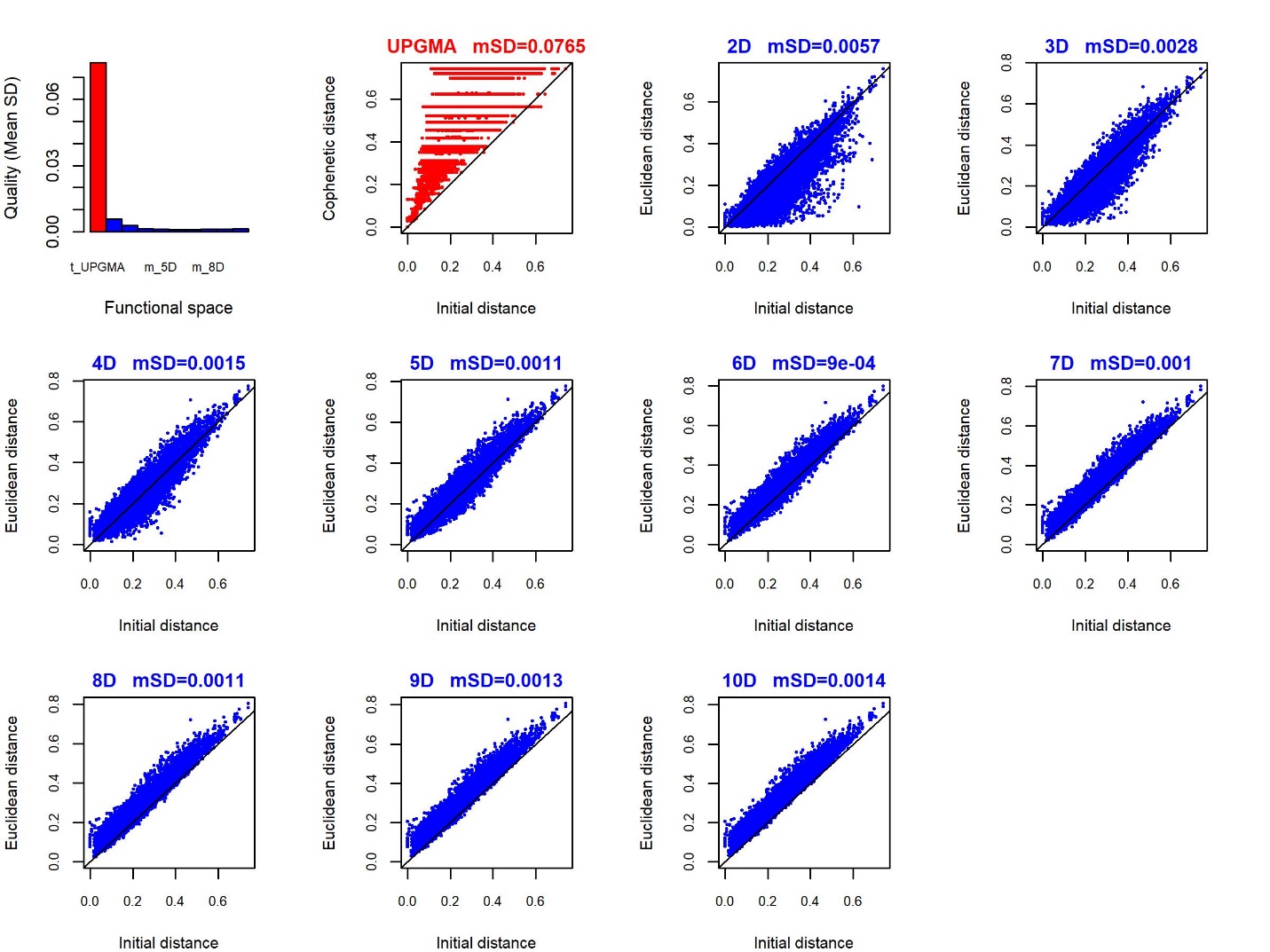


**Fig. S3.1. Plots of functional space quality analysis.**

The plot shows the relationship between initial (X axis) and projected distances (Y axis), along the inclusion of two to ten ordination axes. The error (mSD= mean squared deviation) is shown in the top of all plots. This figure was produced and edited in R v.4.1.2 (https://www.r-project.org/).


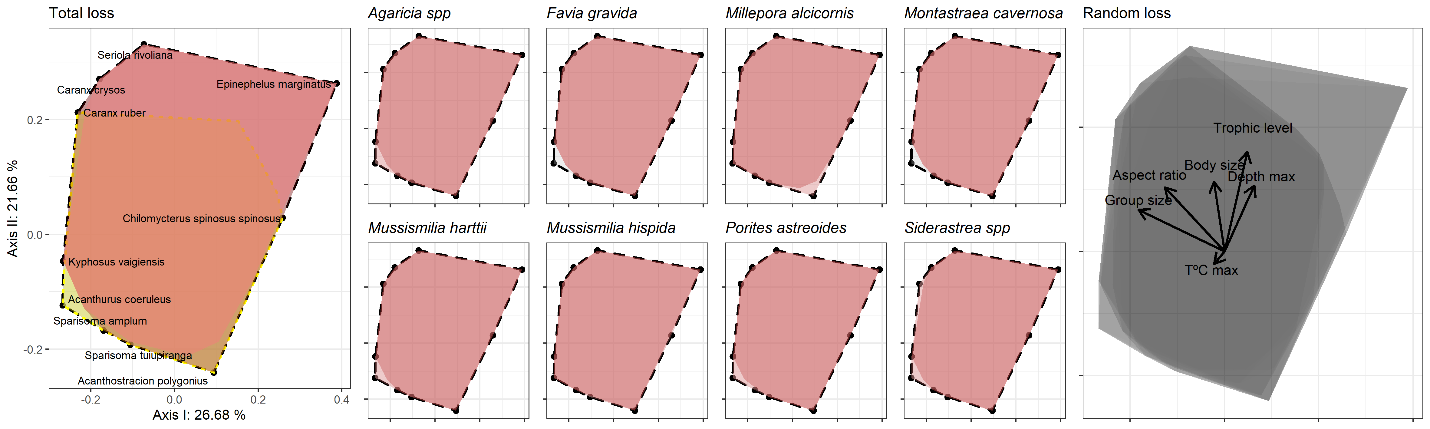


**Figure S3.2. Functional trait spaces based on reef fish of the Brazilian reefs.**

The effect of coral declines on functional space was analyzed by building spaces for the entire assemblage of reef fish of the Brazilian reefs. Functional trait spaces were based on six fish traits (group size, maximum body size, trophic level, aspect ratio, depth range, preferred temperature), with their correlation with PCoA-ordination axes in the right plot. The dashed line represents the complete functional trait space composed by the 113 fish analyzed here. In the left plot, we present the trait space remaining after the simulated loss all coral-associated fish (Total loss); in this plot we also show the identity of the species in the vertices of the trait space, as well the space occupied by ‘all coral-associated fish’ (yellow polygon in the background). In the middle we present the space remaining after the simulated loss of fish associated to a particular coral species (loss per coral species). The space remaining after the loss of adult coral-associate fish is presented in dark red, whereas the space remaining after the loss of juvenile coral-associated fish is presented in light red. In the right plot we present the space remaining after 100 runs of the random loss of fish (100 superimposed random polygons). This figure was produced and edited in R v.4.1.2 (https://www.r-project.org/).


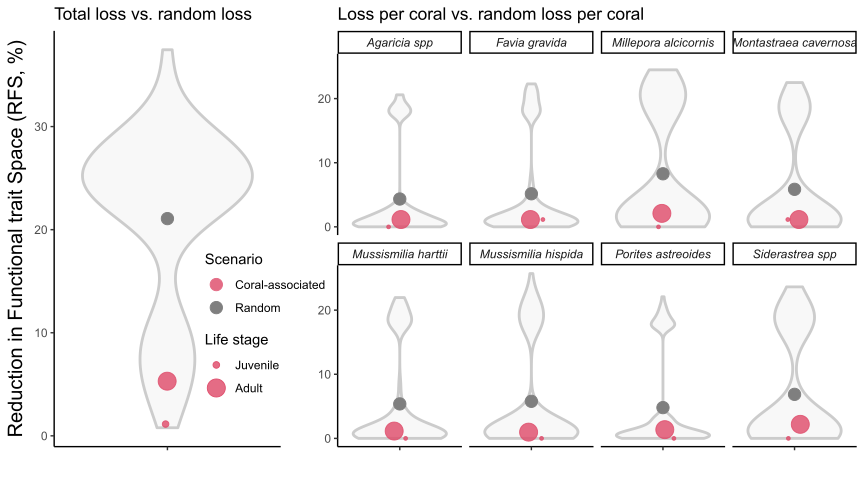


**Fig. S3.3. Reduction in the Functional trait Space area (RFS) between scenarios of loss of coral-associated fish and random loss.**

Random loss was simulated by using an alternative randomization procedure where species in the vertices of the trait space were more likely to be lost. Points depicted the average for each scenario and fish life stage. This figure was produced using R v.4.1.2 (https://www.r-project.org/) and edited using Inkscape v.1.0 (https://inkscape.org/).
